# Responses of turkey vultures to unmanned aircraft systems vary by platform

**DOI:** 10.1038/s41598-021-01098-5

**Published:** 2021-11-04

**Authors:** Morgan B. Pfeiffer, Bradley F. Blackwell, Thomas W. Seamans, Bruce N. Buckingham, Joshua L. Hoblet, Patrice E. Baumhardt, Travis L. DeVault, Esteban Fernández-Juricic

**Affiliations:** 1 U.S. Department of Agriculture, Animal and Plant Inspection Service, Wildlife Services, National Wildlife Research Center, 6100 Columbus Avenue, Sandusky, OH 44870 USA; 2 School of Natural Resource Management, George Campus, Nelson Mandela University, George, South Africa; 3 Department of Biological Sciences, Purdue University, 915 West State Street, West Lafayette, IN 47907 USA; 4 Savannah River Ecology Laboratory, University of Georgia, Aiken, SC 29802 USA

**Keywords:** Animal behaviour, Urban ecology

## Abstract

A challenge that conservation practitioners face is manipulating behavior of nuisance species. The turkey vulture (*Cathartes aura*) can cause substantial damage to aircraft if struck. The goal of this study was to assess vulture responses to unmanned aircraft systems (UAS) for use as a possible dispersal tool. Our treatments included three platforms (fixed-wing, multirotor, and a predator-like ornithopter [powered by flapping flight]) and two approach types (30 m overhead or targeted towards a vulture) in an operational context. We evaluated perceived risk as probability of reaction, reaction time, flight-initiation distance (FID), vulture remaining index, and latency to return. Vultures escaped sooner in response to the fixed-wing; however, fewer remained after multirotor treatments. Targeted approaches were perceived as riskier than overhead. Vulture perceived risk was enhanced by flying the multirotor in a targeted approach. We found no effect of our treatments on FID or latency to return. Latency was negatively correlated with UAS speed, perhaps because slower UAS spent more time over the area. Greatest visual saliency followed as: ornithopter, fixed-wing, and multirotor. Despite its appearance, the ornithopter was not effective at dispersing vultures. Because effectiveness varied, multirotor/fixed-wing UAS use should be informed by management goals (immediate dispersal versus latency).

## Introduction

The use of small (< 25 kg) unmanned aircraft systems (also referred to as unoccupied aircraft systems; hereafter UAS) has generated interest among conservation practitioners because of the potential to save time and money previously allocated to human occupied aircraft operations^1–3^. This technology also has potential to save human lives, as traditional aircraft wildlife operations generally require low-level flights in rugged terrain that can result in aircraft stalls and collisions with structures; in fact, aircraft operations are the leading cause of death for field biologists^4,5^. Adding to the appeal of UAS use are the customizability and potential reduction in disturbance to wildlife compared to traditional aircraft operations^1,2,6^. In addition to passive wildlife management activities (e.g., estimating the number of animals in wildlife populations^1,7^), UAS have potential to be used actively, including use as a nonlethal hazing tool to disperse nuisance wildlife^8,9^. Targeted wildlife dispersal with UAS might reduce local abundances, and thus could prevent wildlife collisions with civil or military aircraft, which can result in human injuries, damage to the aircraft, and animal mortality^10,11^.

For both passive and active UAS operations, understanding wildlife reactions in response to different types of UAS approaches can aid our ability to use this technology more effectively^8^. In comparison to passive wildlife UAS operations, maximizing wildlife dispersal with active UAS operations is not well studied^8,12,13^. Animals generally respond to an approaching object as if they were assessing risk in the context of a cost–benefit model (i.e., the cost of escape compared to the cost associated with loss of foraging and other vital activities)^14,15^. We can apply antipredator theory to the novel risk posed by UAS to wildlife, as wildlife show escape behavior in response to vehicle approach, including aircraft^16,17^.

An animal’s decision to escape from an approaching UAS is likely based on characteristics of the object including form and flight pattern^8,18,19^. In recent studies, UAS that were similar to natural aerial predator forms (e.g., frames with a short neck or raptor-like)^20^ were generally perceived as riskier than multirotor or fixed-wing UAS to multiple bird species^8,18^. Flights with a multirotor system, which ostensibly had the least predator-like form, resulted in close approaches (4 m) to waterbirds before flushing and, in a controlled setting (i.e., animals within an enclosure outdoors), multirotor approaches failed to elicit any escape responses from red-winged blackbirds (*Agelaius phoeniceus*)^6,8^. In terms of direction of approach, target-oriented UAS approaches (both horizontal and vertical) towards wildlife were considered more threatening than overhead flight patterns, likely because of the increased perceived risk of a collision^6,8,19,20^. Previous research investigating UAS platform and approach on avian response used a small passerine species in a controlled setting involving captive birds^8^. Here we expand on that design with a medium-sized raptor in an operational landfill context.

Building upon the antipredator conceptual framework in a vehicle-approach context, we designed an experiment to quantify the reactions of turkey vultures (*Cathartes aura,* hereafter referred to as vultures) in response to approach by UAS platforms (multirotor, fixed-wing, and ornithopter [an UAS engineered to fly with flapping motions]), and direction of approach (overhead or targeted). We selected turkey vultures as our target species because they are medium-sized (2 kg) obligate scavengers and the most widely distributed of the New World vultures^21^. Given their abundance and large body size turkey vultures also pose a known risk to aviation safety across the USA; between January 2015 and August 2020, over 350 strikes with this species resulted in over $19 million in aircraft repair/associated costs in addition to the death of the individual vultures^22,23^. Most vulture collisions occur outside of the airport environment (> 152 m Above Ground Level [AGL] and outside of the airport property) and no deterrents have been evaluated on their ability to alter vulture flight paths^24,25^. Therefore, development of nonlethal management tools and integration with other techniques (e.g., resource management and pyrotechnics) is warranted for reducing bird/aircraft collisions, thus protecting people, property, and vultures.

We hypothesized that approach by the predator ornithopter would be perceived by turkey vultures as riskier (e.g., assessed as a level of risk^26^) than the fixed-wing or multirotor UAS^18^. Although other raptors rarely prey upon turkey vultures, during periods of heightened defense (such as breeding), peregrine falcons (*Falco peregrinus*) can successfully knock turkey vultures out of the air; therefore, raptor presence and, perhaps, silhouettes have potential to elicit a fear response^27,28^. We hypothesized that targeted approaches would be perceived as riskier than overhead approaches because lower UAS flights caused more escape reactions than overhead flights in previous research^29–31^. We hypothesized that certain interactions of UAS platform and approach would increase the perceived risk because of the additive effects of a predator form during a direct approach^18,31^.

We therefore predicted that vultures would react sooner, have longer flight-initiation distances (FID; distance between the UAS and the vulture when escape is initiated), disperse in greater numbers, and have greater latency to return to the study area in response to (1) the ornithopter than the fixed-wing or multirotor; (2) targeted than overhead UAS approaches; and (3) the ornithopter in a targeted approach than the other UAS/approach interactions.

It was impractical to conduct a behavioral experiment with captive vultures and UAS, hence we selected a landfill as a study site because of a high abundance of vultures in free-flying conditions (i.e., without attracting vultures by providing bait). Under these field conditions, vultures could be engaged in a variety of activities (e.g., foraging, socializing, preening, etc.) and occasionally were surrounded by mixed flocks of herring (*Larus argentatus*) and ring-billed gulls (*L. delawarensis*). We considered in our Methods (see below) the possibility the gulls might influence vulture response to UAS approach^14^. Also, we standardized our approach by selecting nonflying vultures on the ground located at a higher vantage point than gulls as focal birds. Given the location of our experiment, our inference is per the landfill context for vultures engaged in nonflying activities. Importantly, vultures can engage in nonvolant activities, such as roosting and feeding, near airports. Manipulating the behavior of vultures in a nonlethal manner to reduce their local abundance, in flight or not, might reduce the probability of a collision with aircraft.

## Methods

### UAS trials

**Figure 1:**
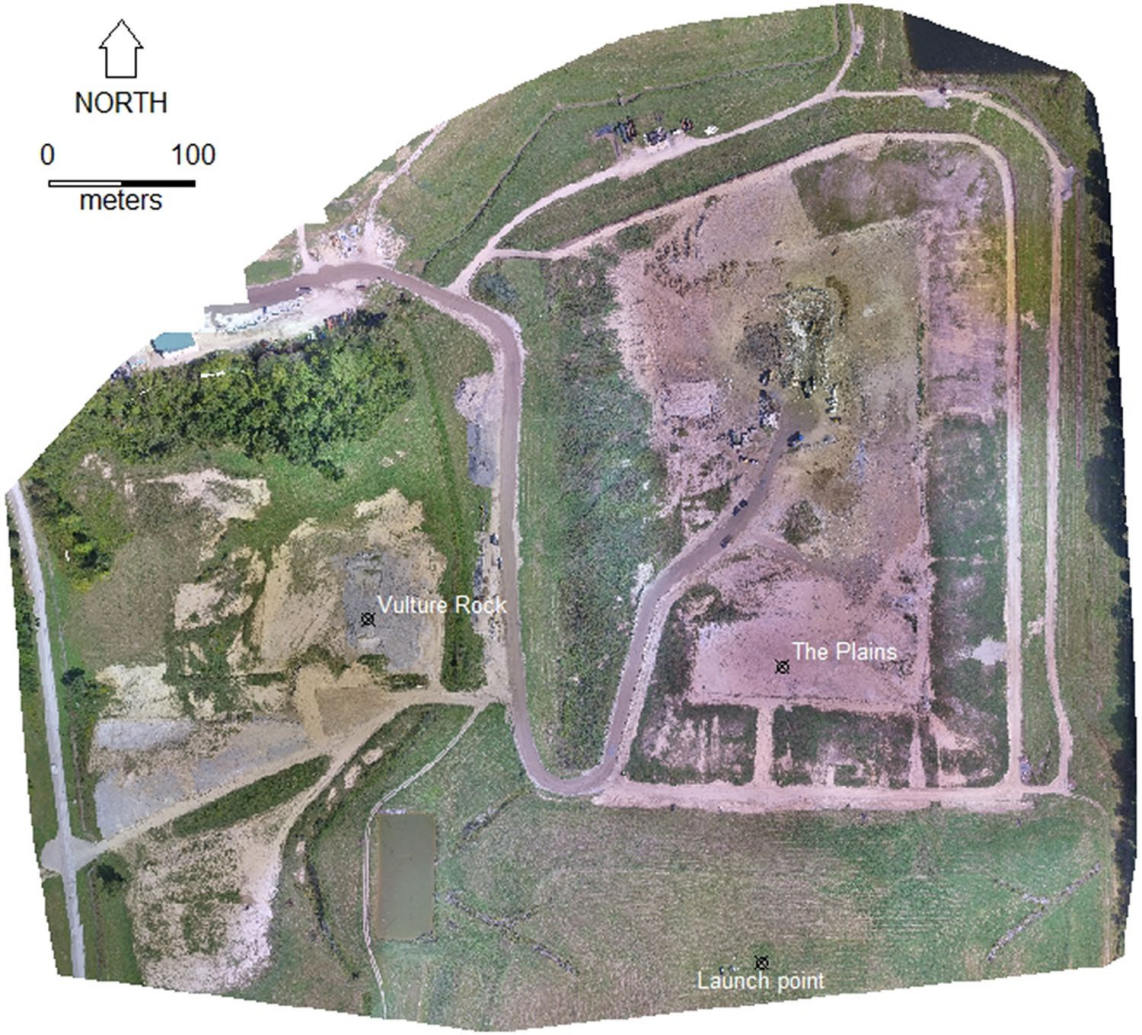
Study locations at the Erie County Landfill, Milan, Ohio. Base map was created using geo-referenced images from the DJI Inspire and Drone2Map (version 1.3.2.232, ESRI, Redlands, California, USA). Map was created by M.B.P.

**Figure 2:**
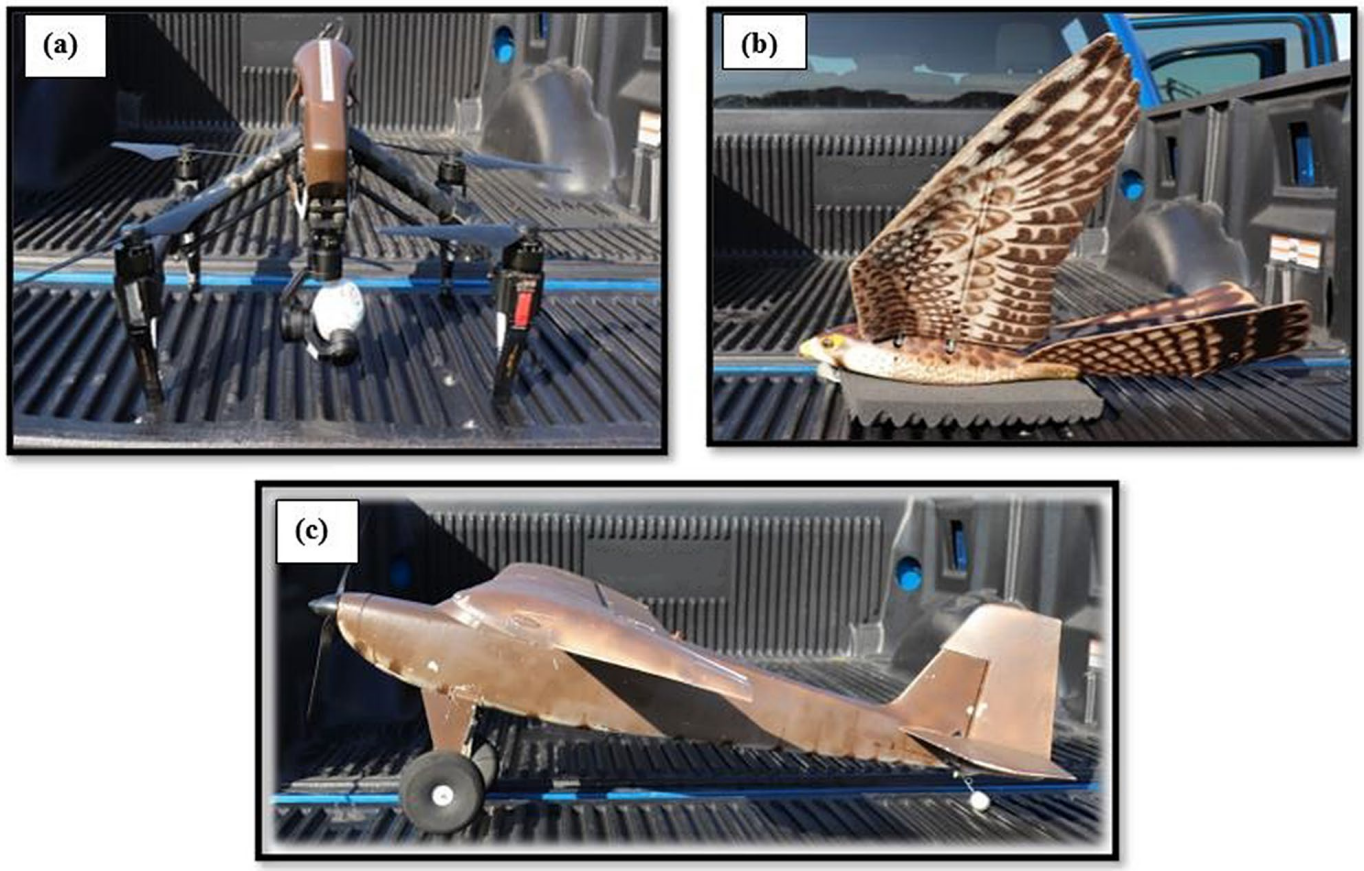
Photographs of the (**a**) multirotor ( DJI Inspire 1 V1.0 Da-Jiang Innovations Shenzhen, China), (**b**) ornithopter (Robird version 3, Clear Flight Solutions, Institutenweg, Netherlands), and (**c**) fixed-wing (E-flite Timber X 1.2 m BNF Basic with AS3X and SAFE Select, Hobby Zone, Eden Prairie, MN, USA). Images were taken by M.B.P.

We conducted the present study between 08 July 2019 and 03 September 2019 at the Erie County Landfill, Milan, Ohio (41.3434° N, − 82.5966° W, Fig. 1). The landfill is located 7 km south of Lake Erie and was frequented by groups of vultures in the summer^32^. Our treatments included ornithopter, fixed-wing, and multirotor UAS in an overhead or targeted pattern (see below). The ornithopter was the Robird (Fig. 2), which is an UAS modeled after a peregrine falcon, powered by flapping flight, and maneuvered using a mechanism in the tail (Clear Flight Solutions, Institutenweg, Netherlands). The multirotor (quadcopter) was a DJI Inspire 1 V1.0 (Da-Jiang Innovations Shenzhen, China). The fixed-wing platform was an E-flite Timber X 1.2 m BNF Basic with AS3X and SAFE Select with one propeller on the nose (Hobby Zone, Eden Prairie, MN, USA). The fixed-wing and multirotor were painted to match the countershading of the ornithopter. We present additional UAS specifics in the Supplementary File S1 according to a standardized reporting protocol^33^.

Overhead treatments involved a gridded ‘lawn-mower’ back and forth movement (usually West to East based on predominate local wind conditions) above the focal birds at ~ 30 m AGL^19^. Targeted treatments involved flying the UAS directly at focal turkey vultures (see below) that occasionally were surrounded by mixed flocks of herring and ring-billed gulls. For overhead UAS treatments, the UAS pilot-in-command (PIC) did not target an individual bird. In contrast, targeted UAS treatments involved selection of a focal individual by the PIC and observers. Focal vultures were selected in relation to their location in the grounded flock (e.g., perched higher). The PIC flew the UAS directly toward the focal bird but avoided collision.

Observers also scanned the area for bald eagles (*Haliaeetus leucocephalus*). Flights were not conducted if UAS operation would interfere with eagle movement or foraging in accordance with the Bald and Golden Eagle Protection Act. All treatment platforms were flown by a Part 107 UAS pilots from AERIUM Analytics (Calgary, Alberta, Canada). An additional UAS was launched prior to the treatment UAS to video record reactions (see below) and this UAS was flown by the first author (M.B.P. Part 107). All pilots completed the CITI Program Animal Care and Use Research Technicians Basic Course. Further, all operations occurred in Class G Airspace. Our methods were reviewed under the USDA National Wildlife Research Center (NWRC) QA-2963, amendment number: 01, and approved by the NWRC Institutional Animal Care and Use Committee. Our experiment was completed in accordance with the American Veterinary Medical Association guidelines. No animals were injured during this study.

We launched all UAS from a vegetated capped landfill area which was at least 170 m south of the focal birds at a similar elevation (Fig. 1). This minimum start distance helped control for potential biases associated with our presence and the launch of the UAS, as current recommendations suggest launching a UAS at least 50–100 m away from wildlife to avoid disturbance^6,29^. However, we recognize that the visual acuity of birds likely exceeds 170 m for most species^34–36^, which is similar to average soaring altitudes of turkey vultures while foraging^37^. There were instances (*n* = 20) when the vultures did not remain on the ground long enough to facilitate a UAS treatment. We were unable to determine the cause for every disturbance (e.g., ground vehicle disturbance), thus these flights were not used in our analyses.

Our observations at the Erie County Landfill indicated that vultures generally used two distinct, yet not independent locations, and traveled freely between sites. One area was the compressed landfill section (i.e., “The Plains”) and the other area was a group of mounds comprised of fill dirt and lime (i.e., “Vulture Rock”). Both sites were located away from the active dumping site and contained few food resources; instead, the area was mainly used for loafing. The research team positioned themselves at the launch point from 0830 h until 1430 h. We were restricted from staying later in the afternoon because of landfill operating hours.

We conducted a UAS treatment only if there was at least one turkey vulture on the ground at either location, as antipredator responses are measured more accurately when the bird is not flying. One observer with a spotting scope watched the focal vulture prior to launch of the treatment UAS. We attempted to follow a random treatment schedule (Supplementary File S2) to avoid replication within a day. We used a random number generator to produce numbers over two days to cover all possible treatments with a maximum of 3 treatments/day separated by at least 2 h at a given study location to minimize the changes of sampling the same individuals^38^. We then ranked the random numbers for each two-day period to generate a random order. Prior to the selected UAS launch, the observer collected count data on vultures and gulls at the study location, and then an “eye in the sky” UAS was launched. This UAS, a 3DR Solo (3D Robotics, Berkley, CA, USA) ascended to 119 m (390 ft), and then moved horizontally into a position directly over the focal vulture. The purpose of the 3DR Solo was for observation and to record bird reactions to the treatment UAS. We assumed that this altitude would pose little disturbance or perceived risk to the target vulture^6,18,39^, but we also noted any responses by target vultures to this UAS. If vultures left the ground prior to the 3DR Solo getting in position, we landed the UAS at the launch point.

The 3DR Solo carried a GoPro Hero 4 (GoPro, Inc, San Mateo, CA, USA) which video-recorded the entire flight in the linear field of view at 60 frames per second. The GoPro was set to a 1080-pixel resolution with low light and spot meter off, protune on, white balance on auto, ISO limit 400 (moderate light sensitivity), and high sharpness. After the 3DR Solo was in position over the target vulture, the treatment UAS was revealed from under an opaque fabric, launched, and flown in the predetermined approach scenario for at least 3 min. We were limited in the battery duration of the ornithopter. The fixed-wing and ornithopter were hand-launched into the prominent wind direction, whereas the multirotors were vertical-takeoff-and landing models. All UAS were flown manually during the trials. After the treatment UAS landed, the 3DR Solo returned to the launch point in the opposite flight pattern (horizontal return at the same altitude and above the PIC, then descent to a predetermined landing area). The observer recorded the focal bird reaction during the treatment, including reaction time since the reveal of the treatment UAS. The observer also noted any disturbance of the focal vulture prior to UAS approach. Once both UASs landed, the observer conducted post-treatment counts of gulls and vultures.

Ambient light conditions were measured after each UAS treatment by recording a 15-s average light intensity across wavelengths (µmol m^−2^ s^−1^) at approximately 1 m from ground level using a Li-Cor LI-250 Light Meter and LI-190SA Quantum Sensor (Li-Cor, Lincoln, NE, USA). In addition, we recorded wind speed (m s^−1^), wind direction, and temperature for each UAS treatment with a Kestrel 4500 Pocket Weather Tracker (Nielson-Kellerman, Boothwyn, PA, USA). We also collected UAS sound intensity from example treatments at the Erie County Landfill on 06 September 2019, after the completion of the experiment. We recorded sound using a hand-held HP-882A Digital Sound Level Meter LCD Noise Measuring Instrument (HoldPeak, Guangdong, China), but this device did not store data. We recorded the Digital Sound Level Meter’s LCD screen with an iPad mini (5th generation, Apple Inc. Cupertino, CA, USA). We used a general linear model to assess covariance among fixed effects (UAS platform and UAS approach) and sound intensity (Supplementary File S3).

### Perceptual modeling

Birds might detect UAS approach based on brightness or intensity (achromatic) or hue (chromatic) relative to the background ambient light^40^. Differences in the visual background can affect how visually salient the UAS is in a given visual scene, from the turkey vulture’s visual system perspective, by changing the achromatic and chromatic contrast of the UAS relative to the background. To account for this, we modeled each UAS against the two main visual background that the vultures would encounter during a UAS approach: the sky and the horizon. These two backgrounds represent the vulture’s perspective of the UAS in an overhead and targeted UAS approach, respectively. We note that based on the position of our launch point, the approaching UAS was backlit by the sun, which could increase the disability glare. Specifically, the excess of sunlight in the eye chamber can reduce object contrast and resolution^40,41^.

To estimate perception of the UAS against the environmental background, we used the receptor-noise limited visual model^42^ adjusted for avian species sensitive to violet wavelengths. Genomic sequencing has identified the turkey vulture in the violet sensitive color vision class^43^. We note, though, that many gull species are ultra-violet sensitive^44^. The perceptual model included reflectance from the UAS and ambient radiance (Supplementary File S4), and we assumed a generic violet-sensitive visual system, as the visual configuration of the turkey vulture has not been quantified^43^. We used the Pavo package^45^ and expressed the units of chromatic and achromatic contrasts as units of just noticeable differences (JND). Values of JND < 4 are difficult for an animal to distinguish the UAS from the visual background^44^.

### Post-processing of the UAS video

Because the 3DR Solo “eye in the sky” UAS was not configured to collect Motion Imagery Standards Board (MISB) compliant video (e.g., paired with associated spatial metadata), we exported still frames individually for geo-referencing to estimate vulture FID from the approaching UAS and UAS approach speed (Supplementary File S5).

### Power analyses

We attempted to calculate possible effect sizes for our planned study, rather than use arbitrary levels of effect sizes (e.g., small, medium, and large) to determine necessary sample sizes^46,47^. We estimated from a 2018 pilot study that a sample size of 96 UAS trials would allow us to detect a significant effect size of 0.42 for the interaction of UAS platform and approach on the vulture remaining index (number of vultures in the study area after a treatment/number of vultures in the study area before a treatment). We estimated from a range of scenarios (i.e., first vulture to react, average reaction time, and a random vulture to react) that between 66–174 UAS trials would allow us to detect a significant effect size of 0.30–0.51 for the interaction of UAS platform and approach on vulture reaction times. For vulture FID, we estimated that 36 UAS trials was needed to detect a significant effect size 0.63 for UAS approach and 464–640 UAS trials to detect a significant effect size of 0.14–0.17 for UAS platform (Supplementary File S6).

### Statistical analyses

It was not possible to mark individual vultures for this study. Although we waited an arbitrary threshold of 2 h between UAS treatments for each study location to increase the chances of exposing different individuals, individual vultures might have been exposed to multiple treatments based on vulture landfill use^32^. Using data collected from the same individuals without correction would, in most modeling approaches, violate the independence of data assumption^48^. Therefore, we built models with and without a temporal autocorrelation structure for each continuous response variable and plotted the residuals^49^. If behavioral responses were correlated with time, we would expect their residuals to be correlated with the time lag between observations^49^. We used Akaike’s Information Criterion to rank and select the constructed general linear models with the lowest AIC^50^. We did not observe temporal autocorrelation with any of our response variables, as the model residuals did not surpass the generated thresholds for correlation (Supplementary File S7). Therefore, we used general or generalized linear models (instead of mixed models) to analyze our data.

The general linear models constructed examined the effects of UAS platform, approach type, and their interaction on different dependent variables: focal vulture reaction time since UAS reveal, focal vulture FID, the vulture remaining index, and latency to return. The generalized linear model constructed examined the effects our fixed effects on the probability of a vulture reaction. We considered UAS speed, starting distance, ambient light intensity (as a proxy of potential glare effects), wind speed, group size of mixed species of gulls (both ring-billed and herring) on the ground, and group size of turkey vultures on the ground as covariates. Wind speed is important for local movement decisions of turkey vultures, including when to depart an overnight roost^51^. We included group size of turkey vultures because an increase in group size of conspecifics likely increases the dilution effect (i.e., potentially affecting escape response and FID) by way of an abundance of potential targets^52^. Furthermore, birds use social information of their conspecifics to weigh escape decisions from vehicles^53,54^. Turkey vultures and gulls both used the landfill and could be considered a mixed-species aggregation^55^, foraging in close proximity could influence escape responses to the UAS approach; thus we included group size of gulls. We examined the normality of model residuals and the homogeneity of variances with untransformed and transformed variables. Then, we selected the transformations that helped meet model assumptions.

As a post hoc analysis (i.e., we did not have preliminary data to run a power analysis), we examined UAS platform as a fixed effect on the number of passes a UAS performed for targeted approaches at turkey vultures on the ground. We determined a pass as a direct trajectory close (< 5 m) to a vulture on the ground from reviewing the “eye in the sky” 3DR Solo videos. If vultures were flushed prior to the end of the treatment, the treatment UAS pilot defended the area by flying the UAS low over the study area preventing vultures from landing. Those movements were not considered passes because no vultures remained.

We ran our models in R^56,57^ (4.0.2, www.r-project.org). General linear models used type III sums of squares. We present least squared means ± standard error (SE), unless stated otherwise. We also present means ± standard error (SE) for untransformed raw data when appropriate. R code is presented in Supplementary File S8.

## Results

### Auditory and visual perception of UAS platforms

**Figure 3:**
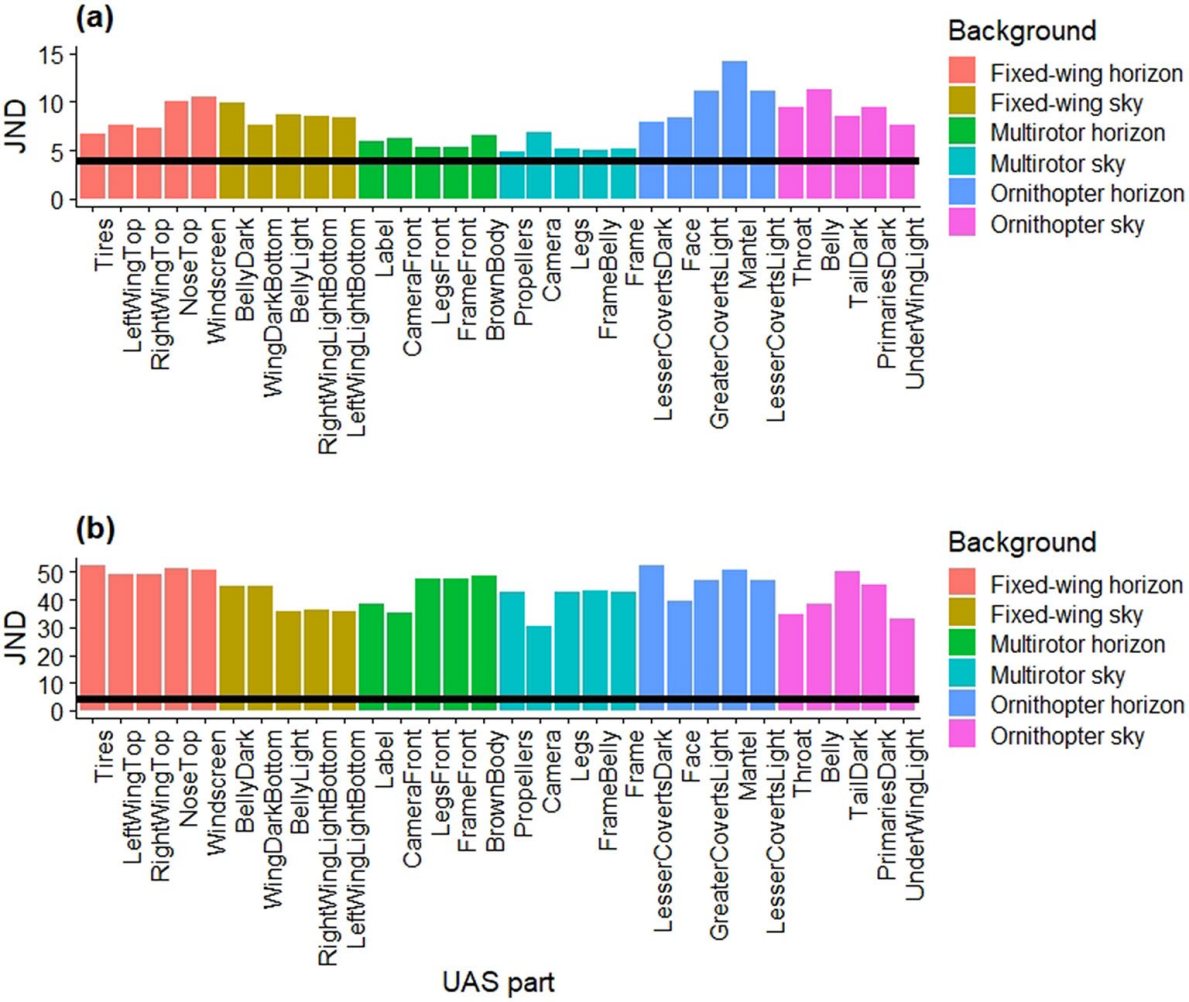
Chromatic (**a**) and achromatic (**b**) contrast values for five of the largest parts of three UAS platforms under party cloudy ambient light conditions, using visual system properties of general violet sensitive avian species for two different backgrounds (sky and horizon). Just noticeable differences (JND, black horizontal bar) > 4 indicates the object can be distinguished from the visual background. The fixed-wing and multirotor were painted to match the countershading of the ornithopter.

We did not measure sound intensity for every trial, instead we flew example trials over microphones following the completion of all trials. Sound intensity did not vary across UAS platforms (*F*_(2,2)_ = 11.40 , *P* = 0.08) nor approach type (*F*_(1,2)_ = 0.49, *P* = 0.56) in our example trials. From the perspective of a general violet sensitive avian species, such as the turkey vulture^43^, we estimated the visual contrast in the chromatic and achromatic dimensions^44^ of different components of the three UAS platforms under the sky and horizon backgrounds (Fig. 3). The sky background is representative of the vulture’s perspective of the UAS in an overhead approach, whereas the horizon background is representative of a targeted UAS approach. We found that all values of visual contrast were above the discrimination threshold of 4 JND, suggesting that animals would have been able to detect all UAS parts against the two visual backgrounds (Fig. 3). We then used the values of each of the 5 largest components per UAS to estimate averages in each visual background and found that UAS platforms differed significantly in their chromatic contrasts (*F*_(2,12)_ = 20.3, *P* < 0.01). Specifically, when viewed in an overhead approach (i.e., background was the sky), the multirotor (5.42 ± 0.46 JND, *n* = 5) had significantly lower levels of chromatic contrasts than the fixed-wing (8.64 ± 0.46 JND, *n* = 5, *t*_12_ = 5.00, *P* < 0.01) and ornithopter (9.24 ± 0.46 JND, *n* = 5, *t*_12_ = − 3.82, *P* < 0.01). Further, when viewed in a targeted approach (i.e., background was the horizon), the UAS platforms differed in their chromatic contrasts (*F*_(2,12)_ = 8.54, df = 2 and 12, *P* < 0.01). Specifically, the multirotor (5.86 ± 0.80 JND, *n* = 5) had lower chromatic contrasts than the ornithopter (10.54 ± 0.80 JND, *n* = 5, *t*_12_ − 4.13, *P* < 0.01). However, we did not find significant differences in the averaged achromatic contrast among UAS platforms for the sky (*F*_(2,12)_ = 0.04, *P* = 0.96) or horizon background (*F*_(2,12)_ = 2.90, *P* = 0.09). Overall, the multirotor was the least visually salient^8^ and the ornithopter was the most visually salient to violet sensitive bird species.

### Responses to UAS approach

Between 08 July 2019 and 03 September 2019, we obtained 100 UAS trials. However, in 6 trials we were unable to determine if the focal vulture reacted to the approach of the UAS or an outside disturbance; these 6 trials were removed from analyses associated with the focal vulture. The focal turkey vulture reacted by escaping from the UAS in 59 out of 94 trials (~ 63%). UAS speed (*F*_(2,82)_ = 4.30, *P* = 0.02) and starting distance (i.e., distance between the launch point and the focal vulture, *F*_(2,88)_ = 4.54, *P* = 0.01) varied with UAS platform, but not approach type nor the interaction term (Supplementary File S9). Despite attempts to standardize UAS speed across treatments, fixed-wing approaches were ~ 1.3 times faster (17.0 ± 0.86 m s^−1^, *n* = 35) than ornithopter approaches (13.3 ± 0.97 m s^−1^, *n* = 28, *t*_82_ = 2.82, *P* = 0.02). Approach speed of the multirotor (14.5 ± 0.92 m s^−1^, *n* = 31) did not differ compared to speed of the fixed-wing or ornithopter. Also, because of enhanced controller range and maneuverability (i.e., the pilot was about to maneuver the multirotor to greater distances), starting distances were ~ 1.2 times greater with multirotor treatments (293 ± 10.27 m, *n* = 31) than fixed-wing treatments (254 ± 9.66 m, *n* = 35, *t*_88_ = − 2.78, *P* = 0.02) and ornithopter treatments (257 ± 10.80 m, *n* = 28, *t*_88_ = 2.41, *P* = 0.05). Even though the starting distances of the fixed-wing and ornithopter were shorter than the multirotor, they were well beyond (> 25 m) the FID shown by vultures in this experiment. Because starting distance varied with UAS platform, we did not include it in our models to avoid issues of variation between independent factors. UAS speed was included in the vulture reaction time and latency to return models, as there was no association with UAS platform and approach type (Supplementary File S9).

**Figure 4:**
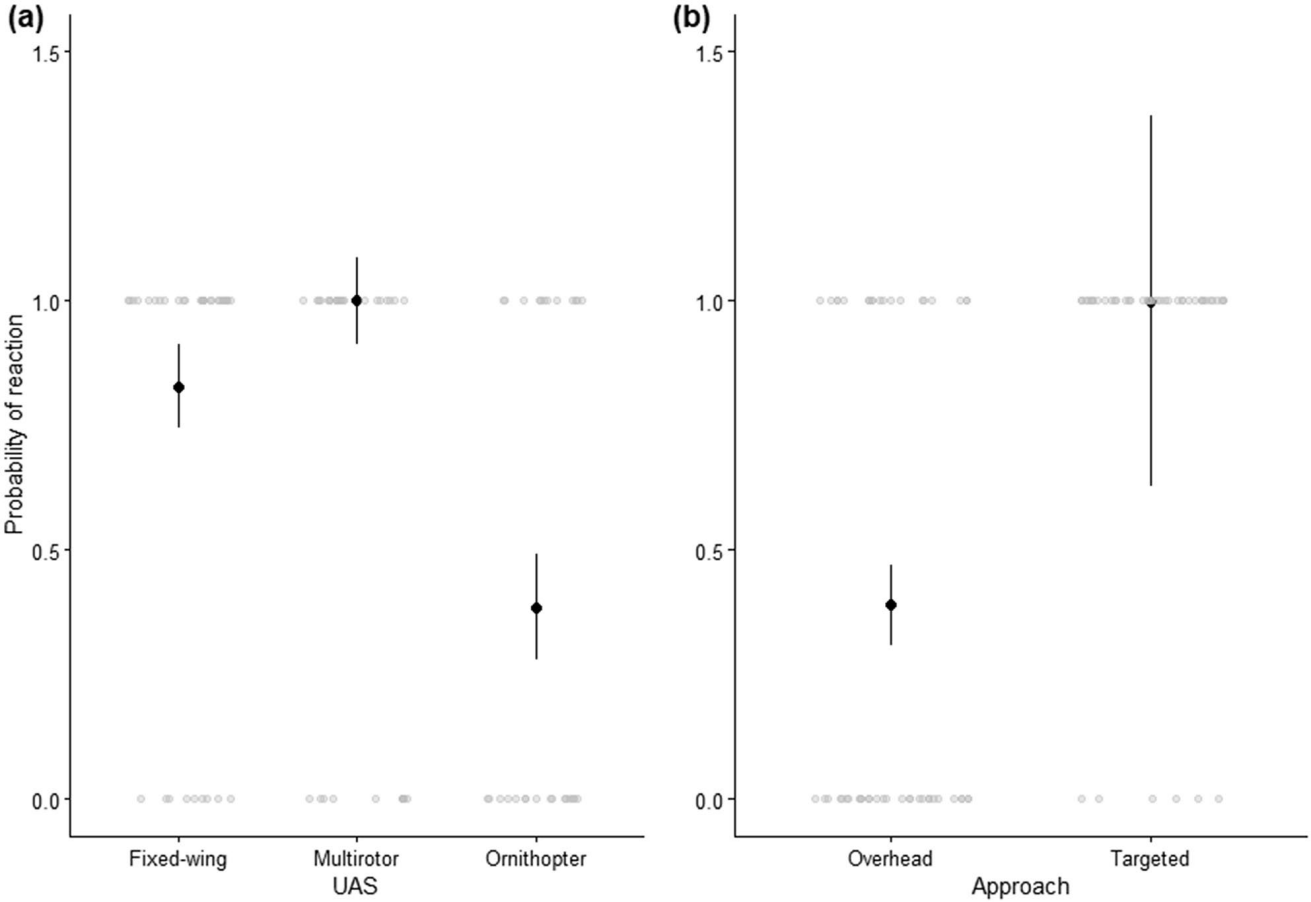
Probability of focal turkey vulture reaction in response to (**a**) UAS platform and (**b**) approach. Raw data are shown as points. UAS trials were conducted at the Erie County Landfill, Ohio, between July and September 2019.

The probability of vulture reaction was influenced by UAS platform and approach type (Table 1). The probability of a vulture reacting was ~ 2.2 times greater in response to the fixed-wing (0.83 ± 0.08) than the ornithopter (0.38 ± 0.11, *Z* = − 2.73, *P* = 0.02, Fig. 4). However, there was no difference between ornithopter and multirotor treatments (1.00 ± 0.09, *Z* = − 0.01, *P* = 0.99) or multirotor and fixed-wing treatments (*Z* = 0.01, *P* = 1.00). As predicted, there was greater probability of reaction for targeted rather than overhead approaches (Table 1, Fig. 4). Specifically, the probability of a vulture reacting was ~ 2.6 times greater in response to a targeted (0.99 ± 0.37) than an overhead approach (0.39 ± 0.08).

**Table 1:** Results from the generalized linear model analysis using a binomial distribution of turkey vulture reactions in response to UAS approach.

Binomial focal vulture reaction (*n* = 94)	X^2^	df	*P*
UAS platform	11.7	2	** < 0.01**
Approach	23.6	1	** < 0.01**
UAS platform * approach	3.26	2	0.20
Ambient light (µmol m^−2^ s^−1^)	0.85	1	0.36
Vulture group size	2.23	1	0.14
Gull group size	1.63	1	0.20
Wind speed (m s^−1^)	1.37	2	0.20

For UAS platform, the categories were fixed-wing, multirotor, and ornithopter and approach included targeted or overhead. Trials were conducted at the Erie County Landfill, Ohio, between July and September 2019. Significant results are marked in bold.

**Figure 5:**
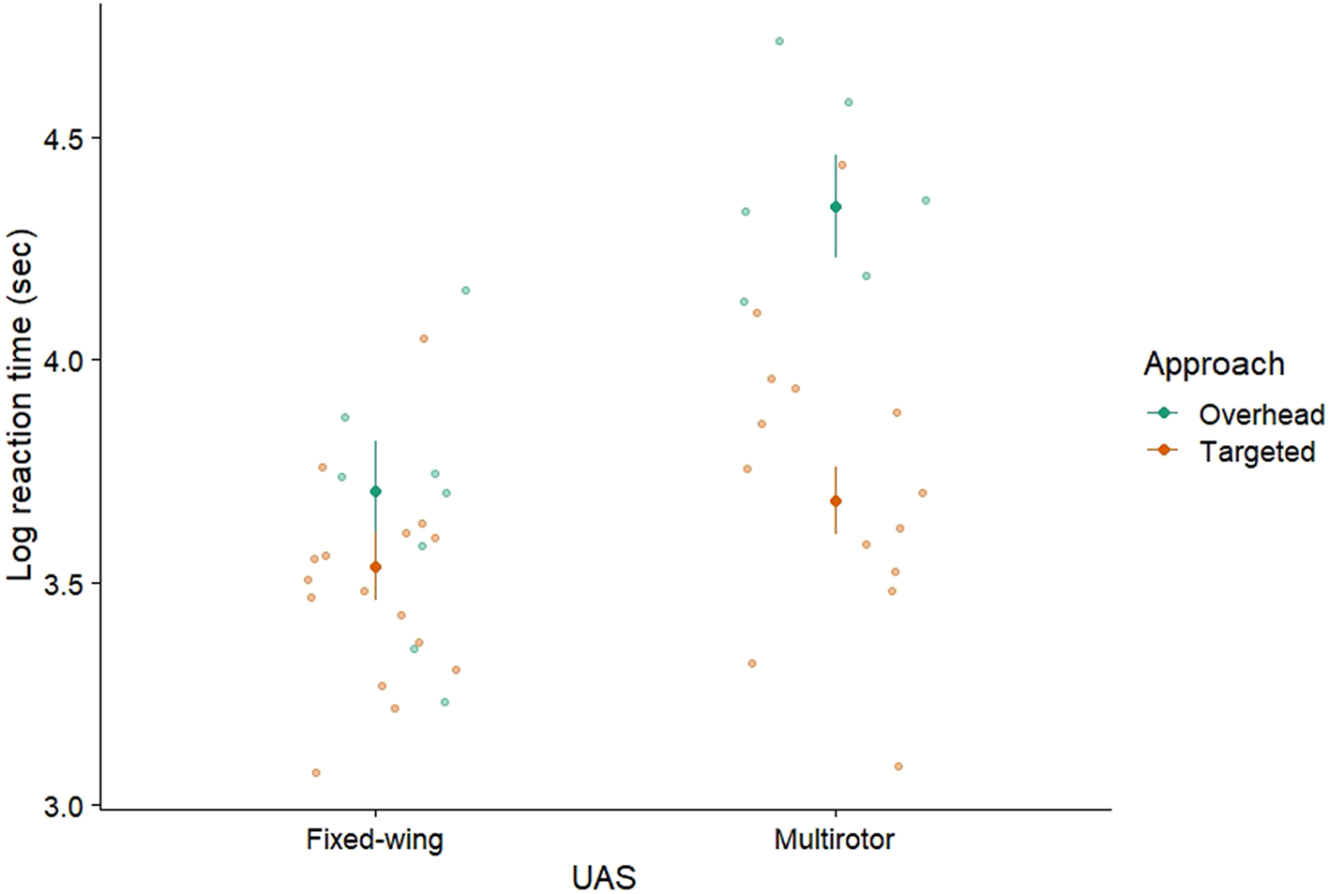
Interaction between the fixed effects of UAS platform and approach on vulture reaction time since reveal. Raw data are shown as points. Ornithopter treatments were removed because of low sample size.

Focal vulture reaction time was recorded in 59 trials, however; we only observed reactions to overhead ornithopter treatments for 3 trials. Therefore, we removed ornithopter treatments (*n* = 12) to obtain a total of 47 UAS trials for the vulture reaction time model. Focal vulture reaction time ranged from 21.58–111.89 s with an average of 44.76 ± 2.85 s. Focal vulture reaction time (log_10_ transformed) was affected by UAS platform, approach, and their interaction (Table 2). Across approaches, focal turkey vultures reacted ~ 1.1 times sooner to fixed-wing approaches (3.62 ± 0.07 s, *n* = 26) than multirotor approaches (4.01 ± 0.07 s, *n* = 21). Focal turkey vultures reacted ~ 1.1 times sooner to targeted approaches (3.61 ± 0.05 s, *n* = 31) than to overhead approaches (4.02 ± 0.08 s, *n* = 16). However, these single effects should be taken with care given the significant interaction effect, whereby focal turkey vultures reacted ~ 1.2 times earlier to targeted than to overhead approaches with the multirotor (*t*_36_ = 4.84, *P* < 0.01, Fig. 5), but the difference between approach types was not significant with the fixed-wing (*t*_36_ = 1.20, *P* = 0.24; Fig. 5).

**Table 2:** Results from general linear models type III sums of squares analyses of turkey vulture responses to UAS approach.

	*F*	df	*P*
**Focal vulture reaction time (*n* = 47)**			
UAS platform^1^	14.98	1, 36	** < 0.01**
Approach	17.08	1, 36	** < 0.01**
UAS platform * approach	6.55	1, 36	**0.01**
Square root gull group size	2.11	1, 36	0.15
Wind speed (m s^−1^)	1.44	1, 36	0.24
Square root UAS speed	0.05	1, 36	0.83
Ambient light (µmol m^−2^ s^−1^)	0.04	1, 36	0.84
Log_10_ vulture group size	0.00	1, 36	0.97
**Square root focal vulture FID (*n* = 35)**			
UAS platform^2^	0.01	2, 28	0.99
Square root gull group size	3.77	1, 28	0.06
Log_10_ vulture group size	1.98	1, 28	0.17
Wind speed (m s^−1^)	1.37	1, 28	0.25
Ambient light (µmol m^−2^ s^−1^)	0.00	1, 28	1.00
**Vulture remaining index (*n* = 100)**			
UAS platform^2^	4.69	2, 91	**0.01**
Approach	18.49	1, 91	** < 0.01**
UAS platform * approach	1.10	2, 91	0.34
Square root gull group size	1.34	1, 91	0.25
Ambient light (µmol m^−2^ s^−1^)	0.76	1, 91	0.38
Wind speed (m s^−1^)	0.16	1, 91	0.69
**Log**_**10**_** latency to return (*n* = 31)**			
UAS platform^2^	0.03	2, 23	0.98
Square root UAS speed	5.07	1, 23	**0.03**
Log_10_ vulture group size	0.77	1, 23	0.39
Wind speed (m s^−1^)	0.72	1, 23	0.40
Square root gull group size	0.11	1, 23	0.75
Square root ambient light (µmol m^−2^ s^−1^)	0.00	1, 23	0.98

For UAS platform, the categories were fixed-wing, multirotor, and ornithopter and approach included targeted or overhead. Trials were conducted at the Erie County Landfill, Ohio, between July and September 2019. Significant results are marked in bold.

^1^Multirotor and fixed-wing.

^2^Multirotor, fixed-wing and ornithopter.

Focal vulture FID was calculated from the isolated georeferenced photographs obtained from the “eye in the sky” UAS. This UAS was launched prior to the reveal of the treatment UAS and its presence did not influence bird reactions (e.g., flight height was over 115 m and was launched over 100 m away and M.B.P. pers. comm.) The Root Mean Square (RMS) error was less than 0.001 m for 401 georeferenced photographs compared to the base orthomosaic map, which relates to a < 0.001 m degree of spatial accuracy (Supplementary File S10). We obtained 45 trials for FID, however, we had low sample sizes (≤ 4) for overhead treatments. Therefore, we removed overhead treatments for FID (*n* = 10), for a total of 35 trials and were unable to include UAS approach as an independent effect. FID ranged from 10.18–144.77 m and mean FID was 60.28 ± 5.44 m (*n* = 35). Despite our predictions, we did not observe an effect of UAS platform on focal vulture FID (Table 2). There were no differences in FID between fixed-wing (7.42 ± 0.59 m, *n* = 13) and multirotor (7.54 ± 0.60 m, *n* = 13) or ornithopter (7.46 ± 0.70 m, *n* = 9) approaches (Table 2).

The vulture remaining index (number of vultures in the study area after a treatment/number of vultures in the study area before a treatment) ranged from 0–2 vultures. A vulture remaining index of 2 indicated that the number of vultures in the study area doubled (i.e., not all vultures were dispersed, and more were present after the UAS flight). Mean vulture remaining index was 0.48 ± 0.05 vultures. Because UAS speed and starting distance varied significantly with UAS platform (Supplementary File S2), they were not included for this dependent variable. We found that UAS platform and approach type affected the vulture remaining index but did not find an interaction effect (Table 2). The vulture remaining index after multirotor UAS treatments (0.29 ± 0.09 vultures, *n* = 32) was ~ 2.3 times smaller than after ornithopter UAS treatments (0.66 ± 0.09 vultures, *n* = 32, *t*_91_ = − 3.06, *P* < 0.01). There were no differences in the vulture remaining index between fixed-wing (0.48 ± 0.08 vultures, *n* = 36) and multirotor approaches (*t*_91_ = 1.57, *P* = 0.26), and fixed-wing and ornithopter approaches (*t*_91_ = − 1.57, *P* = 0.26). The vulture remaining index after targeted approaches (0.27 ± 0.07 vultures, *n* = 49) was ~ 2.6 times smaller than after overhead approaches (0.69 ± 0.07 vultures, *n* = 51).

**Figure 6:**
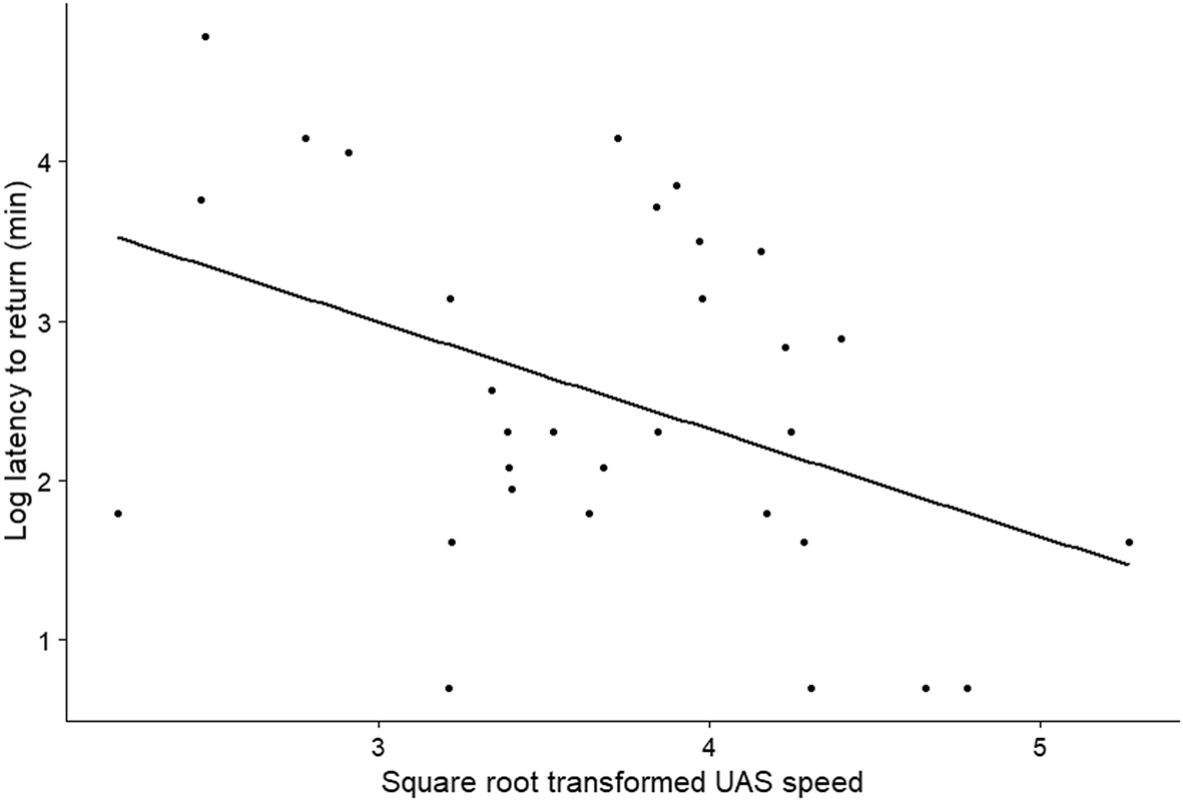
Relationship between vulture latency to return to the study area on the ground and UAS approach speed.

All vultures on the ground were flushed from the study area in 44 UAS trials (~ 46%). Latency to return ranged from 2–120 min and mean latency time was 22.48 ± 3.93 min. We also had to remove overhead treatments because of low sample size for this model. We observed no significant effect of UAS platform on latency for a vulture to return (Table 2): fixed-wing (2.48 ± 0.35 min, *n* = 12), multirotor (2.59 ± 0.35 min, *n* = 13), and ornithopter approaches (2.48 ± 0.49 min, *n* = 6). We did find an effect of UAS speed on latency; an UAS that approached slower resulted in a longer latency period (Table 2, Fig. 6).

### Post hoc analysis of number of UAS passes

For application in the airport environment, UAS operations may be limited for safety reasons. Therefore, we examined the number of targeted passes to clear an area of vultures to gauge the management effort. Number of targeted UAS passes needed to disperse all vultures from the ground differed by UAS platform (*F*_(2,43)_ = 4.12, *P* = 0.02). The number of multirotor approaches needed to clear an area (1.13 ± 0.31 passes, *n* = 15) was roughly half that of ornithopter approaches (2.29 ± 0.32 passes, *n* = 14, *t*_43_ = − 2.62, *P* = 0.03). There were no differences in the number of passes needed to clear the study area between fixed-wing (2.12 ± 0.29 passes, *n* = 17) and multirotor approaches (*t*_43_ = 2.35, *P* = 0.06) and fixed-wing and ornithopter approaches (*t*_43_ = − 0.39, *P* = 0.92).

## Discussion

Contrary to our predictions regarding form of UAS platform, direction of UAS approach and their interaction, there were no consistent behavioral responses of free-ranging turkey vultures at a landfill to our treatments. Specifically, we found no effect of UAS platform on latency to return or FID. However, we likely would not have realized an adequate sample size to discern an effect by simultaneously minimizing Type I and Type II errors of platform on FID (based on our a priori power analyses) and we could not conduct a power analysis for latency to return because of lack of previous research. Turkey vulture perceived risk, as indicated by reaction time, was only enhanced by flying in a targeted vs. overhead approach with the multirotor platform. For vulture reaction and the vulture remaining index, we found similar effects of UAS platform and approach, but no interaction effect. The fixed-wing generated faster reactions, but the multirotor needed fewer passes to clear an area and the vulture remaining index was lower after multirotor presentations. Vulture responses to the ornithopter indicated that these vultures perceived this platform as the least risky, despite its aerial predator-like appearance and high perceived risk by passerines and waterfowl^8,18^. Alternatively, if the ornithopter was perceived as an aerial predator the lack of responses could be explained by previous experience with aerial predators and their habits (e.g., bald eagles hazing vultures in the winter for their crop contents^28^).

Confounding variables of UAS speed and starting distance could possibly explain our results. However, we discuss below, why these are minor limitations. Although we attempted to control for approach speed of UAS by visually adjusting flying patterns, local wind conditions and UAS capabilities caused differences in approach speeds between treatments. Therefore, we were only able to include speed as a covariate in some of our models. When included, UAS approach speed was not significant in its effect on vulture reaction time but was the only significant measured covariate on latency to return. Variations in UAS speed between platforms was 4 m/s which, although, statistically significant this difference probably did not result in the vultures assessing the faster UAS as risker. Specifically, previous research showed that turkey vultures largely did not adjust their spatial escape responses based on speed^34^. There is little evidence of bird response to vehicle approach that suggests a temporal margin of safety (i.e., whereby the bird adjusts escape according to approach speed): most evidence indicates a spatial margin of safety^58,59^.

However, speed of the approaching UAS was negatively correlated with latency to return; therefore, a slower UAS approach was perceived as riskier^60^. We note that previous research on latency to return relates to animals hiding in refuge^61,62^; in our study, dispersed vultures became airborne, which does not conceal the vulture from aerial threats. Still, because aerial threats against a species this large are few, taking flight allows greater visibility of the area and the UAS itself.

Additionally, we were unable to standardize starting distance, which is also important for avian escape decisions from UAS^31^. Starting distance for all flights was beyond (> 25 m) the FIDs recorded in this experiment^34^. However, vultures have the visual acuity to detect the UAS from > 100 m and we cannot rule out that they had detected its presence. We were unable to measure alert distance because of the difficulty in observing those behaviors from > 100 m^63^.

Further, we attempted to control for UAS size by choosing platforms of similar wingspans. Still, UAS platform-specific components (e.g., large body of the fixed-wing vs. skeletal design of the multirotor) or flight patterns (e.g., louder propeller noise when multirotors perform erratic maneuverers) likely influenced the visual looming rate and proximate audible differences among platforms^64,65^. However, size of the looming vehicle, particularly relative to natural threats, might not be as important in the decision-making process^58^.

Based on our power analyses, we did not obtain the necessary sample size to discern an effect of UAS platform on turkey vulture FID. We were unable to include approach type in our FID models because of too few samples from overhead treatments. However, UAS approach type and associated conditions might not be as risky in a 3-dimensional approach compared to the more restricted approach types available to terrestrial vehicles^53,66^. For example, across 22 terrestrial and aquatic bird species, FID did not differ by UAS approach altitude (4 m or 10 m)^31^. Further, Collins et al.^67^ did not observe differences in FID for great egrets (*Ardea alba*) based on UAS approach altitude. Even approach direction in association with occupied aircraft did not influence FID of multiple bird species^54^. Also, perhaps the targeted approach angle we employed (< 10°), much shallower than the typical (~ 90°) aerial predator attack angle^6,68^, was perceived as less risky as measured by vulture FID^69^.

The only platform that enhanced turkey vulture responses, as measured by reaction time, was the multirotor in a targeted versus overhead approach. Given that this platform was the least salient relative to the estimated visual capabilities of the turkey vulture, targeted versus overhead approaches likely enhanced vulture detection of the UAS. Additionally, although the flight performances of the ornithopter and fixed-wing were greatly influenced by wind speed and direction, the internal stabilization technology of the multirotor and smaller surface area enabled a steady, unwavering trajectory irrespective of wind conditions. Variation in flight patterns caused by the wind might have increased the detection of the ornithopter and fixed-wing and allowed the multirotor a relatively stealthier slower approach (see above). Again, escape responses to multirotor approach differed between approach type, suggesting enhanced detection of the UAS during targeted approaches. The precise maneuverability of the multirotor also enabled this platform to spend more time over the study area in both approach types, increasing the exposure to the stimulus, whereas the other platforms had to perform wide turning maneuvers. This effect of greater time over the site could explain why the multirotor was able to disperse more vultures with fewer passes. Moreover, if we consider potential effects of form (e.g., the fixed wing), speed, maneuverability, and possibly size of the UAS relative to the target species, questions remain as to how combinations of effects might better serve in enhancing disturbance during hazing and minimizing disturbance during wildlife survey applications.

In summary, targeted UAS approaches should be used to disperse nonflying turkey vultures with either a multirotor or fixed-wing based on management goals and logistics. We acknowledge that the vulture latency periods recorded in our landfill context are relatively short (e.g., 2–120 min) and thus might not reduce the probability of an aircraft collision. It is unknown if vultures would remain away from the targeted area if these methods were applied at an airport or if vultures were pursued after taking flight, with the intention of moving birds away from the air traffic pattern. We note that UAS hazing could cause stress^70^, but these disturbances should be weighed against the consequences of an aircraft-vulture collision. Vultures’ ingestion of plastic at landfills also represents a conservation concern^71^ which could have a greater negative consequence on fitness and ecosystem health than direct UAS disturbance.

We note that there are many logistical considerations for operating UAS in the airport environment (e.g., limited battery duration, lack of access to select areas, long set up times for some UAS platforms). Further considerations include space requirement for UAS to operate. For example, fixed-wing flight operations might be limited because of the unobstructed open-space requirements^12^ in addition to the requirement of large horizontal take-off and landing areas. Even so, this platform could elicit a vulture reaction 1.5 times faster than a multirotor, which might allow the vulture to escape the trajectory of the aircraft, thus avoiding a collision^34^. The practicality of using this tool in an airport context to reduce strikes will likely be dependent on the local abundance of vultures, spatial and temporal characteristics of aircraft flight routes and vultures, and ability to conduct UAS operations in areas with anticipated vulture presence. We suggest that the use of UAS for vulture management be integrated with other nonlethal techniques^72^, including removal of attractants (e.g., animal carcasses) which aim at reducing the local abundance of vultures. Employing UAS hazing with other wildlife management techniques would reduce the probability of vultures habituating to UAS approach^59^. Other techniques to reduce strike risk include using falconry birds to disperse wild birds out of the path of aircraft and displacement distances of municipal solid waste landfills, however, the utility of these deterrents to reduce strike risk with vultures is unknown and are not feasible for many airports^73,74^.

Given the ability of the multirotor to disperse vultures, its vertical take-off and landing ability, and ability to assess remote areas, future research should focus on enhancing the perceived risk of multirotor platforms to species of interest through flight patterns (e.g. 90 degree angle approach type) and additions (e.g., onboard lighting) tuned to the visual capabilities of the species which would aid in reducing vulture habitation to UAS approach^75,76^. Future research should also consider combinations of effects (e.g., UAS size, form, speed, and maneuverability) relative to the target species size, antipredator ecology, and avian flight escape behaviors in the context of mitigating aircraft strike risk. Understanding vulture escape behaviors (e.g., direction of escape and latency to return) in response to UAS dispersal will guide UAS applications to reduce strike frequency with aircraft in the airport environment.

## Supplementary Information

Supplementary Information.

## Data Availability

Data are available from USDA Forest Service digital repository: 10.2737/NWRC-RDS-2021-003.
