# Supplementary material for: Responses of turkey vultures to unmanned aircraft systems vary by platform
Source: Sci Rep. 2021 Nov 4;11:21655. doi: 10.1038/s41598-021-01098-5 (PMC8569017; doi:10.1038/s41598-021-01098-5)
Supplement: Supplementary file 1 — Supplementary Information. [file 41598_2021_1098_MOESM1_ESM.docx]

Responses of turkey vultures to unmanned aircraft systems vary by platform

Morgan B. Pfeiffer, Bradley F. Blackwell, Thomas W. Seamans, Bruce N. Buckingham, Joshua L. Hoblet, Patrice E. Baumhardt, Travis L. DeVault, Esteban Fernández-Juricic

# Supplementary Files

**Supplementary File S1**. UAS specifications

**Supplementary File S2.** Random UAS treatment schedule for UAS flights at the Erie County Landfill

**Supplementary File S3.** Sound intensity of UAS example trials

**Supplementary File S4**: Post- processing of percent reflectance data from the three UAS

**Supplementary File S5**: Georeferencing methodology

**Supplementary File S6.** Pilot study protocol and power analyses

**Supplementary File S7**: Temporal autocorrelation results

**Supplementary File S8.** R Code for statistical analyses

**Supplementary File S9.** Covariate model summaries

**Supplementary File S10.** Metadata for georeferenced photographs

# **Supplementary File S1**. UAS specifications

The ornithopter was a Robird and was flown using a Jeti DS-14 transmitter. The multirotor was a DJI Inspire 1 V1.0 and was flown using a proprietary GL658A transmitter. The fixed-wing was an E-flite Timber X 1.2m BNF Basic flown using a Spektrum DK63 transmitter. Battery durations and maximum windspeed limitations differed per platform: ~20 minutes for the multirotor at 10 m s-1 windspeed, ~10 minutes for the fixed-wing at 10 m s-1 windspeed, and ~4 minutes for the ornithopter at 9 m s-1 windspeed. To achieve a similar appearance, the fixed-wing and multirotor were painted using Folk Art Paints in 4646CA Vintage White and 4654CA Real Brown (Plaid Enterprises Inc., Norcross, GA, USA) to match the countershading of the ornithopter. The wingspan of the fixed-wing platform (1.18 m) was within 6% of the ornithopter (1.11 m) and the wingspan of the multirotor (0.74 m) was 33% less than that of the ornithopter. The fixed-wing model experienced damage during the study and was replaced with an identical model (painted as described, above) for three flights from 30 August to 03 September 2019. We recognized, however, that reflected spectra across the platforms might differ (due to aircraft shape, size, and color), and considered these possible differences in our analyses.

# **Supplementary File S2**. Random UAS treatment schedule for UAS flights at the Erie County Landfill

| Day | Flight period | Random num. | Treatment rank |  | Number | Treatment |  |  |
| --- | --- | --- | --- | --- | --- | --- | --- | --- |
| 1 | morning | 0.15 | 5 |  | 1 | overhead multirotor |  |  |
| 1 | mid-day | 0.67 | 3 |  | 2 | targeted multirotor |  |  |
| 1 | afternoon | 0.95 | 1 |  | 3 | overhead fixed-wing |  |  |
| 2 | morning | 0.70 | 2 |  | 4 | targeted fixed-wing |  |  |
| 2 | mid-day | 0.13 | 6 |  | 5 | overhead ornithopter |  |  |
| 2 | afternoon | 0.54 | 4 |  | 6 | targeted ornithopter |  |  |
| 3 | morning | 0.52 | 2 |  |  |  |  |  |
| 3 | mid-day | 0.81 | 1 |  |  |  |  |  |
| 3 | afternoon | 0.51 | 3 |  |  |  |  |  |
| 4 | morning | 0.23 | 5 |  |  |  |  |  |
| 4 | mid-day | 0.46 | 4 |  |  |  |  |  |
| 4 | afternoon | 0.06 | 6 |  |  |  |  |  |
| 5 | morning | 0.28 | 6 |  |  |  |  |  |
| 5 | mid-day | 0.45 | 4 |  |  |  |  |  |
| 5 | afternoon | 0.67 | 1 |  |  |  |  |  |
| 6 | morning | 0.61 | 2 |  |  |  |  |  |
| 6 | mid-day | 0.44 | 5 |  |  |  |  |  |
| 6 | afternoon | 0.58 | 3 |  |  |  |  |  |
| 7 | morning | 0.98 | 1 |  |  |  |  |  |
| 7 | mid-day | 0.50 | 2 |  |  |  |  |  |
| 7 | afternoon | 0.29 | 5 |  |  |  |  |  |
| 8 | morning | 0.32 | 4 |  |  |  |  |  |
| 8 | mid-day | 0.17 | 6 |  |  |  |  |  |
| 8 | afternoon | 0.47 | 3 |  |  |  |  |  |
| 9 | morning | 0.03 | 6 |  |  |  |  |  |
| 9 | mid-day | 0.69 | 2 |  |  |  |  |  |
| 9 | afternoon | 0.46 | 4 |  |  |  |  |  |
| 10 | morning | 0.48 | 3 |  |  |  |  |  |
| 10 | mid-day | 0.93 | 1 |  |  |  |  |  |
| 10 | afternoon | 0.15 | 5 |  |  |  |  |  |
| 11 | morning | 0.40 | 5 |  |  |  |  |  |
| 11 | mid-day | 0.50 | 4 |  |  |  |  |  |
| 11 | afternoon | 0.34 | 6 |  |  |  |  |  |
| 12 | morning | 0.70 | 3 |  |  |  |  |  |
| 12 | mid-day | 0.96 | 1 |  |  |  |  |  |
| 12 | afternoon | 0.92 | 2 |  |  |  |  |  |
| 13 | morning | 0.04 | 6 |  |  |  |  |  |
| 13 | mid-day | 0.45 | 3 |  |  |  |  |  |
| 13 | afternoon | 1.00 | 1 |  |  |  |  |  |
| 14 | morning | 0.72 | 2 |  |  |  |  |  |
| 14 | mid-day | 0.20 | 5 |  |  |  |  |  |
| 14 | afternoon | 0.29 | 4 |  |  |  |  |  |
| 15 | morning | 0.44 | 6 |  |  |  |  |  |
| 15 | mid-day | 0.90 | 1 |  |  |  |  |  |
| 15 | afternoon | 0.61 | 2 |  |  |  |  |  |
| 16 | morning | 0.54 | 5 |  |  |  |  |  |
| 16 | mid-day | 0.57 | 4 |  |  |  |  |  |
| 16 | afternoon | 0.59 | 3 |  |  |  |  |  |
| 17 | morning | 0.90 | 2 |  |  |  |  |  |
| 17 | mid-day | 0.45 | 5 |  |  |  |  |  |
| 17 | afternoon | 0.32 | 6 |  |  |  |  |  |
| 18 | morning | 0.49 | 4 |  |  |  |  |  |
| 18 | mid-day | 0.93 | 1 |  |  |  |  |  |
| 18 | afternoon | 0.69 | 3 |  |  |  |  |  |
| 19 | morning | 0.44 | 4 |  |  |  |  |  |
| 19 | mid-day | 0.72 | 1 |  |  |  |  |  |
| 19 | afternoon | 0.46 | 3 |  |  |  |  |  |
| 20 | morning | 0.19 | 6 |  |  |  |  |  |
| 20 | mid-day | 0.61 | 2 |  |  |  |  |  |
| 20 | afternoon | 0.38 | 5 |  |  |  |  |  |
| 21 | morning | 0.38 | 4 |  |  |  |  |  |
| 21 | mid-day | 0.67 | 2 |  |  |  |  |  |
| 21 | afternoon | 0.55 | 3 |  |  |  |  |  |
| 22 | morning | 0.36 | 5 |  |  |  |  |  |
| 22 | mid-day | 0.33 | 6 |  |  |  |  |  |
| 22 | afternoon | 0.82 | 1 |  |  |  |  |  |
| 23 | morning | 0.16 | 5 |  |  |  |  |  |
| 23 | mid-day | 0.84 | 1 |  |  |  |  |  |
| 23 | afternoon | 0.19 | 4 |  |  |  |  |  |
| 24 | morning | 0.60 | 3 |  |  |  |  |  |
| 24 | mid-day | 0.68 | 2 |  |  |  |  |  |
| 24 | afternoon | 0.08 | 6 |  |  |  |  |  |
| 25 | morning | 0.08 | 6 |  |  |  |  |  |
| 25 | mid-day | 0.68 | 3 |  |  |  |  |  |
| 25 | afternoon | 0.30 | 5 |  |  |  |  |  |
| 26 | morning | 0.51 | 4 |  |  |  |  |  |
| 26 | mid-day | 0.89 | 2 |  |  |  |  |  |
| 26 | afternoon | 0.98 | 1 |  |  |  |  |  |
| 27 | morning | 0.56 | 2 |  |  |  |  |  |
| 27 | mid-day | 0.45 | 3 |  |  |  |  |  |
| 27 | afternoon | 0.08 | 5 |  |  |  |  |  |
| 28 | morning | 0.43 | 4 |  |  |  |  |  |
| 28 | mid-day | 0.03 | 6 |  |  |  |  |  |
| 28 | afternoon | 0.77 | 1 |  |  |  |  |  |
| 29 | morning | 0.24 | 3 |  |  |  |  |  |
| 29 | mid-day | 0.24 | 4 |  |  |  |  |  |
| 29 | afternoon | 0.17 | 5 |  |  |  |  |  |
| 30 | morning | 0.34 | 2 |  |  |  |  |  |
| 30 | mid-day | 0.35 | 1 |  |  |  |  |  |
| 30 | afternoon | 0.09 | 6 |  |  |  |  |  |
| 31 | morning | 0.18 | 5 |  |  |  |  |  |
| 31 | mid-day | 0.46 | 3 |  |  |  |  |  |
| 31 | afternoon | 0.67 | 2 |  |  |  |  |  |
| 32 | morning | 0.30 | 4 |  |  |  |  |  |
| 32 | mid-day | 0.98 | 1 |  |  |  |  |  |
| 32 | afternoon | 0.01 | 6 |  |  |  |  |  |
| 33 | morning | 0.21 | 4 |  |  |  |  |  |
| 33 | mid-day | 0.60 | 1 |  |  |  |  |  |
| 33 | afternoon | 0.40 | 3 |  |  |  |  |  |
| 34 | morning | 0.52 | 2 |  |  |  |  |  |
| 34 | mid-day | 0.14 | 5 |  |  |  |  |  |
| 34 | afternoon | 0.04 | 6 |  |  |  |  |  |
| 35 | morning | 0.08 | 5 |  |  |  |  |  |
| 35 | mid-day | 0.26 | 4 |  |  |  |  |  |
| 35 | afternoon | 0.77 | 2 |  |  |  |  |  |
| 36 | morning | 1.00 | 1 |  |  |  |  |  |
| 36 | mid-day | 0.56 | 3 |  |  |  |  |  |
| 36 | afternoon | 0.06 | 6 |  |  |  |  |  |
| 37 | morning | 0.70 | 1 |  |  |  |  |  |
| 37 | mid-day | 0.19 | 6 |  |  |  |  |  |
| 37 | afternoon | 0.37 | 4 |  |  |  |  |  |
| 38 | morning | 0.68 | 2 |  |  |  |  |  |
| 38 | mid-day | 0.23 | 5 |  |  |  |  |  |
| 38 | afternoon | 0.51 | 3 |  |  |  |  |  |
| 39 | morning | 0.19 | 5 |  |  |  |  |  |
| 39 | mid-day | 0.17 | 6 |  |  |  |  |  |
| 39 | afternoon | 0.50 | 3 |  |  |  |  |  |
| 40 | morning | 0.67 | 2 |  |  |  |  |  |
| 40 | mid-day | 0.88 | 1 |  |  |  |  |  |
| 40 | afternoon | 0.27 | 4 |  |  |  |  |  |
| 41 | morning | 0.64 | 2 |  |  |  |  |  |
| 41 | mid-day | 0.29 | 4 |  |  |  |  |  |
| 41 | afternoon | 0.39 | 3 |  |  |  |  |  |
| 42 | morning | 0.09 | 5 |  |  |  |  |  |
| 42 | mid-day | 0.97 | 1 |  |  |  |  |  |
| 42 | afternoon | 0.03 | 6 |  |  |  |  |  |
| 43 | morning | 0.84 | 1 |  |  |  |  |  |
| 43 | mid-day | 0.61 | 2 |  |  |  |  |  |
| 43 | afternoon | 0.21 | 5 |  |  |  |  |  |
| 44 | morning | 0.01 | 6 |  |  |  |  |  |
| 44 | mid-day | 0.57 | 3 |  |  |  |  |  |
| 44 | afternoon | 0.38 | 4 |  |  |  |  |  |
| 45 | morning | 0.68 | 3 |  |  |  |  |  |
| 45 | mid-day | 0.42 | 5 |  |  |  |  |  |
| 45 | afternoon | 0.97 | 1 |  |  |  |  |  |
| 46 | morning | 0.04 | 6 |  |  |  |  |  |
| 46 | mid-day | 0.63 | 4 |  |  |  |  |  |
| 46 | afternoon | 0.77 | 2 |  |  |  |  |  |
| 47 | morning | 0.37 | 6 |  |  |  |  |  |
| 47 | mid-day | 0.78 | 1 |  |  |  |  |  |
| 47 | afternoon | 0.39 | 5 |  |  |  |  |  |
| 48 | morning | 0.72 | 2 |  |  |  |  |  |
| 48 | mid-day | 0.71 | 3 |  |  |  |  |  |
| 48 | afternoon | 0.52 | 4 |  |  |  |  |  |

# **Supplementary File S3.** Sound intensity of UAS trials

The PIC was positioned 337 m away from the microphones and flew each UAS from East to West. We flew each treatment once and recorded the average dB for 10 seconds prior to passage of the UAS, which represented the maximum decibels recorded. Specifically, we assumed that the loudest dB reading was when the UAS was directly overhead of the sound level meter. However, we did not attempt to deviate flight patterns dynamically near the sound level meter to mimic sound associated with airframe wind resistance during banking. These possible sound differences could not necessarily be mimicked due to variability in altitudes and wind conditions during the actual study.

**Supplementary File S3 Table 1.** Raw mean decibels recorded for each treatment using a hand-held HP-882A Digital Sound Level Meter LCD Noise Measuring Instrument (HoldPeak, Guangdong, China).

| Treatment | Mean decibels (dB) ± standard error |
| --- | --- |
| overhead fixed-wing | 54.68 ± 0.81 |
| targeted fixed-wing | 55.73 ± 1.91 |
| overhead ornithopter | 56.11 ± 2.09 |
| targeted ornithopter | 56.24 ± 2.21 |
| overhead multirotor | 57.73 ± 1.85 |
| targeted multirotor | 57.40 ± 2.24 |

# **Supplementary File S4.** Post- processing of percent reflectance data from the three UAS

We collected over 600 light measurements from the three UAS using a Jaz portable spectroradiometer (Ocean Optics, Inc., Dunedin, FL, USA; Fig. 1). Each measurement ranged from 300 to 700 nm, was interpolated to 1-nm increments, was processed to adjust negative values, smoothed by a factor of 0.05, and were aggregated per UAS part. Due to spectrometer error, we were unable to collect ambient radiance data from our study site. Instead, we used measurements of absolute irradiance of the surrounding environment and the radiance of the sky and horizon from an open grassy field in West Lafayette, Indiana, USA (40.417370°N, -86.941781°W) on a partly cloudy day with 20% cloud cover (25 August 2015). The location and ambient light conditions do not pose issues for discerning perception, as the model calculations are relative to the visual physiology of the organism with the light conditions and reflectance from the UAS. For the absolute irradiance data, we averaged these measurements, converted from µWatt cm^-2^ to µmol s^-1^ m^-2^, and interpolated to 1-nm increments before applying them in the perceptual contrast models. The radiance of the sky and horizon data were averaged for each condition and interpolated to 1-nm increments before use in the perceptual contrast models. We partition UAS reflectance data to either a front or ventral perspective and used the horizon or sky radiance data as the respective backgrounds for contrasts.


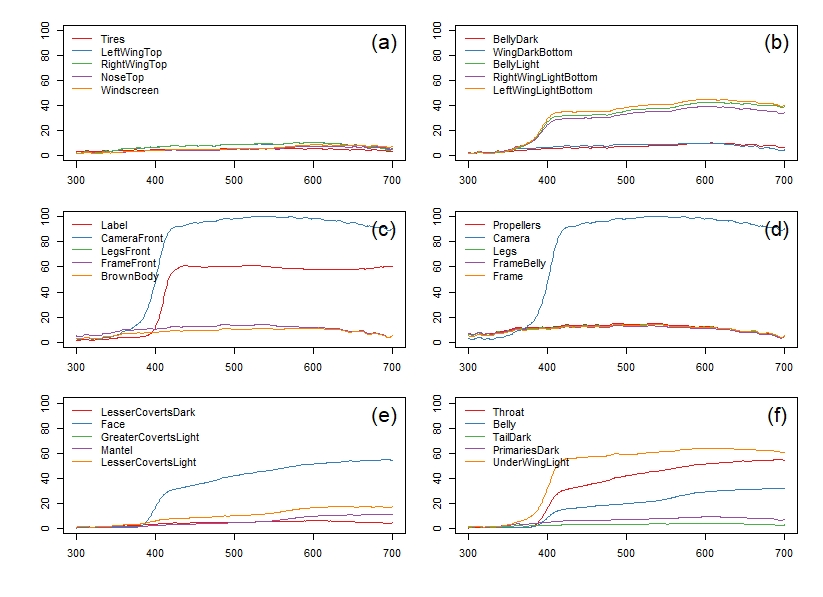


**Supplementary File S4 Figure 1.** Aggregated and smoothed by a factor of 0.05 percent reflectance data of UAS parts visible in regard to perspective; (a) front fixed-wing, (b) ventral fixed-wing, (c) front multirotor, (d) ventral multirotor, (e) front ornithopter, and (f) ventral ornithopter.

# **Supplementary File S5**. Georeferencing methodology

Videos of each trial were reviewed by two authors (MBP and BFB) in a blind study design in which MBP knew the treatment, but BFB was unaware of the treatment prior to watching the video. The video reviewers identified escape behavior for each turkey vulture on the ground for each trial; these frames were used to calculate FID. Both video reviewers had to agree on the image that contained the vulture’s first movement of escape in response to the approaching UAS. To be counted as a flight, the vulture had to carry through with a flight reaction to the approaching UAS (Fig. 1). To calculate speed of the treatment UAS, we exported two still frames taken prior to any vulture escape; we used Adobe Premiere Pro 2020 (version 14.2, Adobe Systems McLean, VA, USA) to export the frames.

Individual still frames were georeferenced by personnel unaware of the treatment (WSP USA, Worcester, MA, USA). Each image was added as a raster layer in ArcGIS Pro 2.4.3 (2019 ESRI, Redlands, California, USA). First, manual addition of control points to each raster was conducted by locating a point of interest on the raster to the referenced orthophoto created by the DJI Inspire (Main text Fig. 1). We conducted an automatic mapping mission of the landfill using the DJI Inspire after all trials were completed (05 September 2019) using the DJI Go App which automatically captured overlapping photographs (*n* = 145) of the study area. We did not use ground control points, however the orthomosaic resolution was 5.6cm/pixel. After manual addition of control points, the “Auto Georeference” function was used. This function added an additional 10-49 control points based on spectral signatures common between the georeferenced photograph and orthophoto. Each control point generated a residual spatial difference in comparison to the referenced orthophoto. Control points with a residual > 3 m were removed. Next, a spline transformation for local accuracy was performed on the control points and image. The transformation is based on a spline function—a piecewise polynomial that maintains continuity and smoothness between adjacent polynomials. The average Root Mean Square Error (RMSE), a measure of accuracy, was calculated. A full explanation of this process is illustrated in below. The georeferenced images were added to ArcGIS Pro and two authors (MBP and JLH) manually added features to a point shapefile at the location of the UAS and reacting vulture. We then used a customized ArcGIS model (Fig. 2) to calculate distance between multiple features in a point shapefile. Time, based on UAS position, between the two frames was recorded and converted from seconds:frames s^-1^ to seconds in order to calculate m s^-1^. We also collect speed measurements for approaches in which no vultures reacted.

| 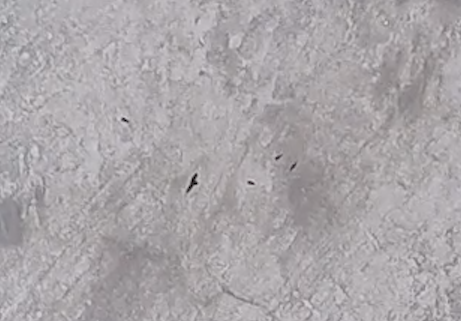  a) | 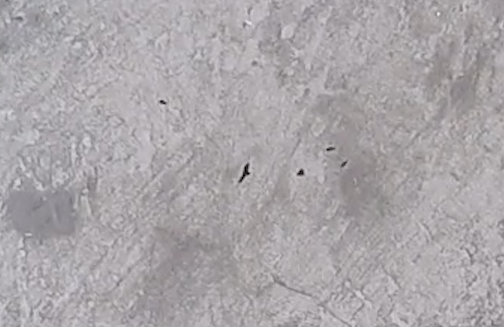  b) |
| --- | --- |

**Supplementary File S5 Figure 1.** Magnified still images from the 3DR Solo taken on 24 July 2019 (Run 28). a) shows five turkey vultures prior to any escape reactions. Four vultures have their wings held close to their bodies and one has its wings outstretched. b) shows the highlighted vulture raising its wings as the first step in escape from the oncoming UAS. Image b) was exported as the scene when this vulture initiated escape in order to estimate FID. Images were created by M.B.P.

The following georeferencing methodology utilizes the following software packages:

ArcGIS Pro version 2.4.3.

ESRI georeferencing support: <https://pro.arcgis.com/en/pro-app/help/data/imagery/georeferencing-a-raster-automatically-to-another-raster.htm>

Blue Marble Geographics Global Mapper version 21.

Website: <https://www.bluemarblegeo.com/products/global-mapper.php>

1. Launch ArcGIS pro and create a new Map.
2. Give the new project a name and location to be stored. Click “OK”.
3. A blank map opens. Add the orthophoto that the ungeoreferenced photos will be georeferenced to the map: “Map” tab -> “Add Data” -> “Data” -> navigate to file.
4. Highlight the orthophoto file in the table of contents. Select “Stretch Type” from the Raster Layer -> “Appearance” Tab and choose “None”. This ensures that the image pixel values are displayed as the image was at the time of creation and not being clipped or altered as to obscure ground features.
5. Add an ungeoreferenced photo to the map by selecting “Add Data” -> “Data” from the Map tab. Navigate to the folder where the images are stored and choose an image to be georeferenced.
6. The image will be added to the table of contents. Highlight the image, choose “Stretch Type from the “Appearance” tab and choose “None”. This ensures that the image pixel values are displayed as the image was at the time of creation and not being clipped or altered as to obscure objects in the image.
7. Highlight the image in the Table of Contents, and select “Georeference” from the “Imagery” tab. A number of tools will be activated. First, select “Fit to Display” and the ungeoreferenced image will be overlaid on the reference orthophoto.
8. Use the “Scale” function to make the ungeoreferenced image the approximate scale of the reference image.
9. Once the image is in relative position, click “Control Point Table” from the “Georeferencing” tab. A blank table will open.
10. Click “Add Control Points” from the “Georeference” Tab. This will allow the user to manually create a control points that will further align the image to the reference orthophoto. Objects that are on the ground surface were chosen to avoid vertical displacement that are common in both the ungeoreferenced image and the reference orthophoto image. The user first picks a point in the ungeoreferenced image and then picks the same point in the reference orthophoto image. This point will be then added to the Control Point Table. Points were chosen on the periphery of the ungeoreferenced photo to align the image to the referenced orthophoto and to distribute the manual control evenly throughout the image.
11. Continue to add points around the periphery of the ungeoreferenced image to further orient the image in relation to the reference orthophoto. We found that four points were sufficient to achieve an adequate initial georeference.
12. Once the image has been roughly oriented using manually picked control points, the user can run the “Auto Georeference” tool found in the “Georeference” tab. This tool computes control points based on the manual points already created and the spectral signatures common between both images. Due to the nature of the landfill changing throughout the duration of the study, WSP found that the Auto Georeference tool worked best when adding four manual control points before running the tool. Once run, the tool will created 10 - 40 control points and added them to the Control point table.
13. The user will then need to eliminate any control points with large residuals. The residual values can be sorted from high to low by clicking the “Residual” column header in the Control Point Table. A control point can be deleted by highlighting a control point in the table and clicking the “Delete Selected” button on the table toolbar. As the larger residuals are deleted, the image will adjust to further refine georeferencing.
14. Once the residuals are cleared to a value of < 3 meters and there are > 10 control points, a Spline transformation can be run on the control points. Up until this point, a 1^st^ Order Polynomial (Affine) Transformation has been used to georeferenced the image. The ESRI support states that “A first-order or affine transformation is used to shift, scale, and rotate a raster dataset. This generally results in straight lines on the raster dataset mapped as straight lines in the warped raster dataset. Thus, squares and rectangles on the raster dataset are commonly changed into parallelograms of arbitrary scaling and angle orientation.” ESRI support states that “The spline transformation is a true rubber sheeting method and optimizes for local accuracy but not global accuracy. It is based on a spline function—a piecewise polynomial that maintains continuity and smoothness between adjacent polynomials”.
15. Save as a new image with georeferencing written into image header. This saves the georeferencing information into the header of the image allowing geo-aware software to orient the image in real world coordinates (in this case WGS 1984 UTM Zone 17N meters).

On the Georeference tab choose “Save as New” and enter the following settings:

Output Raster Dataset: Choose a path where that image is to be stored and a name for the image. WSP appended a “_R” onto the image file name to differentiate the georeferenced and nongeoreferenced versions. Keep in mind that this version of ArcGIS Pro cannot accept “.” in filenames.

Coordinate System: WGS_1984_UTM_Zone_17N (derived from the base orthophoto).

Geographic Transformations: None.

Clipping Geometry: Default.

Maintain Clipping Extent: Unchecked.

Cell Size: Automatically updates upon entering Raster Size.

Raster Size: Columns 1920, Rows 1080 (the original image size).

Pixel Type: 8 Bit Unsigned.

NoData Value: blank (default).

Renderer Settings: Check “Force RGB”.

Output Format: TIFF.

Compression Type: None.

Compression Quality: Blank (Default).

WSP chose uncompressed .tif images as the output type to avoid losing image quality which some compression algorithms can introduce.

1. Export control points. The Georeference tab -> “Export Control Points” produces a text file of the control points in original photo coordinates and the final projected coordinates (WGS 1984 UTM Zone 17N Meters). Parameters for this export are navigating to an output directory and giving the .txt file a unique name. WSP used the naming convention of the rectified image appended with “_Control” as the filename.
2. Convert .txt files into ESRI .shp files. The text file of the exported control points contains four columns of data: x and y coordinates of the control point in original image coordinates and the x and y location of the control point in actual projected coordinates (WGS 1984 UTM Zone 17N Meters). Due to the number of control points for each image and the overall image count this process would need to be run in a batch. WDP used Global Mapper v21 to convert the text files into an ESRI .shp file to be used in ArcGIS Pro to be able to see where each control point was located in the image.
3. Open Global Mapper v21. Go to File -> “Batch Convert/Reproject”.
4. Select “ASCII Text” as File Type to Convert From.
5. Select “Shapefile” as File Type to Convert To.
6. Add .txt files to dialog through the “Add Files” button. Choose where to direct the destination files. WSP used the same file names appended with _Control. Since the incoming .txt files do not have a projection associated with them specify UTM Zone 17 / WGS84 / meters as the projection.
7. Set the parameters for the .txt import:

Import Type: Point Only (All Features are Points)

Coordinate Order: X / Easting / Longitude Coordinate First

Coordinate Format: Default (Decimal or Separated)

Coordinate Layout / Fields to Skip at Start of Line: 2

Coordinate Layout / Rows to Skip at Start of File: 0

Coordinate Layout / Coordinate Pairs Per Line: 1

Coordinate Line Prefix: None.

Check Use Selected Options for All ASCII Files

Coordinate Delimiter: Check Space or Tab

Leave all others default.

Click OK

1. Select project for output – select WGS84 UTM Zone 17N Meters.
2. Global Mapper creates shapefiles but they load as having an unknown coordinate system in ArcGIS Pro. To define a coordinate system, under Geoprocessing -> Toolboxes open the Data Management Tools -> Projections and Transformations -> right click “Define Projection” and select “Batch”.
3. Under Batch Define Projection, you can either create a temporary tool or save the batch tool. In this case we chose to save the tool because it will be used multiple times throughout the georeferencing process. Give the tool a name and location to be saved. Click “Next”.
4. On the next dialog, choose the files to define a projection and choose the coordinate system (WGS_1984_UTM_Zone_17N). Click “Run” and the tool will define the projection for the shapefiles


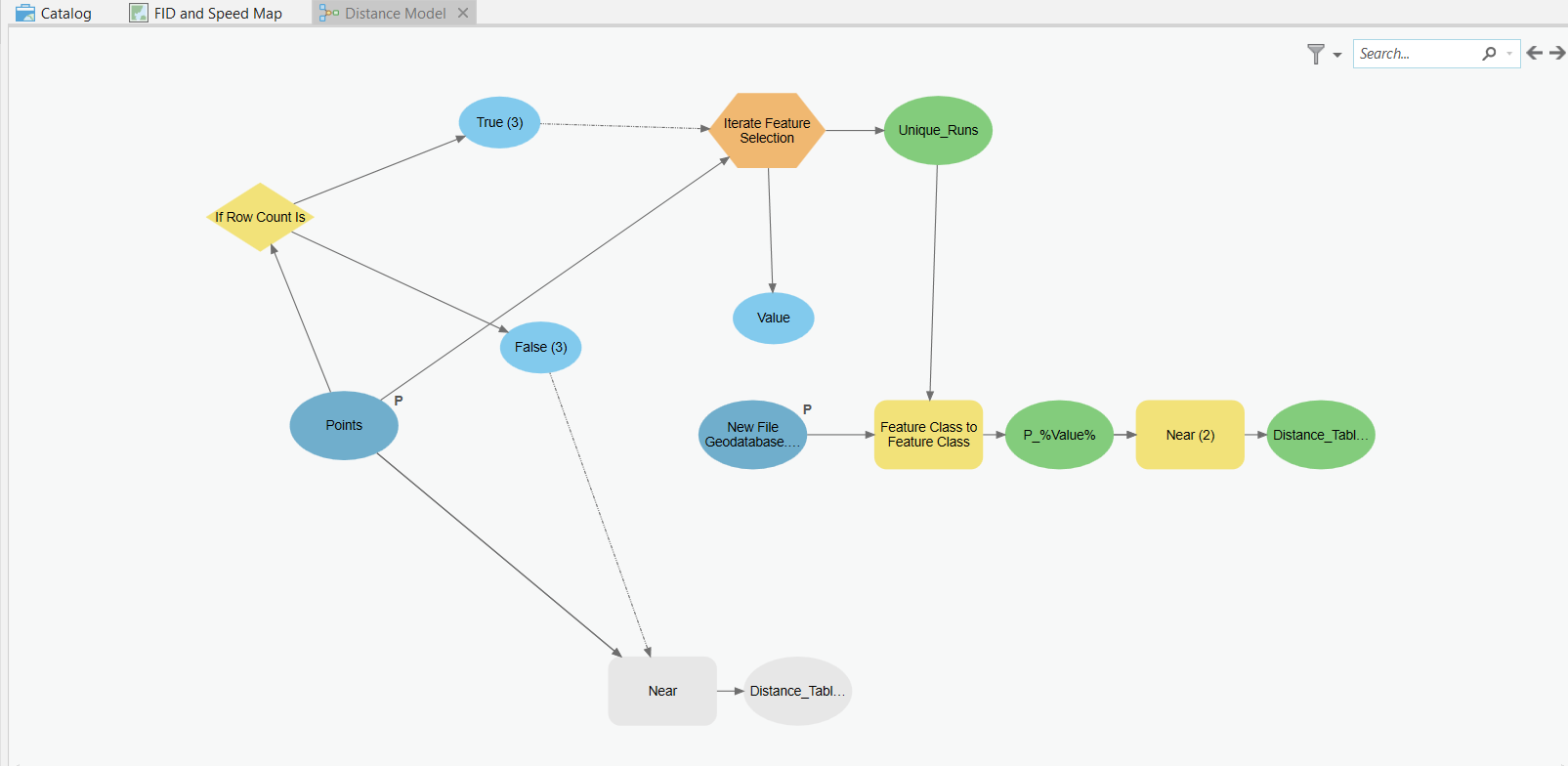


**Supplementary File S5 Figure 2**: ArcGIS model built for calculating multiple distances in our dataset

# **Supplementary File S6.** Pilot study protocol and power analyses

*2018 Pilot study protocol*

We first conducted a pilot study between 30 July and 03 August 2018 at the Erie County Landfill, Milan, Ohio (41.3434° N, -82.5966° W). The landfill is located 7 km south of Lake Erie and was frequented by groups of vultures in the summer ^1^. We flew an ornithopter and a fixed-wing UAS in an overhead or targeted pattern (see below). The ornithopter was a UAS modeled after a peregrine falcon (*Falco peregrinus*) that was powered by flapping flight and maneuvered using a mechanism in the tail (Clear Flight Solutions, Institutenweg, Netherlands). The fixed-wing platform was STAUFENBIEL WE CAN Fly 1460mm PNP (Staufenbiel/Horizon Hobby, Barsbüttel, Germany).

Given the short duration of the pilot study and the possibility of confounding results from treatments conducted sequentially (e.g., an ornithopter treatment might disperse most of the birds leaving no individuals for the next treatment), treatments were scheduled rather than assigned randomly. The order followed was: overhead fixed-wing, overhead ornithopter, targeted fixed-wing, and targeted ornithopter. Overhead treatments involved a gridded ‘lawn-mower’ back and forth movement above the focal birds at ~ 30 m AGL ^2^. Targeted treatments involved flying the UAS directly at focal turkey vultures that occasionally were surrounded by mixed flocks of herring (*Larus argentatus*) and ring-billed gulls (*L. delawarensis*). For overhead UAS treatments, the UAS pilot-in-command (PIC) did not target an individual bird, but for targeted UAS treatments, the PIC and observers identified one bird on the ground to target with a direct flight. The PIC was instructed to approach as close as possible to the focal bird in a direct flight without colliding. Focal birds were first chosen based on species (preference given to vultures) and then location in the grounded flock (e.g. perched higher).

We were able to approach the study site from below the position of the birds (16 m), climb to the top of the compressed landfill area ~100 m from the target area, and remain relatively obscured by the topography. Prior to launch of the UAS, the observers, timer, and PIC were positioned together southeast of the main active tipping face of the landfill. Before each UAS treatment, a rough count of gulls (mixed species) and vultures on the ground and in the air was recorded. To control for the visual presence of the UAS, the UAS platforms were covered with an opaque fabric sheet and mandatory pre-flight checks were conducted under this cover. Treatment time began when the UAS was revealed. Both UAS were launched by being manually thrown into the dominate wind direction. Duration of UAS treatments was ~ four minutes from reveal of the UAS to landing. Flights on a respective day were separated by at least 2hrs (~ 0900h, 1200h, 1400h) to reduce repeated measures on the same individuals.

At reveal and launch of the UAS, the timer announced ‘Start’ and started the stopwatch (Ultrak 499, Gardena, California, USA). Three observers each monitored an individual bird with binoculars and one observer monitored the focal vulture or gull. If a focal bird reacted in response to the UAS (see below), the observer communicated this immediately to the timer, who recorded the time to the nearest 10th of a second with the stopwatch. In the case that more than one bird reacted, the timer noted the order in which the reaction times were announced. A behavioral reaction in response to the UAS comprised a movement to the side (unrelated to foraging or conspecific interactions), relocation to another part of the landfill, or flying away from the landfill. The first behavioral reaction of the focal birds was recorded to the timer. We were unable to accurately determine alert response ^3^ because of the distance between the observers and birds. If after the four-minute treatment, a bird made no obvious behavioral reaction to the UAS, a ‘no reaction’ was noted. All stopwatch data were imported directly using the Ultrak software. For each treatment, we calculated the average reaction time for each species. Immediately after the UAS treatment, a block count of gulls and vultures on the ground and in the air was conducted. We calculated the vulture remaining index. Lower values correspond to a greater index of vultures dispersed.

Observers also scanned the area for bald eagles (*Haliaeetus leucocephalus*). Flights were not conducted if UAS operation would interfere with eagle movement or foraging in accordance with the Bald and Golden Eagle Protection Act. The PIC was licensed as a FAA Part 107 remote UAS pilot. Our methods were reviewed under the USDA National Wildlife Research Center (NWRC) QA-2963 and approved by the NWRC Institution Animal Care and Use Committee. No animals were injured during this study.

*Power analyses for the main study*

To adequately test our hypotheses and infer biological effects, we performed power analyses for each response variable (vulture remaining index, reaction time, FID, and latency to return) to determine necessary sample sizes. We conducted power analyses to determine the minimum number of UAS flights necessary to detect an effect of the treatment, given it exists, of UAS platform, approach and their interaction on the response metrics^4^. We did not set an *a priori* desired management effect. Instead, we used the results from the 2018 pilot study to directly calculate the effect size for each fixed effect and their interaction. We used the vulture remaining index from the pilot study to plan the main study, given that vultures remaining is suitable to measure effectiveness of UAS to disperse birds, and we had these data from the previous study. We did not conduct a power analysis for the binomial focal vulture reaction model given its similarities to the vulture remaining index and included the entire dataset. We calculated a range of effect sizes for vulture reaction time which included 1) the first vulture to react, 2) the average reaction time, and 3) a random vulture to react. For FID and latency to return, which were not collected in the 2018 pilot study, we searched the published literature for studies that used experimental designs to quantify bird response to vehicle approach. We only included studies that manipulated similar independent effects (e.g., approach and vehicle type). Because FID and bird body mass are positively correlated ^5^, we only used bird species that had a body mass within the upper percentile (25%) of a turkey vulture's body mass (1,505-2,508g). We found no studies that recorded latency for vultures to return to the study area after a vehicle approach. We, therefore, could not conduct a power analysis for latency to return.

We used general linear models (GLMs) to examine the response variables, with UAS approach and platform as fixed effects and the interaction between UAS approach and UAS platform. We used the partial eta^2^ from the GLM ANOVA statistics output to calculate effect sizes directly for each variable. This statistic is necessary for study comparisons involving more than one independent variable, where the sum of squares of each effect is in relation to the summation of the sum of squares for that effect and within-groups sum of squares ^6^. Specifically, the partial eta^2^ was used to determine the effect size *f* directly for each fixed effect and their interaction. This method will estimate the minimum sample size needed to achieve an effect or greater of the fixed effects on the response metrics.

We entered the partial eta2 from the GLM ANOVA summaries for each fixed effect and interaction directly into G*Power. Using an ANOVA (fixed effects, special, main effects and interactions) in the F test family in the statistical program G*Power (ver. 3.1.9.6 Universität, Germany), we calculated effect size for each fixed effect and the interaction term separately for 6 treatments (multirotor overhead, multirotor targeted, ornithopter overhead, ornithopter targeted, fixed-wing overhead, and fixed-wing targeted). Because the interaction term is what we were most interested in quantifying, we focused on the total sample size needed for that term.

We present the power analysis results for the vulture remaining index (Tables 1-2, Fig.1). We also present the range of effect and sample sizes for vulture reaction times (Tables 3-10). Lastly, we present the results of our literature review (Tables 11-14) for studies that measured FID of avian species of similar mass of turkey vultures in response to vehicle approach and type ^7-9^. For these analyses we used the ANOVA: Fixed effects, omnibus, one-way statistical test in G*Power. We entered the group sizes, means, and pooled standard deviations of the mentioned studies directly into G*Power.

**Supplementary File S6 Table 1.** ANOVA statistics for the vulture remaining index GLM.

| Variable | df | Sum^2^ | Mean^2^ | Statistic | P value | eta^2^ | Partial eta^2^ |
| --- | --- | --- | --- | --- | --- | --- | --- |
| UAS platform | 1 | 3.801 | 3.801 | 6.951 | 0.030 | 0.416 | 0.465 |
| approach | 1 | 0.212 | 0.212 | 0.387 | 0.551 | 0.023 | 0.046 |
| platform * approach | 1 | 0.756 | 0.756 | 1.383 | 0.273 | 0.083 | 0.147 |
| Residuals | 8 | 4.375 | 0.547 | NA | NA | NA | NA |

**Supplementary File S6 Table 2.** G*Power protocol for the fixed effects ([1] = UAS platform, [2] = approach, [3] = platform * approach) of the vulture remaining index response variable.

[1] -- Tuesday, February 09, 2021 -- 10:24:29

F tests - ANOVA: Fixed effects, special, main effects and interactions

**Analysis:** A priori: Compute required sample size

**Input:** Effect size f = 0.9322869

α err prob = 0.05

Power (1-β err prob) = 0.95

Numerator df = 2

Number of groups = 6

**Output:** Noncentrality parameter λ = 19.1214950

Critical F = 3.6337235

Denominator df = 16

Total sample size = 22

Actual power = 0.9531422

[2] -- Tuesday, February 09, 2021 -- 10:24:54

F tests - ANOVA: Fixed effects, special, main effects and interactions

**Analysis:** A priori: Compute required sample size

**Input:** Effect size f = 0.2195860

α err prob = 0.05

Power (1-β err prob) = 0.95

Numerator df = 1

Number of groups = 6

**Output:** Noncentrality parameter λ = 13.1152991

Critical F = 3.8766551

Denominator df = 266

Total sample size = 272

Actual power = 0.9503683

[3] -- Tuesday, February 09, 2021 -- 10:25:09

F tests - ANOVA: Fixed effects, special, main effects and interactions

**Analysis:** A priori: Compute required sample size

**Input:** Effect size f = 0.4151300

α err prob = 0.05

Power (1-β err prob) = 0.95

Numerator df = 2

Number of groups = 6

**Output:** Noncentrality parameter λ = 16.0269613

Critical F = 3.1012958

Denominator df = 87

Total sample size = 93

Actual power = 0.9504591

**Supplementary File S6 Figure 1.** G*Power graphical output for the total sample size for discerning a biological effect on the vulture remaining index relative to the interaction of UAS platform and approach.

**Supplementary File S6 Table 3.** ANOVA statistics for the first vulture reaction time GLM.

| term | df | Sum^2^ | Mean^2^ | Statistic | P value | eta^2^ | Partial eta^2^ |
| --- | --- | --- | --- | --- | --- | --- | --- |
| UAS platform | 1 | 79.822 | 79.822 | 0.966 | 0.381 | 0.144 | 0.195 |
| approach | 1 | 113.002 | 113.002 | 1.368 | 0.307 | 0.204 | 0.255 |
| platform * approach | 1 | 29.977 | 29.977 | 0.363 | 0.579 | 0.054 | 0.083 |
| Residuals | 4 | 330.524 | 82.631 | NA | NA | NA | NA |

**Supplementary File S6 Table 4.** G*Power protocol for the fixed effects ([1] = UAS platform, [2] = approach, [3] = platform * approach) of the first vulture reaction time response variable.

[1] -- Tuesday, February 09, 2021 -- 10:32:30

F tests - ANOVA: Fixed effects, special, main effects and interactions

**Analysis**: A priori: Compute required sample size

**Input:** Effect size f = 0.4921748

α err prob = 0.05

Power (1-β err prob) = 0.95

Numerator df = 2

Number of groups = 6

**Output:** Noncentrality parameter λ = 16.4720503

Critical F = 3.1452584

Denominator df = 62

Total sample size = 68

Actual power = 0.9530050

[2] -- Tuesday, February 09, 2021 -- 10:32:46

F tests - ANOVA: Fixed effects, special, main effects and interactions

**Analysis:** A priori: Compute required sample size

**Input:** Effect size f = 0.5850486

α err prob = 0.05

Power (1-β err prob) = 0.95

Numerator df = 1

Number of groups = 6

**Output:** Noncentrality parameter λ = 14.0335564

Critical F = 4.1213382

Denominator df = 35

Total sample size = 41

Actual power = 0.9536708

[3] -- Tuesday, February 09, 2021 -- 10:33:07

F tests - ANOVA: Fixed effects, special, main effects and interactions

**Analysis:** A priori: Compute required sample size

**Input:** Effect size f = 0.3008530

α err prob = 0.05

Power (1-β err prob) = 0.95

Numerator df = 2

Number of groups = 6

**Output:** Noncentrality parameter λ = 15.7491798

Critical F = 3.0497921

Denominator df = 168

Total sample size = 174

Actual power = 0.9503346

**Supplementary File S6 Table 5.** ANOVA statistics for the average vulture reaction time GLM.

| term | df | Sum^2^ | Mean^2^ | Statistic | P value | eta^2^ | Partial eta^2^ |
| --- | --- | --- | --- | --- | --- | --- | --- |
| UAS platform | 1 | 2022.719 | 2022.719 | 0.690 | 0.453 | 0.103 | 0.147 |
| approach | 1 | 2865.124 | 2865.124 | 0.977 | 0.379 | 0.146 | 0.196 |
| platform * approach | 1 | 3075.034 | 3075.034 | 1.049 | 0.364 | 0.156 | 0.208 |
| Residuals | 4 | 11727.790 | 2931.947 | NA | NA | NA | NA |
|  |  |  |  |  |  |  |  |

**Supplementary File S6 Table 6.** G*Power protocol for the fixed effects ([1] = UAS platform, [2] = approach, [3] = platform * approach) of the average vulture reaction time response variable.

[1] -- Tuesday, February 09, 2021 -- 10:36:34

F tests - ANOVA: Fixed effects, special, main effects and interactions

**Analysis:** A priori: Compute required sample size

**Input:** Effect size f = 0.4151300

α err prob = 0.05

Power (1-β err prob) = 0.95

Numerator df = 2

Number of groups = 6

**Output:** Noncentrality parameter λ = 16.0269613

Critical F = 3.1012958

Denominator df = 87

Total sample size = 93

Actual power = 0.9504591

[2] -- Tuesday, February 09, 2021 -- 10:37:00

F tests - ANOVA: Fixed effects, special, main effects and interactions

**Analysis**: A priori: Compute required sample size

**Input:** Effect size f = 0.4937419

α err prob = 0.05

Power (1-β err prob) = 0.95

Numerator df = 1

Number of groups = 6

**Output:** Noncentrality parameter λ = 13.6517396

Critical F = 4.0343097

Denominator df = 50

Total sample size = 56

Actual power = 0.9518444

[3] -- Tuesday, February 09, 2021 -- 10:37:10

F tests - ANOVA: Fixed effects, special, main effects and interactions

**Analysis:** A priori: Compute required sample size

**Input:** Effect size f = 0.5124707

α err prob = 0.05

Power (1-β err prob) = 0.95

Numerator df = 2

Number of groups = 6

**Output:** Noncentrality parameter λ = 16.5454518

Critical F = 3.1588427

Denominator df = 57

Total sample size = 63

Actual power = 0.9530207

**Supplementary File S6 Table 7.** ANOVA statistics for the random vulture reaction time GLM.

| term | df | Sum^2^ | Mean^2^ | Statistic | P value | eta^2^ | Partial eta^2^ |
| --- | --- | --- | --- | --- | --- | --- | --- |
| UAS platform | 1 | 4608.000 | 4608.000 | 1.143 | 0.345 | 0.154 | 0.222 |
| approach | 1 | 5262.446 | 5262.446 | 1.305 | 0.317 | 0.176 | 0.246 |
| platform * approach | 1 | 3963.166 | 3963.166 | 0.983 | 0.378 | 0.132 | 0.197 |
| Residuals | 4 | 16126.421 | 4031.605 | NA | NA | NA | NA |
|  |  |  |  |  |  |  |  |

**Supplementary File S6 Table 8.** G*Power protocol for the fixed effects ([1] = UAS platform, [2] = approach, [3] = platform * approach) of the average vulture reaction time response variable.

[1] -- Tuesday, February 09, 2021 -- 10:39:47

F tests - ANOVA: Fixed effects, special, main effects and interactions

**Analysis:** A priori: Compute required sample size

**Input:** Effect size f = 0.5341788

α err prob = 0.05

Power (1-β err prob) = 0.95

Numerator df = 2

Number of groups = 6

**Output:** Noncentrality parameter λ = 16.5501254

Critical F = 3.1751410

Denominator df = 52

Total sample size = 58

Actual power = 0.9520786

[2] -- Tuesday, February 09, 2021 -- 10:40:05

F tests - ANOVA: Fixed effects, special, main effects and interactions

**Analysis:** A priori: Compute required sample size

**Input:** Effect size f = 0.5711917

α err prob = 0.05

Power (1-β err prob) = 0.95

Numerator df = 1

Number of groups = 6

**Output:** Noncentrality parameter λ = 14.0291782

Critical F = 4.1054559

Denominator df = 37

Total sample size = 43

Actual power = 0.9541708

[3] -- Tuesday, February 09, 2021 -- 10:40:25

F tests - ANOVA: Fixed effects, special, main effects and interactions

**Analysis:** A priori: Compute required sample size

**Input**: Effect size f = 0.4953080

α err prob = 0.05

Power (1-β err prob) = 0.95

Numerator df = 2

Number of groups = 6

**Output:** Noncentrality parameter λ = 16.4371110

Critical F = 3.1477912

Denominator df = 61

Total sample size = 67

Actual power = 0.9524451

**Supplementary File S6 Table 9.** Range of effect sizes for vulture reaction times to UAS approach, UAS platform, and the interaction term. Data are arranged in ascending order by effect size of the interaction term.

| Variable | First vulture | Random vulture | Average vulture |
| --- | --- | --- | --- |
| UAS platform | 0.49 | 0.53 | 0.42 |
| approach | 0.59 | 0.57 | 0.49 |
| platform * approach | 0.30 | 0.50 | 0.51 |

**Supplementary File S6 Table 10.** Range of sample sizes for vulture reaction times to UAS approach, UAS platform, and the interaction term. Data are arranged in ascending order by sample size of the interaction term.

| Variable | Average vulture | Random vulture | First vulture |
| --- | --- | --- | --- |
| UAS platform | 93 | 58 | 68 |
| approach | 56 | 43 | 41 |
| platform * approach | 63 | 67 | 174 |

**Supplementary File S6 Table 11.** Descriptive statistics for bird response to a manipulated vehicle approach. Data are from^9^. Mass comparison (mass comp.) is the percent difference from a turkey vulture’s weight.

| species | Mass comp. | Grp 1 n | Grp 1 mean | Grp 1 SD | Grp 2 n | Grp 2 mean | Grp 2 SD | Pooled SD |
| --- | --- | --- | --- | --- | --- | --- | --- | --- |
| Australian white ibis (*Threskiornis molucca*) | 10 | 9 | 52.6 | 18.9 | 9 | 27.4 | 21 | 20 |

**Supplementary File S6 Table 12.** G*Power protocol for FID of the Australian white ibis (*Threskiornis molucca*) to UAS approach (independent effect: 4 m vs. 10 m AGL). Data are from ^9^.

[7] -- Monday, February 08, 2021 -- 16:02:01

F tests - ANOVA: Fixed effects, omnibus, one-way

**Analysis:** A priori: Compute required sample size

**Input:** Effect size f = 0.6300000

α err prob = 0.05

Power (1-β err prob) = 0.95

Number of groups = 2

**Output:** Noncentrality parameter λ = 14.2884000

Critical F = 4.1300177

Numerator df = 1

Denominator df = 34

Total sample size = 36

Actual power = 0.9564837

**Supplementary File S6 Table 13.** Descriptive statistics for bird response to different vehicle types. Data are from ^7-8^ and arranged in ascending order by sample size. Mass comparison (mass comp.) is the percent difference from a vulture’s weight.

| species | Mass comp. | Grp 1 n | Grp 1 mean | Grp 1 SD | Grp 2 n | Grp 2 mean | Grp 2 SD | Pooled SD |
| --- | --- | --- | --- | --- | --- | --- | --- | --- |
| Australian white ibis | 10 | 8 | 55.10 | 22.40 | 6 | 64.90 | 43.00 | 32.60 |
| Double-crested cormorant (*Phalacrocorax auritus*) | 17 | 90 | 49.45 | 25.68 | 73 | 42.76 | 19.94 | 23.29 |
| Great blue heron (*Ardea herodias*) | 14 | 125 | 49.52 | 22.72 | 93 | 42.16 | 20.27 | 21.71 |

**Supplementary File S6 Table 14.** G*Power protocol for FID of Australian white ibis to vehicle type ([1] independent effect: car vs. bus), double-crested cormorant ([2] independent effect: personal watercraft vs. boat) and great blue heron ([3] independent effect: personal watercraft vs. boat). Data are from ^7-8^.

[1] -- Tuesday, February 09, 2021 -- 10:12:30

F tests - ANOVA: Fixed effects, omnibus, one-way

**Analysis:** A priori: Compute required sample size

**Input:** Effect size f = 0.1487651

α err prob = 0.05

Power (1-β err prob) = 0.95

Number of groups = 2

**Output:** Noncentrality parameter λ = 13.0573224

Critical F = 3.8573221

Numerator df = 1

Denominator df = 588

Total sample size = 590

Actual power = 0.9502846

[2] -- Tuesday, February 09, 2021 -- 10:14:38

F tests - ANOVA: Fixed effects, omnibus, one-way

**Analysis:** A priori: Compute required sample size

**Input:** Effect size f = 0.1428406

α err prob = 0.05

Power (1-β err prob) = 0.95

Number of groups = 2

**Output:** Noncentrality parameter λ = 13.0581997

Critical F = 3.8560754

Numerator df = 1

Denominator df = 638

Total sample size = 640

Actual power = 0.9503446

[3] -- Tuesday, February 09, 2021 -- 10:15:55

F tests - ANOVA: Fixed effects, omnibus, one-way

**Analysis:** A priori: Compute required sample size

**Input:** Effect size f = 0.1676710

α err prob = 0.05

Power (1-β err prob) = 0.95

Number of groups = 2

**Output:** Noncentrality parameter λ = 13.1009209

Critical F = 3.8615778

Numerator df = 1

Denominator df = 464

Total sample size = 466

Actual power = 0.9507382

References

1 Belant, J. L., Seamans, T. W., Gabrey, S. W. & Dolbeer, R. A. Abundance of gulls and other birds at landfills in northern Ohio. *Am. Midl. Nat.* **134**, 30-40 (1995).

2 Mulero-Pázmány, M. *et al.* Unmanned aircraft systems as a new source of disturbance for wildlife: A systematic review. *PloS one* **12**, e0178448 (2017).

3 Fernández-Juricic, E., Jimenez, M. D. & Lucas, E. Alert distance as an alternative measure of bird tolerance to human disturbance: implications for park design. *Environ. Conserv.* **28**, 263-269, doi:10.1017/S0376892901000273 (2001).

4 Johnson, P. C., Barry, S. J., Ferguson, H. M. & Müller, P. Power analysis for generalized linear mixed models in ecology and evolution. *Methods Ecol. Evol.* **6**, 133-142 (2015).

5 Blumstein, D. T. Developing an evolutionary ecology of fear: how life history and natural history traits affect disturbance tolerance in birds. *Anim. Behav.* **71**, 389-399, doi:https://doi.org/10.1016/j.anbehav.2005.05.010 (2006).

6 Lakens, D. Calculating and reporting effect sizes to facilitate cumulative science: a practical primer for t-tests and ANOVAs. *Front. Psychol.* **4**, 863 (2013).

7 McLeod, E. M., Guay, P.-J., Taysom, A. J., Robinson, R. W. & Weston, M. A. Buses, cars, bicycles and walkers: the influence of the type of human transport on the flight responses of waterbirds. *PLoS One* **8**, e82008 (2013).

8 Rodgers, J. A. & Schwikert, S. T. Buffer‐zone distances to protect foraging and loafing waterbirds from disturbance by personal watercraft and outboard‐powered boats. *Conserv. Biol.* **16**, 216-224 (2002).

9 Weston, M. A., O’Brien, C., Kostoglou, K. N. & Symonds, M. R. Escape responses of terrestrial and aquatic birds to drones: Towards a code of practice to minimize disturbance. *J. Appl. Ecol.* **57**, 777-785 (2020).

# **Supplementary File S7.** Temporal autocorrelation results

**Supplementary File S7 Table 1.** Akaike Information Criterion (AIC) model ranking for models with compound symmetry temporal autocorrelation and without temporal autocorrelation. Models in bold were ranked the highest.

|  | df | AIC |
| --- | --- | --- |
| **Binomial focal vulture reaction** |  |  |
| **Without autocorrelation** | **11** | **180.24** |
| Compound symmetry with autocorrelation | 12 | 182.24 |
| **Focal vulture reaction time** |  |  |
| **Without autocorrelation** | **9** | **387.68** |
| Compound symmetry with autocorrelation | 10 | 389.68 |
| **Focal vulture FID** |  |  |
| **Without autocorrelation** | **8** | **337.23** |
| Compound symmetry with autocorrelation | 9 | 339.23 |
| **Vulture remaining index** |  |  |
| **Without autocorrelation** | **10** | **211.42** |
| Compound symmetry with autocorrelation | 11 | 213.42 |
| **Latency to return** |  |  |
| **Without autocorrelation** | **8** | **294.03** |
| Compound symmetry with autocorrelation | 9 | 296.03 |


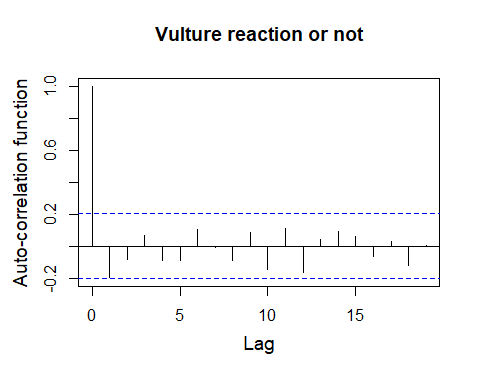


**Supplementary File S7 Figure 1.** Autocorrelation plot for residuals for if the vulture reacted or not. The dottle blue lines are thresholds for correlation.


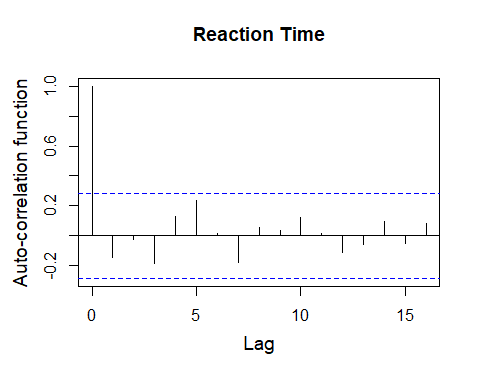


**Supplementary File S7 Figure 2.** Autocorrelation plot for residuals for focal vulture reaction time (s). The dottle blue lines are thresholds for correlation.


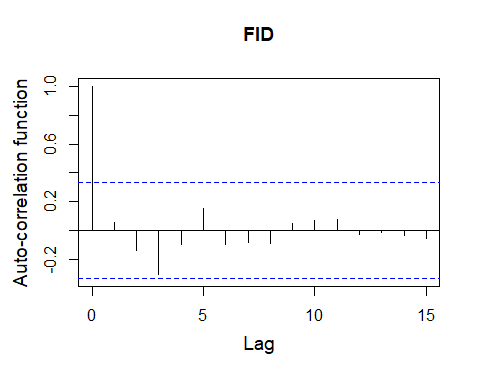


**Supplementary File S7 Figure 3.** Autocorrelation plot for residuals for focal vulture FID (m). The dottle blue lines are thresholds for correlation.


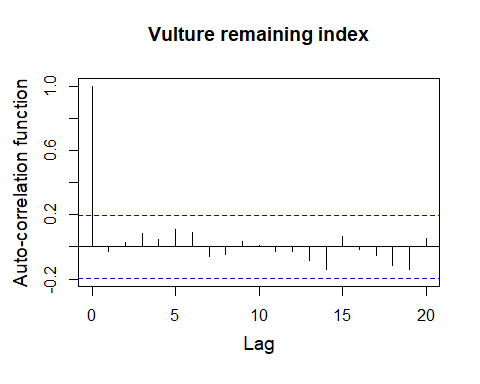


**Supplementary File S7 Figure 4.** Autocorrelation plot for residuals for vulture remaining index. The dottle blue lines are thresholds for correlation.


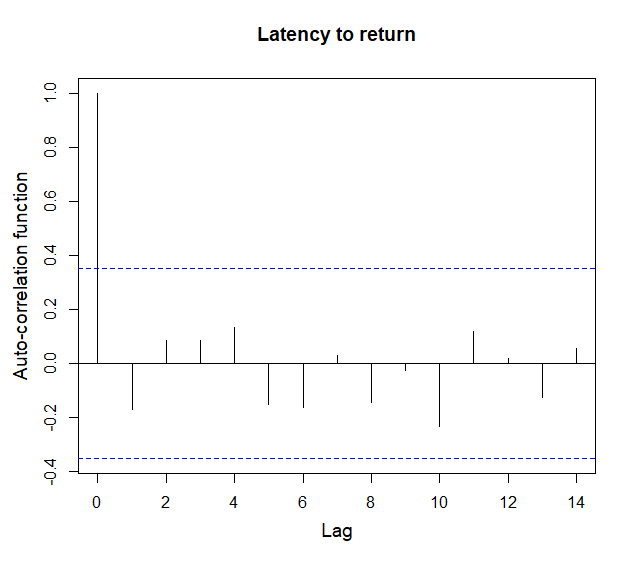


**Supplementary File S7 Figure 5.** Autocorrelation plot for residuals for latency to return (s). The dottle blue lines are thresholds for correlation.

# **Supplementary File S8.** R Code for statistical analyses

Pfeiffer et al. R Code

Pfeiffer et al. in prep.

24 May, 2021

# Code for Pfeiffer et al in prep

# clear memory
rm(list = ls ())

df <- read.csv(file.choose()) #Load Pfeiffer_etal_ArchivedDataset.csv
df<-read.csv("Pfeiffer_etal_ArchivedDataset.csv")
#require the following packages
#Install if you don't have already
if (!require("car")) install.packages("car")

## Loading required package: car

## Loading required package: carData

library(car)
if (!require("gvlma")) install.packages("gvlma")

## Loading required package: gvlma

library(gvlma)
if (!require("plyr")) install.packages("plyr")

## Loading required package: plyr

library(plyr)
if (!require("dplyr")) install.packages("dplyr")

## Loading required package: dplyr

##
## Attaching package: 'dplyr'

## The following objects are masked from 'package:plyr':
##
## arrange, count, desc, failwith, id, mutate, rename, summarise,
## summarize

## The following object is masked from 'package:car':
##
## recode

## The following objects are masked from 'package:stats':
##
## filter, lag

## The following objects are masked from 'package:base':
##
## intersect, setdiff, setequal, union

library(dplyr)
if (!require("ggplot2")) install.packages("ggplot2")

## Loading required package: ggplot2

library(ggplot2)
if (!require("lme4")) install.packages("lme4")

## Loading required package: lme4

## Loading required package: Matrix

## Registered S3 methods overwritten by 'lme4':
## method from
## cooks.distance.influence.merMod car
## influence.merMod car
## dfbeta.influence.merMod car
## dfbetas.influence.merMod car

library(lme4)
if (!require("nlme")) install.packages("nlme")

## Loading required package: nlme

##
## Attaching package: 'nlme'

## The following object is masked from 'package:lme4':
##
## lmList

## The following object is masked from 'package:dplyr':
##
## collapse

library(nlme)
if (!require("psych")) install.packages("psych")

## Loading required package: psych

##
## Attaching package: 'psych'

## The following objects are masked from 'package:ggplot2':
##
## %+%, alpha

## The following object is masked from 'package:car':
##
## logit

library(psych)
if (!require("moments")) install.packages("moments")

## Loading required package: moments

library(moments)
if (!require("cowplot")) install.packages("cowplot")

## Loading required package: cowplot

library(cowplot)
if (!require("emmeans")) install.packages("emmeans")

## Loading required package: emmeans

library(emmeans)

# Settings for model interpretation
# Default sums of squares
options(contrasts = c("contr.treatment", "contr.poly"))

# Necessary to conduct type III sums of squares
options(contrasts = c("contr.sum", "contr.poly"))

# Read Date column to
# Portable Operating System Interface (POSIX) calender time (ct)
dts1<-paste(df$DateOriginal,df$RevealTime)
dts2<-as.POSIXct(dts1,format="%m.%d.%Y %H:%M")
dt<-data.frame(df,ct=dts2)

# check POSIX and create day column
range(dt$ct)

## [1] "2019-07-08 02:08:00 EDT" "2019-09-03 08:33:00 EDT"

# covert wind speed to mps
dt$WindSpeedmps<-dt$WindSpeed/(3.6)

# calculate the number of Herring and Ring-billed Gulls on
# the ground prior to treatment
dt$Bgulls<-dt$bHERGground+dt$bRBGUground

# calculate the vulture remaining index (remaining)
dt$remaining<-(dt$aTUVUground)/(dt$bTUVUground)

###########Binary vulture reaction or not########################
# create a binary model of focal bird reaction or not
binaryDt <- subset.data.frame(
 dt, FocalReactionOrNot == 0 | FocalReactionOrNot == 1 )
binaryDt$FocalReactionOrNot<-as.numeric(
 as.character(binaryDt$FocalReactionOrNot))

as.numeric(binaryDt$FocalReactionOrNot)

## [1] 1 0 0 1 0 1 0 0 1 1 0 1 1 0 0 1 0 1 0 1 1 0 1 1 1 1 1 0 1 0 1 0 1 0 0 1 1 1
## [39] 1 1 0 1 1 0 1 1 0 1 1 0 1 1 0 1 1 1 1 0 0 1 1 1 0 0 1 0 0 0 1 1 1 1 1 1 0 1
## [77] 0 1 1 1 0 1 1 1 1 0 1 0 1 1 0 1 0 1

is.numeric(binaryDt$FocalReactionOrNot)

## [1] TRUE

# look at sample per treatment
nrow(binaryDt)

## [1] 94

table(binaryDt$Drone2, binaryDt$Approach)

##
## Overhead Targeted
## Fixed-wing 18 17
## Multirotor 16 15
## Ornithopter 14 14

# look at overall results
table(binaryDt$Drone2,binaryDt$FocalReactionOrNot)

##
## 0 1
## Fixed-wing 9 26
## Multirotor 10 21
## Ornithopter 16 12

table(binaryDt$FocalReactionOrNot)

##
## 0 1
## 35 59

# making sure that drone and approach are factors
binaryDt$Drone2 <- as.factor(binaryDt$Drone2)
binaryDt$Approach <- as.factor(binaryDt$Approach)

# build models with and without temporal auto-correlation
M0=gls(FocalReactionOrNot~Drone2*Approach+AmbLight+
 bTUVUground+Bgulls+WindSpeedmps,binaryDt)
M0cor1=gls(FocalReactionOrNot~Drone2*Approach+AmbLight+
 bTUVUground+Bgulls+WindSpeedmps,binaryDt,
 correlation=corCompSymm(form=~sort(ct)))

# rank models by AIC
AIC(M0, M0cor1)

## df AIC
## M0 11 180.2379
## M0cor1 12 182.2379

# plot response over time
ggplot(data=binaryDt,aes(x=ct,y=FocalReactionOrNot))+
 geom_point()+theme_classic()+
 labs(x="Date",y="Vulture reaction or not")

# code for Supplementary File S7 Figure 1

# auto-correlation plot
acf(residuals(M0, type="normalized"),
 main="Vulture reaction or not", ylab="", xlab="")
mtext("Lag", 1, line= 2.5, cex=1.2)
mtext("Auto-correlation function", 2, line= 2.5, cex=1.2)

## No evidence of temporal auto-correlation

# check association between SD and the fixed effects
Anova(lm(StartingDistance ~ Drone2*Approach, binaryDt),
 type="3")

## Anova Table (Type III tests)
##
## Response: StartingDistance
## Sum Sq Df F value Pr(>F)
## (Intercept) 6678325 1 2045.9176 < 2e-16 ***
## Drone2 29665 2 4.5440 0.01324 *
## Approach 250 1 0.0765 0.78278
## Drone2:Approach 11779 2 1.8043 0.17062
## Residuals 287251 88
## ---
## Signif. codes: 0 '***' 0.001 '**' 0.01 '*' 0.05 '.' 0.1 ' ' 1

# SD is associated with drone, but not approach

# create linear model and run ANOVA
SD.lm <- lm(StartingDistance ~ Drone2*Approach, binaryDt)
means.SD <- emmeans(SD.lm, "Drone2", type = "response")

## NOTE: Results may be misleading due to involvement in interactions

means.SD

## Drone2 emmean SE df lower.CL upper.CL
## Fixed-wing 254 9.66 88 234 273
## Multirotor 293 10.27 88 272 313
## Ornithopter 257 10.80 88 235 278
##
## Results are averaged over the levels of: Approach
## Confidence level used: 0.95

pairs(means.SD)

## contrast estimate SE df t.ratio p.value
## (Fixed-wing) - Multirotor -39.12 14.1 88 -2.775 0.0183
## (Fixed-wing) - Ornithopter -3.19 14.5 88 -0.220 0.9736
## Multirotor - Ornithopter 35.93 14.9 88 2.411 0.0468
##
## Results are averaged over the levels of: Approach
## P value adjustment: tukey method for comparing a family of 3 estimates

#Multirotor reached further than the other drones

# check association between speed and the fixed effects
Anova(lm(speed1 ~ Drone2*Approach, binaryDt), type="3")

## Anova Table (Type III tests)
##
## Response: speed1
## Sum Sq Df F value Pr(>F)
## (Intercept) 19327.3 1 788.2868 < 2e-16 ***
## Drone2 211.1 2 4.3043 0.01669 *
## Approach 26.0 1 1.0605 0.30613
## Drone2:Approach 96.5 2 1.9675 0.14635
## Residuals 2010.5 82
## ---
## Signif. codes: 0 '***' 0.001 '**' 0.01 '*' 0.05 '.' 0.1 ' ' 1

# drone speed is associated with drone, but not approach

# create linear model and run ANOVA
SD.lm <- lm(speed1 ~ Drone2*Approach, binaryDt)
means.SD <- emmeans(SD.lm, "Drone2", type = "response")

## NOTE: Results may be misleading due to involvement in interactions

means.SD

## Drone2 emmean SE df lower.CL upper.CL
## Fixed-wing 17.0 0.862 82 15.3 18.7
## Multirotor 14.5 0.920 82 12.6 16.3
## Ornithopter 13.3 0.974 82 11.4 15.2
##
## Results are averaged over the levels of: Approach
## Confidence level used: 0.95

pairs(means.SD)

## contrast estimate SE df t.ratio p.value
## (Fixed-wing) - Multirotor 2.53 1.26 82 2.002 0.1180
## (Fixed-wing) - Ornithopter 3.67 1.30 82 2.819 0.0164
## Multirotor - Ornithopter 1.14 1.34 82 0.853 0.6714
##
## Results are averaged over the levels of: Approach
## P value adjustment: tukey method for comparing a family of 3 estimates

#The fixed-wing was faster than the ornithopter,

# grouping all independent continuous factors into a single object
# to facilitate running the correlations
ind.cont <- binaryDt[c("AmbLight", "bTUVUground","Bgulls",
 "WindSpeedmps")]

# pairwise correlations between the independent continuous factors
corr.test(ind.cont, use = "pairwise", method = "pearson",
 adjust = "none")

## Call:corr.test(x = ind.cont, use = "pairwise", method = "pearson",
## adjust = "none")
## Correlation matrix
## AmbLight bTUVUground Bgulls WindSpeedmps
## AmbLight 1.00 -0.14 -0.09 -0.01
## bTUVUground -0.14 1.00 -0.13 -0.05
## Bgulls -0.09 -0.13 1.00 0.03
## WindSpeedmps -0.01 -0.05 0.03 1.00
## Sample Size
## [1] 94
## Probability values (Entries above the diagonal are adjusted for multiple tests.)
## AmbLight bTUVUground Bgulls WindSpeedmps
## AmbLight 0.00 0.17 0.38 0.92
## bTUVUground 0.17 0.00 0.21 0.62
## Bgulls 0.38 0.21 0.00 0.77
## WindSpeedmps 0.92 0.62 0.77 0.00
##
## To see confidence intervals of the correlations, print with the short=FALSE option

# you can make scatterplots of the association between all pairs
pairs(ind.cont, pch = 19, lower.panel = NULL)

# Create a binary model of focal vulture reaction (1) or not (0)
binaryGlmReaction <- glm(FocalReactionOrNot ~ Drone2*Approach+
 AmbLight+bTUVUground+Bgulls+WindSpeedmps,
 family = binomial(link="logit"),
 data=binaryDt)

binaryGlmReaction2 <- glm(FocalReactionOrNot ~ Drone2*Approach,
 family = binomial(link="logit"),
 data=binaryDt)

# Look at the results
Anova(binaryGlmReaction, type="2", test.statistic="LR", error.estimate="deviance")

## Analysis of Deviance Table (Type II tests)
##
## Response: FocalReactionOrNot
## LR Chisq Df Pr(>Chisq)
## Drone2 11.7002 2 0.00288 **
## Approach 23.6073 1 1.181e-06 ***
## AmbLight 0.8457 1 0.35778
## bTUVUground 2.2267 1 0.13564
## Bgulls 1.6315 1 0.20149
## WindSpeedmps 1.3676 1 0.24223
## Drone2:Approach 3.2557 2 0.19636
## ---
## Signif. codes: 0 '***' 0.001 '**' 0.01 '*' 0.05 '.' 0.1 ' ' 1

##There was an effect of drone platform and approach

# model results and report pseudoR2
with(binaryGlmReaction, null.deviance - deviance)

## [1] 43.52029

with(binaryGlmReaction, df.null - df.residual)

## [1] 9

with(binaryGlmReaction, pchisq(null.deviance - deviance,
 df.null - df.residual, lower.tail = FALSE))

## [1] 1.729713e-06

logLik(binaryGlmReaction)

## 'log Lik.' -40.29767 (df=10)

objects(binaryGlmReaction)

## [1] "aic" "boundary" "call"
## [4] "coefficients" "contrasts" "control"
## [7] "converged" "data" "deviance"
## [10] "df.null" "df.residual" "effects"
## [13] "family" "fitted.values" "formula"
## [16] "iter" "linear.predictors" "method"
## [19] "model" "null.deviance" "offset"
## [22] "prior.weights" "qr" "R"
## [25] "rank" "residuals" "terms"
## [28] "weights" "xlevels" "y"

pseudoR2 <- ((binaryGlmReaction$null.deviance-
 binaryGlmReaction$deviance)/binaryGlmReaction$null.deviance)
binaryGlmReaction$null.deviance

## [1] 124.1156

binaryGlmReaction$deviance

## [1] 80.59535

pseudoR2

## [1] 0.3506431

# emmeans and drone pairwise comparisons
# Call for response to obtain the results as probabilities
means.drone <- emmeans(binaryGlmReaction, "Drone2", type = "response")

## NOTE: Results may be misleading due to involvement in interactions

means.drone

## Drone2 prob SE df asymp.LCL asymp.UCL
## Fixed-wing 0.828 0.0838 Inf 0.603 0.939
## Multirotor 1.000 0.0862 Inf 0.000 1.000
## Ornithopter 0.384 0.1061 Inf 0.206 0.600
##
## Results are averaged over the levels of: Approach
## Confidence level used: 0.95
## Intervals are back-transformed from the logit scale

pairs(means.drone,reverse = TRUE)

## contrast odds.ratio SE df z.ratio p.value
## Multirotor / (Fixed-wing) 1.96e+03 1.61e+06 Inf 0.009 1.0000
## Ornithopter / (Fixed-wing) 1.29e-01 1.00e-01 Inf -2.728 0.0175
## Ornithopter / Multirotor 1.00e-04 1.00e-01 Inf -0.012 0.9999
##
## Results are averaged over the levels of: Approach
## P value adjustment: tukey method for comparing a family of 3 estimates
## Tests are performed on the log odds ratio scale

emDrones<-as.data.frame(means.drone)

# code for Figure 4(a) of the main text
ggplot(data=emDrones,aes(x=Drone2,y=prob))+
 geom_point(size=2)+ylim(0,1.5)+
 geom_errorbar(aes(ymin=prob-SE,ymax=prob+SE),width=0)+
 scale_color_brewer(palette = "Dark2") + guides(colour=FALSE) +
 theme_classic(base_size = 16)+xlab("sUAS")+
 ylab("Probability of reaction")+
 geom_point(data=binaryDt,aes(x=Drone2,y=FocalReactionOrNot),
 colour="grey",
 alpha=0.4,position=position_jitter(height=0,width=0.3))+
 theme_cowplot(font_size=11)

# emmeans and approach pairwise comparisons
# Call for response to obtain the results as probabilities
means.approach <- emmeans(binaryGlmReaction, "Approach",
 type = "response")

## NOTE: Results may be misleading due to involvement in interactions

means.approach

## Approach prob SE df asymp.LCL asymp.UCL
## Overhead 0.389 0.0799 Inf 0.248 0.552
## Targeted 0.999 0.3709 Inf 0.000 1.000
##
## Results are averaged over the levels of: Drone2
## Confidence level used: 0.95
## Intervals are back-transformed from the logit scale

emApproach<-as.data.frame(means.approach)

#Code for Figure 4(b) of the main text
ggplot(data=emApproach,aes(x=Approach,y=prob))+
 geom_point(size=2)+ylim(0,1.5)+geom_errorbar(aes(ymin=prob-SE,
 ymax=prob+SE),width=0)+scale_color_brewer(palette = "Dark2") +
 guides(colour=FALSE) + theme_classic(base_size = 16)+
 xlab("Approach")+ylab("Probability of reaction")+
 geom_point(data=binaryDt,aes(x=Approach,y=FocalReactionOrNot),
 colour="grey",
 alpha=0.4,position=position_jitter(height=0,width=0.3))+
 theme_cowplot(font_size=11)

################# Focal vulture reaction model########################
# filter to only include cases when reaction time was recorded
dtReaction <- dt[complete.cases(dt$ReactionTime),]
length(dtReaction$ReactionTime)

## [1] 59

# look at sample sizes (oh no 3!)
table(dtReaction$Drone2, dtReaction$Approach)

##
## Overhead Targeted
## Fixed-wing 10 16
## Multirotor 6 15
## Ornithopter 3 9

# remove ornithopter treatments
dtReaction2<-dtReaction[!(dtReaction$Treatment=="TO" |
 dtReaction$Treatment=="OO" ),]

# look at sample sizes
table(dtReaction2$Drone2, dtReaction2$Approach)

##
## Overhead Targeted
## Fixed-wing 10 16
## Multirotor 6 15

# build models with and without temporal auto-correlation
M0=gls(ReactionTime~Drone2*Approach+AmbLight+bTUVUground+Bgulls+
 WindSpeedmps,dtReaction2)
M0cor1=gls(ReactionTime~Drone2*Approach+AmbLight+bTUVUground+
 Bgulls+WindSpeedmps,dtReaction2,
 correlation=corCompSymm(form=~sort(ct)))

# rank models by AIC
AIC(M0, M0cor1)

## df AIC
## M0 9 387.6793
## M0cor1 10 389.6793

# plot response over time
ggplot(data=dtReaction2,aes(x=ct,y=ReactionTime))+
 geom_point()+theme_classic()+labs(x="Date",
 y="Focal TUVU reation time")

#code for Supplementary File S7 Figure 2

# auto-correlation plot
acf(residuals(M0, type="normalized"), main="Reaction Time",
 ylab="", xlab="")
mtext("Lag", 1, line= 2.5, cex=1.2)
mtext("Auto-correlation function", 2, line= 2.5, cex=1.2)

## No evidence of temporal auto-correlation

# descriptive statistics
Average <-ddply(dtReaction2, "Drone2", summarise,
 N=sum(!is.na(ReactionTime)),
 mean=mean(ReactionTime, na.rm=TRUE),
 sd = sd(ReactionTime, na.rm=TRUE),
 se = sd /sqrt (N))
Average

## Drone2 N mean sd se
## 1 Fixed-wing 26 36.78154 10.01873 1.964835
## 2 Multirotor 21 54.69381 23.75497 5.183759

Average <-ddply(dtReaction2, "Approach", summarise,
 N=sum(!is.na(ReactionTime)),
 mean=mean(ReactionTime, na.rm=TRUE),
 sd = sd(ReactionTime, na.rm=TRUE),
 se = sd /sqrt (N))
Average

## Approach N mean sd se
## 1 Overhead 16 56.80313 24.45407 6.113519
## 2 Targeted 31 38.58194 12.97286 2.329994

# check association between starting distance and the fixed effects
Anova(lm(StartingDistance ~ Drone2*Approach, dtReaction2), type="3")

## Anova Table (Type III tests)
##
## Response: StartingDistance
## Sum Sq Df F value Pr(>F)
## (Intercept) 2742127 1 1059.3419 < 2e-16 ***
## Drone2 17054 1 6.5885 0.01383 *
## Approach 2130 1 0.8229 0.36939
## Drone2:Approach 5572 1 2.1525 0.14962
## Residuals 111306 43
## ---
## Signif. codes: 0 '***' 0.001 '**' 0.01 '*' 0.05 '.' 0.1 ' ' 1

# starting distance is associated with drone, but not approach

# check association between drone speed and the fixed effects
Anova(lm(speed1~ Drone2*Approach, dtReaction2), type="3")

## Anova Table (Type III tests)
##
## Response: speed1
## Sum Sq Df F value Pr(>F)
## (Intercept) 10439.6 1 389.4785 < 2e-16 ***
## Drone2 95.7 1 3.5714 0.06587 .
## Approach 94.4 1 3.5221 0.06768 .
## Drone2:Approach 80.5 1 3.0027 0.09064 .
## Residuals 1099.0 41
## ---
## Signif. codes: 0 '***' 0.001 '**' 0.01 '*' 0.05 '.' 0.1 ' ' 1

# observed no association between drone speed and drone or approach

# include drone speed
dtReaction2 <- dtReaction2[complete.cases(dtReaction2$speed1),]

#Histogram plots
ggplot (dtReaction2, aes(x= bTUVUground)) + geom_histogram(binwidth=1)

ggplot (dtReaction2, aes(x= ReactionTime)) + geom_histogram(binwidth=10)

ggplot (dtReaction2, aes(x= Bgulls)) + geom_histogram(binwidth=100)

ggplot (dtReaction2, aes(x= WindSpeedmps)) + geom_histogram(binwidth=1)

ggplot (dtReaction2, aes(x= speed1)) + geom_histogram(binwidth=1)

ggplot (dtReaction2, aes(x= AmbLight)) + geom_histogram(binwidth=100)

# normal probability plots
qqnorm(dtReaction2$bTUVUground)
qqline(dtReaction2$bTUVUground)

qqnorm(dtReaction2$ReactionTime)
qqline(dtReaction2$ReactionTime)

qqnorm(dtReaction2$Bgulls)
qqline(dtReaction2$Bgulls)

qqnorm(dtReaction2$WindSpeedmps)
qqline(dtReaction2$WindSpeedmps)

qqnorm(dtReaction2$speed1)
qqline(dtReaction2$speed1)

qqnorm(dtReaction2$AmbLight)
qqline(dtReaction2$AmbLight)

# transform variables
dtReaction2 <- mutate(dtReaction2, log_TUVU= log(bTUVUground))
dtReaction2 <- mutate(dtReaction2, sqrt_TUVU = sqrt(bTUVUground))
dtReaction2 <- mutate(dtReaction2, log_ReactionTime= log(ReactionTime))
dtReaction2 <- mutate(dtReaction2, sqrt_ReactionTime = sqrt(ReactionTime))
dtReaction2 <- mutate(dtReaction2, log_Bgulls= log(Bgulls))
dtReaction2 <- mutate(dtReaction2, sqrt_Bgulls = sqrt(Bgulls))
dtReaction2 <- mutate(dtReaction2, log_WindSpeedmps= log(WindSpeedmps))
dtReaction2 <- mutate(dtReaction2, sqrt_WindSpeedmps = sqrt(WindSpeedmps))
dtReaction2 <- mutate(dtReaction2, log_speed1= log(speed1))
dtReaction2 <- mutate(dtReaction2, sqrt_speed1 = sqrt(speed1))
dtReaction2 <- mutate(dtReaction2, log_AmbLight= log(AmbLight))
dtReaction2 <- mutate(dtReaction2, sqrt_AmbLight = sqrt(AmbLight))

# Compare results
skewness(dtReaction2$bTUVUground)

## [1] 1.161258

skewness(dtReaction2$log_TUVU)

## [1] 0.1197932

skewness(dtReaction2$sqrt_TUVU)

## [1] 0.623088

kurtosis(dtReaction2$bTUVUground)

## [1] 3.586781

kurtosis(dtReaction2$log_TUVU)

## [1] 1.776134

kurtosis(dtReaction2$sqrt_TUVU)

## [1] 2.307089

skewness(dtReaction2$ReactionTime)

## [1] 1.531562

skewness(dtReaction2$log_ReactionTime)

## [1] 0.6406288

skewness(dtReaction2$sqrt_ReactionTime)

## [1] 1.082064

kurtosis(dtReaction2$ReactionTime)

## [1] 5.11553

kurtosis(dtReaction2$log_ReactionTime)

## [1] 2.924053

kurtosis(dtReaction2$sqrt_ReactionTime)

## [1] 3.772182

skewness(dtReaction2$Bgulls)

## [1] 1.311693

skewness(dtReaction2$log_Bgulls)

## [1] NaN

skewness(dtReaction2$sqrt_Bgulls)

## [1] -0.05024295

kurtosis(dtReaction2$Bgulls)

## [1] 5.120219

kurtosis(dtReaction2$log_Bgulls)

## [1] NaN

kurtosis(dtReaction2$sqrt_Bgulls)

## [1] 2.486439

skewness(dtReaction2$WindSpeedmps)

## [1] 0.1309375

skewness(dtReaction2$log_WindSpeedmps)

## [1] -0.7296601

skewness(dtReaction2$sqrt_WindSpeedmps)

## [1] -0.2922747

kurtosis(dtReaction2$WindSpeedmps)

## [1] 2.260602

kurtosis(dtReaction2$log_WindSpeedmps)

## [1] 2.715024

kurtosis(dtReaction2$sqrt_WindSpeedmps)

## [1] 2.297748

skewness(dtReaction2$speed1)

## [1] 0.7809009

skewness(dtReaction2$log_speed1)

## [1] -0.5800911

skewness(dtReaction2$sqrt_speed1)

## [1] 0.07074905

kurtosis(dtReaction2$speed1)

## [1] 4.844375

kurtosis(dtReaction2$log_speed1)

## [1] 3.644328

kurtosis(dtReaction2$sqrt_speed1)

## [1] 3.677238

skewness(dtReaction2$AmbLight)

## [1] 0.0600036

skewness(dtReaction2$log_AmbLight)

## [1] -0.5240241

skewness(dtReaction2$sqrt_AmbLight)

## [1] -0.2208452

kurtosis(dtReaction2$AmbLight)

## [1] 1.756667

kurtosis(dtReaction2$log_AmbLight)

## [1] 2.086471

kurtosis(dtReaction2$sqrt_AmbLight)

## [1] 1.827119

# Best combination of continuous variables are log_TUVU,
# sqrt_Bgulls, WindSpeed, sqrt_speed1.

ind.cont <- dtReaction2[c("sqrt_speed1","AmbLight","WindSpeedmps",
 "sqrt_Bgulls" ,"log_TUVU")]
corr.test(ind.cont, use = "pairwise", method = "pearson",
 adjust = "none")

## Call:corr.test(x = ind.cont, use = "pairwise", method = "pearson",
## adjust = "none")
## Correlation matrix
## sqrt_speed1 AmbLight WindSpeedmps sqrt_Bgulls log_TUVU
## sqrt_speed1 1.00 0.05 -0.32 -0.12 -0.03
## AmbLight 0.05 1.00 -0.16 -0.08 -0.21
## WindSpeedmps -0.32 -0.16 1.00 -0.06 0.05
## sqrt_Bgulls -0.12 -0.08 -0.06 1.00 -0.11
## log_TUVU -0.03 -0.21 0.05 -0.11 1.00
## Sample Size
## [1] 45
## Probability values (Entries above the diagonal are adjusted for multiple tests.)
## sqrt_speed1 AmbLight WindSpeedmps sqrt_Bgulls log_TUVU
## sqrt_speed1 0.00 0.73 0.03 0.45 0.83
## AmbLight 0.73 0.00 0.28 0.61 0.17
## WindSpeedmps 0.03 0.28 0.00 0.69 0.73
## sqrt_Bgulls 0.45 0.61 0.69 0.00 0.47
## log_TUVU 0.83 0.17 0.73 0.47 0.00
##
## To see confidence intervals of the correlations, print with the short=FALSE option

# you can make scatterplots of the association between all
# pairs of independent continuous factors much faster with
# the following trick
pairs(ind.cont, pch = 19, lower.panel = NULL)

# making sure that drone and approach are considered categorical factors
dtReaction2$Drone2 <- as.factor(dtReaction2$Drone2)
dtReaction2$Approach <- as.factor(dtReaction2$Approach)

# Build the reaction time model
ReactionModel<-lm(log_ReactionTime~Drone2*Approach+AmbLight+
 WindSpeedmps+log_TUVU+sqrt_Bgulls+sqrt_speed1,
 dtReaction2)

# Global validation of linear model assumptions
gvlma(ReactionModel)

##
## Call:
## lm(formula = log_ReactionTime ~ Drone2 * Approach + AmbLight +
## WindSpeedmps + log_TUVU + sqrt_Bgulls + sqrt_speed1, data = dtReaction2)
##
## Coefficients:
## (Intercept) Drone21 Approach1 AmbLight
## 3.876e+00 -1.972e-01 2.080e-01 -1.726e-05
## WindSpeedmps log_TUVU sqrt_Bgulls sqrt_speed1
## 5.089e-02 -2.082e-03 -4.070e-03 -1.663e-02
## Drone21:Approach1
## -1.232e-01
##
##
## ASSESSMENT OF THE LINEAR MODEL ASSUMPTIONS
## USING THE GLOBAL TEST ON 4 DEGREES-OF-FREEDOM:
## Level of Significance = 0.05
##
## Call:
## gvlma(x = ReactionModel)
##
## Value p-value Decision
## Global Stat 5.8400 0.21142 Assumptions acceptable.
## Skewness 0.5243 0.46901 Assumptions acceptable.
## Kurtosis 0.8857 0.34664 Assumptions acceptable.
## Link Function 1.3884 0.23868 Assumptions acceptable.
## Heteroscedasticity 3.0416 0.08115 Assumptions acceptable.

# Look at results
# Anova Table, type 3 sums of squares
Anova(ReactionModel, type="3",test.statistic="LR",
 error.estimate="deviance")

## Anova Table (Type III tests)
##
## Response: log_ReactionTime
## Sum Sq Df F value Pr(>F)
## (Intercept) 6.2146 1 79.2985 1.252e-10 ***
## Drone2 1.1737 1 14.9763 0.0004399 ***
## Approach 1.3385 1 17.0788 0.0002043 ***
## AmbLight 0.0034 1 0.0432 0.8364337
## WindSpeedmps 0.1132 1 1.4446 0.2372401
## log_TUVU 0.0001 1 0.0016 0.9686448
## sqrt_Bgulls 0.1655 1 2.1122 0.1547911
## sqrt_speed1 0.0039 1 0.0494 0.8254487
## Drone2:Approach 0.5135 1 6.5526 0.0148168 *
## Residuals 2.8213 36
## ---
## Signif. codes: 0 '***' 0.001 '**' 0.01 '*' 0.05 '.' 0.1 ' ' 1

# the interaction between drone and approach were significant
# the fixed effects drone and approach were significant

# calculate emmeans for drone
means.drone <- emmeans(ReactionModel, "Drone2")

## NOTE: Results may be misleading due to involvement in interactions

means.drone

## Drone2 emmean SE df lower.CL upper.CL
## Fixed-wing 3.62 0.0656 36 3.49 3.75
## Multirotor 4.01 0.0716 36 3.87 4.16
##
## Results are averaged over the levels of: Approach
## Confidence level used: 0.95

# calculate emmeans for approach
means.approach <- emmeans(ReactionModel, "Approach")

## NOTE: Results may be misleading due to involvement in interactions

means.approach

## Approach emmean SE df lower.CL upper.CL
## Overhead 4.02 0.0810 36 3.86 4.19
## Targeted 3.61 0.0523 36 3.50 3.71
##
## Results are averaged over the levels of: Drone2
## Confidence level used: 0.95

# emmeans interaction
int <- emmeans(ReactionModel, "Approach", by = c("Drone2"))
pairs (int)

## Drone2 = Fixed-wing:
## contrast estimate SE df t.ratio p.value
## Overhead - Targeted 0.170 0.141 36 1.198 0.2386
##
## Drone2 = Multirotor:
## contrast estimate SE df t.ratio p.value
## Overhead - Targeted 0.662 0.137 36 4.835 <.0001

means.int.df <- data.frame(int)

# code for Figure 5 of the main text
#Plot interaction
ggplot(data=means.int.df,aes(x=Drone2,y=emmean,group = Approach,
 colour = Approach))+
 geom_point(size=2)+geom_errorbar(aes(ymin=emmean-SE,ymax=emmean+SE),
 width=0)+scale_color_brewer(palette = "Dark2")+
 theme_classic(base_size = 16)+xlab("sUAS")+ylab("Log reaction time (sec)")+
 geom_point(data=dtReaction2,aes(x=Drone2,y=log_ReactionTime,
 group = Approach, colour = Approach),
 alpha=0.4,position=position_jitter(height=0,width=0.2))+
 theme_cowplot(font_size=11)

#################### Focal vulture FID model ###########################################
# Subset dataframe to include time when the focal bird reacted (n = 45)
FIDfocal<- dt[complete.cases(dt$FocalFID),]
length(FIDfocal$FocalFID)

## [1] 45

# look at sample sizes (oh no 3!)
table(FIDfocal$Drone2, FIDfocal$Approach)

##
## Overhead Targeted
## Fixed-wing 4 13
## Multirotor 3 13
## Ornithopter 3 9

# remove overhead treatments
FIDfocal2<-FIDfocal[!(FIDfocal$Treatment=="OO" |
 FIDfocal$Treatment=="OM"|
 FIDfocal$Treatment=="OF"),]

# look at sample sizes
table(FIDfocal2$Drone2, FIDfocal2$Approach)

##
## Targeted
## Fixed-wing 13
## Multirotor 13
## Ornithopter 9

# build models with and without temporal auto-correlation
M0=gls(FocalFID~Drone2+AmbLight+bTUVUground+WindSpeedmps+Bgulls,FIDfocal2)
M0cor1=gls(FocalFID~Drone2+AmbLight+bTUVUground+WindSpeedmps+
 Bgulls,FIDfocal2, correlation=corCompSymm(form=~sort(ct)))

# rank models by AIC
AIC(M0, M0cor1)

## df AIC
## M0 8 337.2277
## M0cor1 9 339.2277

# plot response over time
ggplot(data=FIDfocal2,aes(x=ct,y=FocalFID))+geom_point()+
 theme_classic()+labs(x="Date",y="Focal TUVU FID")

# Code for Supplementary File S7 Figure 3

# auto-correlation plot
acf(residuals(M0, type="normalized"), main="FID", ylab="", xlab="")
mtext("Lag", 1, line= 2.5, cex=1.2)
mtext("Auto-correlation function", 2, line= 2.5, cex=1.2)

#No evidence of temporal autocorrleation.

# descriptive statistics
Average <-ddply(FIDfocal2, "Drone2", summarise,
 N=sum(!is.na(FocalFID)),
 mean=mean(FocalFID, na.rm=TRUE),
 sd = sd(FocalFID, na.rm=TRUE),
 se = sd /sqrt (N))
Average

## Drone2 N mean sd se
## 1 Fixed-wing 13 65.64009 30.47076 8.451069
## 2 Multirotor 13 55.38088 25.40043 7.044813
## 3 Ornithopter 9 59.60797 44.19749 14.732498

#Drone speed versus drone and approach
Anova(lm(speed1~ Drone2, FIDfocal), type="3")

## Anova Table (Type III tests)
##
## Response: speed1
## Sum Sq Df F value Pr(>F)
## (Intercept) 9709.9 1 307.5869 < 2e-16 ***
## Drone2 257.4 2 4.0769 0.02409 *
## Residuals 1325.9 42
## ---
## Signif. codes: 0 '***' 0.001 '**' 0.01 '*' 0.05 '.' 0.1 ' ' 1

# speed is associated with drone

#Drone starting distance versus drone and approach
Anova(lm(StartingDistance ~ Drone2, FIDfocal), type="3")

## Anova Table (Type III tests)
##
## Response: StartingDistance
## Sum Sq Df F value Pr(>F)
## (Intercept) 2824051 1 909.6923 < 2e-16 ***
## Drone2 26869 2 4.3276 0.01955 *
## Residuals 130385 42
## ---
## Signif. codes: 0 '***' 0.001 '**' 0.01 '*' 0.05 '.' 0.1 ' ' 1

# SD is associated with drone

# Look at the relationship between starting distance and FID
ggplot(data=FIDfocal2, aes(x=StartingDistance,y=FocalFID))+
 geom_point()+theme_classic()+labs(x="Starting Distance",y="FID")

# No relationship between SD and FID.

# transform variables
FIDfocal2 <- mutate(FIDfocal2, log_TUVU= log(bTUVUground))
FIDfocal2 <- mutate(FIDfocal2, sqrt_TUVU = sqrt(bTUVUground))
FIDfocal2 <- mutate(FIDfocal2, log_FocalFID= log(FocalFID))
FIDfocal2 <- mutate(FIDfocal2, sqrt_FocalFID = sqrt(FocalFID))
FIDfocal2 <- mutate(FIDfocal2, log_Bgulls= log(Bgulls))
FIDfocal2 <- mutate(FIDfocal2, sqrt_Bgulls = sqrt(Bgulls))
FIDfocal2 <- mutate(FIDfocal2, log_AmbLight= log(AmbLight))
FIDfocal2 <- mutate(FIDfocal2, sqrt_AmbLight = sqrt(AmbLight))
FIDfocal2 <- mutate(FIDfocal2, log_WindSpeedmps= log(WindSpeedmps))
FIDfocal2<- mutate(FIDfocal2, sqrt_WindSpeedmps = sqrt(WindSpeedmps))

# Compare results
skewness(FIDfocal2$bTUVUground)

## [1] 1.329342

skewness(FIDfocal2$log_TUVU)

## [1] 0.1202267

skewness(FIDfocal2$sqrt_TUVU)

## [1] 0.6863357

kurtosis(FIDfocal2$bTUVUground)

## [1] 4.281634

kurtosis(FIDfocal2$log_TUVU)

## [1] 1.83692

kurtosis(FIDfocal2$sqrt_TUVU)

## [1] 2.57649

skewness(FIDfocal2$FocalFID)

## [1] 0.6831239

skewness(FIDfocal2$log_FocalFID)

## [1] -0.7309348

skewness(FIDfocal2$sqrt_FocalFID)

## [1] -0.03225889

kurtosis(FIDfocal2$FocalFID)

## [1] 3.369806

kurtosis(FIDfocal2$log_FocalFID)

## [1] 3.001534

kurtosis(FIDfocal2$sqrt_FocalFID)

## [1] 2.665762

skewness(FIDfocal2$Bgulls)

## [1] 0.9675235

skewness(FIDfocal2$log_Bgulls)

## [1] NaN

skewness(FIDfocal2$sqrt_Bgulls)

## [1] -0.1880366

kurtosis(FIDfocal2$Bgulls)

## [1] 4.221192

kurtosis(FIDfocal2$log_Bgulls)

## [1] NaN

kurtosis(FIDfocal2$sqrt_Bgulls)

## [1] 2.083809

skewness(FIDfocal2$AmbLight)

## [1] -0.1726623

skewness(FIDfocal2$log_AmbLight)

## [1] -0.8239588

skewness(FIDfocal2$sqrt_AmbLight)

## [1] -0.4944965

kurtosis(FIDfocal2$AmbLight)

## [1] 2.028842

kurtosis(FIDfocal2$log_AmbLight)

## [1] 2.647093

kurtosis(FIDfocal2$sqrt_AmbLight)

## [1] 2.245031

skewness(FIDfocal2$WindSpeedmps)

## [1] 0.2518474

skewness(FIDfocal2$log_WindSpeedmps)

## [1] -0.8765605

skewness(FIDfocal2$sqrt_WindSpeedmps)

## [1] -0.2993066

kurtosis(FIDfocal2$WindSpeedmps)

## [1] 2.802396

kurtosis(FIDfocal2$log_WindSpeedmps)

## [1] 3.483865

kurtosis(FIDfocal2$sqrt_WindSpeedmps)

## [1] 2.855399

# grouping all independent continuous factors into a single object
ind.cont <- FIDfocal2[c("log_TUVU", "sqrt_Bgulls","AmbLight",
 "WindSpeedmps")]
# runs the pairwise correlations
corr.test(ind.cont, use = "pairwise", method = "pearson",
 adjust = "none")

## Call:corr.test(x = ind.cont, use = "pairwise", method = "pearson",
## adjust = "none")
## Correlation matrix
## log_TUVU sqrt_Bgulls AmbLight WindSpeedmps
## log_TUVU 1.00 -0.20 -0.28 -0.04
## sqrt_Bgulls -0.20 1.00 -0.13 -0.19
## AmbLight -0.28 -0.13 1.00 -0.14
## WindSpeedmps -0.04 -0.19 -0.14 1.00
## Sample Size
## [1] 35
## Probability values (Entries above the diagonal are adjusted for multiple tests.)
## log_TUVU sqrt_Bgulls AmbLight WindSpeedmps
## log_TUVU 0.00 0.26 0.10 0.80
## sqrt_Bgulls 0.26 0.00 0.44 0.26
## AmbLight 0.10 0.44 0.00 0.44
## WindSpeedmps 0.80 0.26 0.44 0.00
##
## To see confidence intervals of the correlations, print with the short=FALSE option

pairs(ind.cont, pch = 19, lower.panel = NULL)

# We used the untransformed ambient light variable,
# log transformed TUVU group size, sqrt gull group size,
# and wind speed. These variables were not correlated
.

## function (..., .env = parent.frame())
## {
## structure(as.list(match.call()[-1]), env = .env, class = "quoted")
## }
## <bytecode: 0x000000004c520630>
## <environment: namespace:plyr>

# making sure that drone and approach are considered categorical factors
FIDfocal2$Drone2 <- as.factor(FIDfocal2$Drone2)

# Build the FID model
FIDtarget<-lm(sqrt_FocalFID~Drone2+AmbLight+log_TUVU+sqrt_Bgulls+
 WindSpeedmps,FIDfocal2)

# Global validation of linear model assumptions
gvlma(FIDtarget)

##
## Call:
## lm(formula = sqrt_FocalFID ~ Drone2 + AmbLight + log_TUVU + sqrt_Bgulls +
## WindSpeedmps, data = FIDfocal2)
##
## Coefficients:
## (Intercept) Drone21 Drone22 AmbLight log_TUVU
## 8.079e+00 -5.023e-02 6.351e-02 3.866e-06 -6.338e-01
## sqrt_Bgulls WindSpeedmps
## 4.174e-02 -4.046e-01
##
##
## ASSESSMENT OF THE LINEAR MODEL ASSUMPTIONS
## USING THE GLOBAL TEST ON 4 DEGREES-OF-FREEDOM:
## Level of Significance = 0.05
##
## Call:
## gvlma(x = FIDtarget)
##
## Value p-value Decision
## Global Stat 1.92389 0.7498 Assumptions acceptable.
## Skewness 0.28375 0.5943 Assumptions acceptable.
## Kurtosis 0.08327 0.7729 Assumptions acceptable.
## Link Function 0.57976 0.4464 Assumptions acceptable.
## Heteroscedasticity 0.97711 0.3229 Assumptions acceptable.

# Look at results
# Anova Table, type 3 sums of squares
Anova(FIDtarget, type="3",test.statistic="LR", error.estimate="deviance")

## Anova Table (Type III tests)
##
## Response: sqrt_FocalFID
## Sum Sq Df F value Pr(>F)
## (Intercept) 58.511 1 14.8089 0.0006303 ***
## Drone2 0.067 2 0.0085 0.9915136
## AmbLight 0.000 1 0.0000 0.9962515
## log_TUVU 7.840 1 1.9842 0.1699552
## sqrt_Bgulls 14.911 1 3.7739 0.0621777 .
## WindSpeedmps 5.411 1 1.3695 0.2517687
## Residuals 110.631 28
## ---
## Signif. codes: 0 '***' 0.001 '**' 0.01 '*' 0.05 '.' 0.1 ' ' 1

# No effect of drone. Gull group size NOT significant.

# calculate emmeans for drone
means.drone <- emmeans(FIDtarget, "Drone2")
means.drone

## Drone2 emmean SE df lower.CL upper.CL
## Fixed-wing 7.42 0.593 28 6.21 8.64
## Multirotor 7.54 0.596 28 6.32 8.76
## Ornithopter 7.46 0.697 28 6.03 8.89
##
## Confidence level used: 0.95

pairs(means.drone)

## contrast estimate SE df t.ratio p.value
## (Fixed-wing) - Multirotor -0.1137 0.885 28 -0.129 0.9909
## (Fixed-wing) - Ornithopter -0.0370 0.929 28 -0.040 0.9991
## Multirotor - Ornithopter 0.0768 0.937 28 0.082 0.9963
##
## P value adjustment: tukey method for comparing a family of 3 estimates

###############Vulture remaining index################################
# calculate the vulture remaining index
dt$remaining<-(dt$aTUVUground)/(dt$bTUVUground)
length(dt$remaining)

## [1] 100

# check sample sizes
table(dt$Drone2, dt$Approach)

##
## Overhead Targeted
## Fixed-wing 19 17
## Multirotor 16 16
## Ornithopter 16 16

# build models with and without temporal auto-correlation
M0=gls(remaining~Drone2*Approach+AmbLight+Bgulls+WindSpeedmps,dt)
M0cor1=gls(remaining~Drone2*Approach+AmbLight+Bgulls+WindSpeedmps,dt,
 correlation=corCompSymm(form=~sort(ct)))

# rank models by AIC
AIC(M0, M0cor1)

## df AIC
## M0 10 211.4208
## M0cor1 11 213.4208

# plot response over time
ggplot(data=dt,aes(x=ct,y=remaining))+geom_point()+theme_classic()+
 labs(x="Date",y="Remaining Vulture Index")

# Code for Supplementary File S7 Figure4

# auto-correlation plot
acf(residuals(M0, type="normalized"), main="Vulture remaining index",
 ylab="", xlab="")
mtext("Lag", 1, line= 2.5, cex=1.2)
mtext("Auto-correlation function", 2, line= 2.5, cex=1.2)

# No evidence of auto correlation.

# descriptive statistics
Average <-ddply(dt, "Drone2", summarise,
 N=sum(!is.na(remaining)),
 mean=mean(remaining, na.rm=TRUE),
 sd = sd(remaining, na.rm=TRUE),
 se = sd /sqrt (N))
Average

## Drone2 N mean sd se
## 1 Fixed-wing 36 0.4737634 0.5648640 0.09414400
## 2 Multirotor 32 0.3133681 0.5028906 0.08889934
## 3 Ornithopter 32 0.6572511 0.4998939 0.08836960

Average <-ddply(dt, "Approach", summarise,
 N=sum(!is.na(remaining)),
 mean=mean(remaining, na.rm=TRUE),
 sd = sd(remaining, na.rm=TRUE),
 se = sd /sqrt (N))
Average

## Approach N mean sd se
## 1 Overhead 51 0.6927602 0.5486678 0.07682886
## 2 Targeted 49 0.2609086 0.4314919 0.06164169

#Drone speed versus drone and approach
Anova(lm(speed1~ Drone2*Approach, dt), type="3")

## Anova Table (Type III tests)
##
## Response: speed1
## Sum Sq Df F value Pr(>F)
## (Intercept) 19327.3 1 788.2868 < 2e-16 ***
## Drone2 211.1 2 4.3043 0.01669 *
## Approach 26.0 1 1.0605 0.30613
## Drone2:Approach 96.5 2 1.9675 0.14635
## Residuals 2010.5 82
## ---
## Signif. codes: 0 '***' 0.001 '**' 0.01 '*' 0.05 '.' 0.1 ' ' 1

# speed is associated with drone, but not approach

#Drone starting distance versus drone and approach
Anova(lm(StartingDistance ~ Drone2*Approach, dt), type="3")

## Anova Table (Type III tests)
##
## Response: StartingDistance
## Sum Sq Df F value Pr(>F)
## (Intercept) 7176852 1 2115.2534 < 2e-16 ***
## Drone2 42273 2 6.2296 0.00288 **
## Approach 15 1 0.0043 0.94789
## Drone2:Approach 18097 2 2.6669 0.07472 .
## Residuals 318933 94
## ---
## Signif. codes: 0 '***' 0.001 '**' 0.01 '*' 0.05 '.' 0.1 ' ' 1

# starting distance is associated with drone, but not approach

# Transform variables
dt <- mutate(dt, log_AmbLight = log(AmbLight))
dt <- mutate(dt, sqrt_AmbLight = sqrt(AmbLight))
dt <- mutate(dt, log_TUVU= log(bTUVUground))
dt <- mutate(dt, sqrt_TUVU = sqrt(bTUVUground))
dt <- mutate(dt, log_Bgulls= log(Bgulls))
dt <- mutate(dt, sqrt_Bgulls = sqrt(Bgulls))
dt <- mutate(dt, log_Winds= log(WindSpeedmps))
dt <- mutate(dt, sqrt_Winds = sqrt(WindSpeedmps))

# Compare results
skewness(dt$AmbLight)

## [1] -0.1149118

skewness(dt$log_AmbLight)

## [1] -0.9392747

skewness(dt$sqrt_AmbLight)

## [1] -0.4707087

kurtosis(dt$AmbLight)

## [1] 1.881546

kurtosis(dt$log_AmbLight)

## [1] 3.293547

kurtosis(dt$sqrt_AmbLight)

## [1] 2.240756

skewness(dt$Bgulls)

## [1] 1.809496

skewness(dt$log_Bgulls)

## [1] NaN

skewness(dt$sqrt_Bgulls)

## [1] 0.250569

kurtosis(dt$Bgulls)

## [1] 7.463007

kurtosis(dt$log_Bgulls)

## [1] NaN

kurtosis(dt$sqrt_Bgulls)

## [1] 2.575882

skewness(dt$WindSpeedmps)

## [1] 0.1316395

skewness(dt$log_Winds)

## [1] -0.720575

skewness(dt$sqrt_Winds)

## [1] -0.2942911

kurtosis(dt$WindSpeedmps)

## [1] 2.24175

kurtosis(dt$log_Winds)

## [1] 2.565456

kurtosis(dt$sqrt_Winds)

## [1] 2.17049

# We used the untransformed ambient light variable,
# sqrt gull group size, and sqrt wind speed.

ind.cont <- dt[c("AmbLight", "sqrt_Bgulls","sqrt_Winds")]
corr.test(ind.cont, use = "pairwise", method = "pearson",
 adjust = "none")

## Call:corr.test(x = ind.cont, use = "pairwise", method = "pearson",
## adjust = "none")
## Correlation matrix
## AmbLight sqrt_Bgulls sqrt_Winds
## AmbLight 1.00 -0.08 -0.05
## sqrt_Bgulls -0.08 1.00 0.12
## sqrt_Winds -0.05 0.12 1.00
## Sample Size
## [1] 100
## Probability values (Entries above the diagonal are adjusted for multiple tests.)
## AmbLight sqrt_Bgulls sqrt_Winds
## AmbLight 0.00 0.45 0.59
## sqrt_Bgulls 0.45 0.00 0.24
## sqrt_Winds 0.59 0.24 0.00
##
## To see confidence intervals of the correlations, print with the short=FALSE option

pairs(ind.cont, pch = 19, lower.panel = NULL)

# These variables were not correlated.

# read fixed effects as factors
dt$Drone2<-as.factor(dt$Drone2)
dt$Approach<-as.factor(dt$Approach)

# Build the vulture remaining model
propDis<-lm(remaining~Drone2*Approach+AmbLight+sqrt_Bgulls+
 sqrt_Winds,dt)

# Global validation of linear model assumptions
gvlma(propDis)

##
## Call:
## lm(formula = remaining ~ Drone2 * Approach + AmbLight + sqrt_Bgulls +
## sqrt_Winds, data = dt)
##
## Coefficients:
## (Intercept) Drone21 Drone22 Approach1
## 5.837e-01 2.244e-03 -1.891e-01 2.087e-01
## AmbLight sqrt_Bgulls sqrt_Winds Drone21:Approach1
## -8.111e-05 -3.575e-03 5.209e-02 3.144e-02
## Drone22:Approach1
## 6.937e-02
##
##
## ASSESSMENT OF THE LINEAR MODEL ASSUMPTIONS
## USING THE GLOBAL TEST ON 4 DEGREES-OF-FREEDOM:
## Level of Significance = 0.05
##
## Call:
## gvlma(x = propDis)
##
## Value p-value Decision
## Global Stat 3.7203 0.4452 Assumptions acceptable.
## Skewness 0.9208 0.3373 Assumptions acceptable.
## Kurtosis 0.2318 0.6302 Assumptions acceptable.
## Link Function 2.3153 0.1281 Assumptions acceptable.
## Heteroscedasticity 0.2524 0.6154 Assumptions acceptable.

# Look at results
# Anova Table, type 3 sums of squares
Anova(propDis, type="3",test.statistic="LR", error.estimate="deviance")

## Anova Table (Type III tests)
##
## Response: remaining
## Sum Sq Df F value Pr(>F)
## (Intercept) 1.3138 1 5.6906 0.01913 *
## Drone2 2.1640 2 4.6867 0.01155 *
## Approach 4.2692 1 18.4923 4.278e-05 ***
## AmbLight 0.1762 1 0.7633 0.38461
## sqrt_Bgulls 0.3101 1 1.3431 0.24952
## sqrt_Winds 0.0378 1 0.1638 0.68665
## Drone2:Approach 0.5089 2 1.1023 0.33651
## Residuals 21.0087 91
## ---
## Signif. codes: 0 '***' 0.001 '**' 0.01 '*' 0.05 '.' 0.1 ' ' 1

# Drone and approach are significant

# emmeans for drone
means.drone <- emmeans(propDis, "Drone2")

## NOTE: Results may be misleading due to involvement in interactions

means.drone

## Drone2 emmean SE df lower.CL upper.CL
## Fixed-wing 0.479 0.0815 91 0.317 0.640
## Multirotor 0.287 0.0872 91 0.114 0.460
## Ornithopter 0.663 0.0853 91 0.494 0.832
##
## Results are averaged over the levels of: Approach
## Confidence level used: 0.95

pairs(means.drone)

## contrast estimate SE df t.ratio p.value
## (Fixed-wing) - Multirotor 0.191 0.122 91 1.574 0.2620
## (Fixed-wing) - Ornithopter -0.185 0.118 91 -1.570 0.2639
## Multirotor - Ornithopter -0.376 0.123 91 -3.061 0.0081
##
## Results are averaged over the levels of: Approach
## P value adjustment: tukey method for comparing a family of 3 estimates

emDrones<-as.data.frame(means.drone)

ggplot(data=emDrones,aes(x=Drone2,y=emmean))+
 geom_point(size=2)+ylim(0,1.5)+geom_errorbar(aes(ymin=emmean-SE,
 ymax=emmean+SE),width=0)+scale_color_brewer(palette = "Dark2") +
 guides(colour=FALSE) + theme_classic(base_size = 16)+xlab("sUAS")+
 ylab("Remaining index")+ geom_point(data=dt,aes(x=Drone2,y=remaining),
 colour="grey",alpha=0.4,position=position_jitter(height=0,width=0.3))+
 theme_cowplot(font_size=11)

## Warning: Removed 2 rows containing missing values (geom_point).

#emmeans for approach
means.approach <- emmeans(propDis, "Approach")

## NOTE: Results may be misleading due to involvement in interactions

means.approach

## Approach emmean SE df lower.CL upper.CL
## Overhead 0.685 0.0678 91 0.550 0.820
## Targeted 0.268 0.0689 91 0.131 0.404
##
## Results are averaged over the levels of: Drone2
## Confidence level used: 0.95

emApproach<-as.data.frame(means.approach)

ggplot(data=emApproach,aes(x=Approach,y=emmean))+
 geom_point(size=2)+ylim(0,1.5)+geom_errorbar(aes(ymin=emmean-SE,
 ymax=emmean+SE),width=0)+scale_color_brewer(palette = "Dark2") +
 guides(colour=FALSE) +theme_classic(base_size = 16)+xlab("Approach")+
 ylab("Remaining index")+geom_point(data=dt,aes(x=Approach,y=remaining),colour="grey",
 alpha=0.4,position=position_jitter(height=0,width=0.3))+
 theme_cowplot(font_size=11)

## Warning: Removed 2 rows containing missing values (geom_point).

################# Latency to return model ##################################

# All vultures had to disperse the study area to measure
# latency to return (n = 44).

# Subset dataframe
dtL<- dt[complete.cases(dt$LatencyMin),]
length(dtL$LatencyMin)

## [1] 44

# check sample sizes (oh no sample size of 2!)
table(dtL$Drone2, dtL$Approach)

##
## Overhead Targeted
## Fixed-wing 6 12
## Multirotor 5 13
## Ornithopter 2 6

# remove overhead treatments
dtL2<-dtL[!(dtL$Treatment=="OO" | dtL$Treatment=="OM"|
 dtL$Treatment=="OF"),]

# check sample sizes
table(dtL2$Drone, dtL2$Approach.ab)

##
## T
## F 12
## M 13
## O 6

# descriptive statistics
Average <-ddply(dtL2, "Drone2", summarise,
 N=sum(!is.na(LatencyMin)),
 mean=mean(LatencyMin, na.rm=TRUE),
 sd = sd(LatencyMin, na.rm=TRUE),
 se = sd /sqrt (N))
Average

## Drone2 N mean sd se
## 1 Fixed-wing 12 27.41667 34.77057 10.037399
## 2 Multirotor 13 15.46154 13.83604 3.837426
## 3 Ornithopter 6 27.83333 27.33069 11.157708

# build models with and without temporal auto-correlation
M0=gls(LatencyMin~Drone2+AmbLight+bTUVUground+WindSpeedmps+Bgulls,dtL2)
M0cor1=gls(LatencyMin~Drone2+AmbLight+bTUVUground+WindSpeedmps+Bgulls,
 dtL2, correlation=corCompSymm(form=~sort(ct)))

# rank models by AIC
AIC(M0, M0cor1)

## df AIC
## M0 8 294.0337
## M0cor1 9 296.0337

# plot response over time
ggplot(data=dtL,aes(x=ct,y=LatencyMin))+geom_point()+theme_classic()+
 labs(x="Date",y="Latency to return")

# Code for Supplementary File S7 Figure 5

# auto-correlation plot
acf(residuals(M0, type="normalized"), main="Reaction Time", ylab="",
 xlab="")
mtext("Lag", 1, line= 2.5, cex=1.2)
mtext("Auto-correlation function", 2, line= 2.5, cex=1.2)

#No evidence for temporal auto-correlation
# Drone speed versus drone and approach
Anova(lm(speed1~ Drone2, dtL2), type="3")

## Anova Table (Type III tests)
##
## Response: speed1
## Sum Sq Df F value Pr(>F)
## (Intercept) 5342.1 1 188.7717 5.735e-14 ***
## Drone2 13.4 2 0.2376 0.7901
## Residuals 792.4 28
## ---
## Signif. codes: 0 '***' 0.001 '**' 0.01 '*' 0.05 '.' 0.1 ' ' 1

# speed is not associated with drone

#Drone starting distance versus drone and approach
Anova(lm(StartingDistance ~ Drone2, dtL2), type="3")

## Anova Table (Type III tests)
##
## Response: StartingDistance
## Sum Sq Df F value Pr(>F)
## (Intercept) 1841676 1 560.4583 < 2e-16 ***
## Drone2 34374 2 5.2303 0.01175 *
## Residuals 92009 28
## ---
## Signif. codes: 0 '***' 0.001 '**' 0.01 '*' 0.05 '.' 0.1 ' ' 1

# Starting distance is associated with drone

# Transform variables
dtL2 <- mutate(dtL2, log_AmbLight = log(AmbLight))
dtL2 <- mutate(dtL2, sqrt_AmbLight = sqrt(AmbLight))
dtL2 <- mutate(dtL2, log_TUVU= log(bTUVUground))
dtL2 <- mutate(dtL2, sqrt_TUVU = sqrt(bTUVUground))
dtL2 <- mutate(dtL2, log_LatencyMin= log(LatencyMin))
dtL2 <- mutate(dtL2, sqrt_LatencyMin = sqrt(LatencyMin))
dtL2 <- mutate(dtL2, log_Bgulls= log(Bgulls))
dtL2 <- mutate(dtL2, sqrt_Bgulls = sqrt(Bgulls))
dtL2 <- mutate(dtL2, log_WindSpeed= log(WindSpeedmps))
dtL2 <- mutate(dtL2, sqrt_WindSpeed = sqrt(WindSpeedmps))
dtL2 <- mutate(dtL2, log_speed= log(speed1))
dtL2 <- mutate(dtL2, sqrt_speed = sqrt(speed1))

# Compare results
skewness(dtL2$AmbLight)

## [1] 0.06597292

skewness(dtL2$log_AmbLight)

## [1] -0.4416599

skewness(dtL2$sqrt_AmbLight)

## [1] -0.1757506

kurtosis(dtL2$AmbLight)

## [1] 1.659598

kurtosis(dtL2$log_AmbLight)

## [1] 1.945498

kurtosis(dtL2$sqrt_AmbLight)

## [1] 1.734415

skewness(dtL2$bTUVUground)

## [1] 1.026301

skewness(dtL2$log_TUVU)

## [1] 0.1602657

skewness(dtL2$sqrt_TUVU)

## [1] 0.6082568

kurtosis(dtL2$bTUVUground)

## [1] 2.866751

kurtosis(dtL2$log_TUVU)

## [1] 1.79062

kurtosis(dtL2$sqrt_TUVU)

## [1] 2.17414

skewness(dtL2$LatencyMin)

## [1] 2.009003

skewness(dtL2$log_LatencyMin)

## [1] 0.07910235

skewness(dtL2$sqrt_LatencyMin)

## [1] 1.009234

kurtosis(dtL2$LatencyMin)

## [1] 7.388929

kurtosis(dtL2$log_LatencyMin)

## [1] 2.138967

kurtosis(dtL2$sqrt_LatencyMin)

## [1] 3.396637

skewness(dtL2$Bgulls)

## [1] 1.15614

skewness(dtL2$log_Bgulls)

## [1] NaN

skewness(dtL2$sqrt_Bgulls)

## [1] 0.04608253

kurtosis(dtL2$Bgulls)

## [1] 4.379909

kurtosis(dtL2$log_Bgulls)

## [1] NaN

kurtosis(dtL2$sqrt_Bgulls)

## [1] 1.917154

skewness(dtL2$WindSpeedmps)

## [1] 0.2273432

skewness(dtL2$log_WindSpeed)

## [1] -0.9216843

skewness(dtL2$sqrt_WindSpeed)

## [1] -0.3185507

kurtosis(dtL2$WindSpeedmps)

## [1] 2.839549

kurtosis(dtL2$log_WindSpeed)

## [1] 3.859623

kurtosis(dtL2$sqrt_WindSpeed)

## [1] 3.01487

skewness(dtL2$speed1)

## [1] 0.3763195

skewness(dtL2$log_speed)

## [1] -0.6359887

skewness(dtL2$sqrt_speed)

## [1] -0.1321431

kurtosis(dtL2$speed1)

## [1] 3.088082

kurtosis(dtL2$log_speed)

## [1] 3.084842

kurtosis(dtL2$sqrt_speed)

## [1] 2.784817

# Best combination of continuous variables are AmbLight,
# sqrt_Bgulls, log_TUVU, and log_LatencyMin, and windspeed.

ind.cont <- dtL2[c("AmbLight","WindSpeedmps","sqrt_Bgulls" ,
 "log_TUVU","sqrt_speed")]
corr.test(ind.cont, use = "pairwise", method = "pearson",
 adjust = "none")

## Call:corr.test(x = ind.cont, use = "pairwise", method = "pearson",
## adjust = "none")
## Correlation matrix
## AmbLight WindSpeedmps sqrt_Bgulls log_TUVU sqrt_speed
## AmbLight 1.00 0.00 -0.07 -0.40 -0.10
## WindSpeedmps 0.00 1.00 -0.07 -0.04 -0.26
## sqrt_Bgulls -0.07 -0.07 1.00 -0.14 -0.25
## log_TUVU -0.40 -0.04 -0.14 1.00 0.17
## sqrt_speed -0.10 -0.26 -0.25 0.17 1.00
## Sample Size
## [1] 31
## Probability values (Entries above the diagonal are adjusted for multiple tests.)
## AmbLight WindSpeedmps sqrt_Bgulls log_TUVU sqrt_speed
## AmbLight 0.00 1.00 0.70 0.03 0.58
## WindSpeedmps 1.00 0.00 0.72 0.81 0.17
## sqrt_Bgulls 0.70 0.72 0.00 0.47 0.17
## log_TUVU 0.03 0.81 0.47 0.00 0.35
## sqrt_speed 0.58 0.17 0.17 0.35 0.00
##
## To see confidence intervals of the correlations, print with the short=FALSE option

pairs(ind.cont, pch = 19, lower.panel = NULL)

# Read fixed effects as factors
dtL2$Drone2<-as.factor(dtL2$Drone2)

# Build the latency model
LateLmT<-lm(log_LatencyMin~Drone2+AmbLight+log_TUVU+WindSpeedmps+
 sqrt_Bgulls+sqrt_speed,dtL2)

# Global validation of linear model assumptions
gvlma(LateLmT)

##
## Call:
## lm(formula = log_LatencyMin ~ Drone2 + AmbLight + log_TUVU +
## WindSpeedmps + sqrt_Bgulls + sqrt_speed, data = dtL2)
##
## Coefficients:
## (Intercept) Drone21 Drone22 AmbLight log_TUVU
## 6.122e+00 -3.654e-02 7.280e-02 1.543e-05 -2.586e-01
## WindSpeedmps sqrt_Bgulls sqrt_speed
## -1.906e-01 -3.880e-03 -7.341e-01
##
##
## ASSESSMENT OF THE LINEAR MODEL ASSUMPTIONS
## USING THE GLOBAL TEST ON 4 DEGREES-OF-FREEDOM:
## Level of Significance = 0.05
##
## Call:
## gvlma(x = LateLmT)
##
## Value p-value Decision
## Global Stat 0.93550 0.9194 Assumptions acceptable.
## Skewness 0.02463 0.8753 Assumptions acceptable.
## Kurtosis 0.84097 0.3591 Assumptions acceptable.
## Link Function 0.03197 0.8581 Assumptions acceptable.
## Heteroscedasticity 0.03792 0.8456 Assumptions acceptable.

# Look at results
# Anova Table, type 3 sums of squares
Anova(LateLmT, type="3",test.statistic="LR", error.estimate="deviance")

## Anova Table (Type III tests)
##
## Response: log_LatencyMin
## Sum Sq Df F value Pr(>F)
## (Intercept) 14.8768 1 11.5141 0.002499 **
## Drone2 0.0650 2 0.0251 0.975202
## AmbLight 0.0015 1 0.0012 0.973215
## log_TUVU 0.9976 1 0.7721 0.388654
## WindSpeedmps 0.9402 1 0.7277 0.402429
## sqrt_Bgulls 0.1358 1 0.1051 0.748743
## sqrt_speed 6.5516 1 5.0707 0.034184 *
## Residuals 29.7171 23
## ---
## Signif. codes: 0 '***' 0.001 '**' 0.01 '*' 0.05 '.' 0.1 ' ' 1

# We did not see any drone, approach, ambient light, or
# flock size effects on latency for a vulture to return.
# Speed was significant.

means.drone <- emmeans(LateLmT, "Drone2")
means.drone

## Drone2 emmean SE df lower.CL upper.CL
## Fixed-wing 2.48 0.354 23 1.75 3.21
## Multirotor 2.59 0.349 23 1.87 3.31
## Ornithopter 2.48 0.494 23 1.46 3.50
##
## Confidence level used: 0.95

pairs(means.drone)

## contrast estimate SE df t.ratio p.value
## (Fixed-wing) - Multirotor -0.109335 0.530 23 -0.206 0.9768
## (Fixed-wing) - Ornithopter -0.000274 0.606 23 0.000 1.0000
## Multirotor - Ornithopter 0.109061 0.628 23 0.174 0.9835
##
## P value adjustment: tukey method for comparing a family of 3 estimates

ggplot(data=dtL2, aes(x=sqrt_speed,y=log_LatencyMin))+
 geom_point()+theme_classic()+
 labs(x="Square root transformed UAS speed",y="Log transformed latency")

# code for Supplementary File S7 Figure 5

# this is predicted line comparing only chosen variables
ggplot(data = dtL2, aes(x = sqrt_speed, y = log_LatencyMin)) +
 geom_point(color='black') +
 geom_smooth(method = "lm", se = FALSE, color='black')+
 labs(x="Square root transformed UAS speed",y="Log latency to return")+
 theme_cowplot(font_size=11)

## `geom_smooth()` using formula 'y ~ x'

################ Number of passes #############################
#Number of passes versus drone and approach
passes <- dt[complete.cases(dt$NoOfPasses),]
unique(passes$NoOfPasses)

## [1] "1" "TBD" "2" "6" "5"
## [6] "3" "at least 2" "4"

passes<- subset.data.frame(passes, NoOfPasses == 1 | NoOfPasses == 2 |
 NoOfPasses == 3|NoOfPasses == 4|
 NoOfPasses == 5|NoOfPasses == 6)

# check sample sizes (oh no sample size of 0!)
table(passes$Drone2, passes$Approach)

##
## Overhead Targeted
## Fixed-wing 3 17
## Multirotor 0 15
## Ornithopter 1 14

# remove overhead treatments
passes2<-passes[!(passes$Treatment=="OO" | passes$Treatment=="OM"|
 passes$Treatment=="OF"),]

length(passes2$NoOfPasses)

## [1] 46

passes2$NoOfPasses<-as.integer(passes2$NoOfPasses)
passes2$Drone2<-as.factor(passes2$Drone2)
Anova(lm(NoOfPasses ~ Drone2, passes2), type="3")

## Anova Table (Type III tests)
##
## Response: NoOfPasses
## Sum Sq Df F value Pr(>F)
## (Intercept) 155.673 1 110.9093 1.752e-13 ***
## Drone2 11.580 2 4.1249 0.02297 *
## Residuals 60.355 43
## ---
## Signif. codes: 0 '***' 0.001 '**' 0.01 '*' 0.05 '.' 0.1 ' ' 1

#Create linear model and run ANOVA
passes.lm <- lm(NoOfPasses ~ Drone2, passes2)
means.passes <- emmeans(passes.lm, "Drone2")
means.passes

## Drone2 emmean SE df lower.CL upper.CL
## Fixed-wing 2.12 0.287 43 1.538 2.70
## Multirotor 1.13 0.306 43 0.516 1.75
## Ornithopter 2.29 0.317 43 1.647 2.92
##
## Confidence level used: 0.95

pairs(means.passes)

## contrast estimate SE df t.ratio p.value
## (Fixed-wing) - Multirotor 0.984 0.420 43 2.345 0.0602
## (Fixed-wing) - Ornithopter -0.168 0.428 43 -0.393 0.9185
## Multirotor - Ornithopter -1.152 0.440 43 -2.617 0.0320
##
## P value adjustment: tukey method for comparing a family of 3 estimates

means.passes <- as.data.frame(means.passes)

#Plot interaction
ggplot(data=means.passes,aes(x=Drone2,y=emmean))+
 geom_point(size=2)+geom_errorbar(aes(ymin=emmean-SE,ymax=emmean+SE),
 width=0)+scale_color_brewer(palette = "Dark2")+
 theme_classic(base_size = 16)+xlab("sUAS")+ylab("Number of passes")+
 geom_point(data=passes2,aes(x=Drone2,y=NoOfPasses),
 alpha=0.4,position=position_jitter(height=0,width=0.2))+
 theme_cowplot(font_size=11)

# Number of passes was calculated for targeted approaches.

# **Supplementary File S9.** Covariate model summaries

**Supplementary File S9 Table 1.** Associations of our fixed and independent effects with UAS speed and starting distance for all our models. Significant results are in bold.

|  |  | *F* | df | *P* |
| --- | --- | --- | --- | --- |
| **Binomial focal vulture reaction (*n* = 94)** |  |  |  |  |
| UAS speed (m s^-1^) | Approach | 1.06 | 1, 82 | 0.31 |
|  | UAS platform *approach | 1.97 | 2, 82 | 0.15 |
| Starting distance (m) | Approach | 0.08 | 1, 88 | 0.78 |
|  | UAS platform *approach | 1.80 | 2, 88 | 0.17 |
| **Focal vulture reaction time (*n* = 47)** |  |  |  |  |
| UAS speed (m s^-1^) | UAS platform | 3.57 | 1, 41 | 0.07 |
|  | Approach | 3.52 | 1, 41 | 0.07 |
|  | UAS platform *approach | 3.00 | 1, 41 | 0.09 |
| Starting distance (m) | UAS platform | 6.59 | 1, 43 | **0.01** |
|  | Approach | 0.83 | 1, 43 | 0.37 |
|  | UAS platform * approach | 2.15 | 1, 43 | 0.15 |
| **Focal vulture FID (*n* = 35)** |  |  |  |  |
| UAS speed (m s^-1^) | UAS platform | 4.08 | 2, 42 | **0.02** |
| Starting distance (m) | UAS platform | 4.33 | 2, 42 | **0.02** |
| **Vulture remaining index (*n* = 100)** |  |  |  |  |
| UAS speed (m s^-1^) | UAS platform | 4.30 | 2, 82 | **0.02** |
|  | Approach | 1.06 | 1, 82 | 0.31 |
|  | UAS platform * approach | 1.97 | 2, 82 | 0.15 |
| Starting distance (m) | UAS platform | 6.23 | 2, 94 | **0.00** |
|  | Approach | 0.00 | 1, 94 | 0.95 |
|  | UAS platform * approach | 2.67 | 2, 94 | 0.07 |
| **Latency to return (*n* = 31)** |  |  |  |  |
| UAS speed (m s^-1^) | UAS platform | 0.24 | 2, 28 | 0.79 |
| Starting distance (m) | UAS platform | 5.23 | 2, 28 | **0.01** |

# **Supplementary File S10.** Metadata for georeferenced photographs

**Supplementary File S10 Table 1.** Root Mean Square (RMS), transformation, and number of control points for georeferenced photographs used to calculate FID.

| Photo name | RMS error | Transformation | Number of Control Points |
| --- | --- | --- | --- |
| Run4.FID4 | 0.000549 | Spline | 20 |
| Run6.FID1 | 0.000318 | Spline | 21 |
| Run4.Photo4 | 0.000159 | Spline | 20 |
| Run4.FID2 | 0.000156 | Spline | 29 |
| Run4.Photo3 | 0.000138 | Spline | 30 |
| Run6_Photo1 | 0.000104 | Spline | 17 |
| Run42.Photo2 | 0.000073 | Spline | 23 |
| Run87.Photo3 | 0.000062 | Spline | 36 |
| Run21.FID1 | 0.000061 | Spline | 17 |
| Run43.Photo1 | 0.00006 | Spline | 26 |
| Run60.Photo1 | 0.00006 | Spline | 20 |
| Run4.FID3 | 0.000059 | Spline | 30 |
| Run87.Photo4 | 0.000058 | Spline | 34 |
| Run4.FID5 | 0.000057 | Spline | 29 |
| Run13.FID1 | 0.000054 | Spline | 29 |
| Run13.Photo2 | 0.000054 | Spline | 28 |
| Run16.Photo2 | 0.000052 | Spline | 29 |
| Run16.Photo1 | 0.00005 | Spline | 21 |
| Run44.FID4 | 0.000045 | Spline | 25 |
| Run4.FID7 | 0.00004 | Spline | 27 |
| Run21.FID3_4 | 0.000038 | Spline | 17 |
| Run22.FID1 | 0.000038 | Spline | 16 |
| Run13.FID2 | 0.000037 | Spline | 40 |
| Run96.FID7 | 0.000036 | Spline | 49 |
| Run13.FID11 | 0.000035 | Spline | 30 |
| Run1.Photo1 | 0.000034 | Spline | 32 |
| Run2.Photo2 | 0.000034 | Spline | 25 |
| Run27.Photo1 | 0.000034 | Spline | 25 |
| Run45.FID3 | 0.000033 | Spline | 19 |
| Run28.FID7 | 0.000029 | Spline | 22 |
| Run22.FID14 | 0.000028 | Spline | 24 |
| Run6.FID3 | 0.000027 | Spline | 33 |
| Run13.FID13 | 0.000027 | Spline | 35 |
| Run4.FID6 | 0.000026 | Spline | 20 |
| Run4.FID8 | 0.000026 | Spline | 20 |
| Run13.FID6 | 0.000026 | Spline | 33 |
| Run41.Photo2 | 0.000026 | Spline | 24 |
| Run13.Photo3 | 0.000025 | Spline | 27 |
| Run13.FID12 | 0.000024 | Spline | 29 |
| Run48.Photo1 | 0.00002 | Spline | 28 |
| Run5.Photo1 | 0.000019 | Spline | 13 |
| Run10.FID1 | 0.000019 | Spline | 27 |
| Run22.FID10 | 0.000019 | Spline | 27 |
| Run84.FID5 | 0.000019 | Spline | 21 |
| Run96.FID5 | 0.000019 | Spline | 29 |
| Run88.Photo1 | 0.000018 | Spline | 20 |
| Run22.FID2_3_4 | 0.000017 | Spline | 30 |
| Run24.Photo2 | 0.000017 | Spline | 39 |
| Run30.Photo2 | 0.000017 | Spline | 28 |
| Run37.Photo4 | 0.000017 | Spline | 20 |
| Run37.Photo8 | 0.000017 | Spline | 24 |
| Run48.FID4 | 0.000017 | Spline | 25 |
| Run96.Photo8 | 0.000017 | Spline | 31 |
| Run26.Photo1 | 0.000016 | Spline | 19 |
| Run42.Photo1 | 0.000016 | Spline | 27 |
| Run96.Photo4 | 0.000016 | Spline | 25 |
| Run13.FID16 | 0.000015 | Spline | 38 |
| Run8.Photo2 | 0.000014 | Spline | 20 |
| Run13.FID14 | 0.000014 | Spline | 34 |
| Run19.Photo2 | 0.000014 | Spline | 27 |
| Run22.FID9 | 0.000014 | Spline | 34 |
| Run34.Photo2 | 0.000014 | Spline | 17 |
| Run48.FID1 | 0.000014 | Spline | 24 |
| Run49.Photo2 | 0.000014 | Spline | 36 |
| Run67.Photo1 | 0.000014 | Spline | 30 |
| Run74.Photo1 | 0.000014 | Spline | 36 |
| Run85.Photo2 | 0.000014 | Spline | 17 |
| Run2.Photo1 | 0.000013 | Spline | 10 |
| Run4.FID10 | 0.000013 | Spline | 15 |
| Run19.Photo1 | 0.000013 | Spline | 26 |
| Run22.Photo3 | 0.000013 | Spline | 22 |
| Run47.Photo1 | 0.000013 | Spline | 18 |
| Run75.FID2 | 0.000013 | Spline | 20 |
| Run21.Photo1 | 0.000012 | Spline | 27 |
| Run24.Photo1 | 0.000012 | Spline | 21 |
| Run34.Photo1 | 0.000012 | Spline | 32 |
| Run48.FID3 | 0.000012 | Spline | 18 |
| Run57.FID6 | 0.000012 | Spline | 19 |
| Run4.FID1 | 0.000011 | Spline | 18 |
| Run11.Photo1 | 0.000011 | Spline | 33 |
| Run12.Photo2 | 0.000011 | Spline | 33 |
| Run13.FID9 | 0.000011 | Spline | 32 |
| Run22.FID5 | 0.000011 | Spline | 26 |
| Run26.Photo2 | 0.000011 | Spline | 21 |
| Run27.Photo2 | 0.000011 | Spline | 20 |
| Run38.Photo2 | 0.000011 | Spline | 22 |
| Run52.Photo1 | 0.000011 | Spline | 25 |
| Run58.Photo1 | 0.000011 | Spline | 30 |
| Run69.Photo1 | 0.000011 | Spline | 27 |
| Run75.FID3 | 0.000011 | Spline | 24 |
| Run99.Photo2 | 0.000011 | Spline | 24 |
| Run7.FID1 | 0.00001 | Spline | 19 |
| Run14.FID4 | 0.00001 | Spline | 26 |
| Run20.Photo1 | 0.00001 | Spline | 21 |
| Run22.FID6_7_8 | 0.00001 | Spline | 27 |
| Run28.FID2_3 | 0.00001 | Spline | 18 |
| Run33.FID2 | 0.00001 | Spline | 28 |
| Run43.FID2 | 0.00001 | Spline | 24 |
| Run54.Photo2 | 0.00001 | Spline | 21 |
| Run57.FID4 | 0.00001 | Spline | 18 |
| Run57.Photo2 | 0.00001 | Spline | 28 |
| Run59.FID1 | 0.00001 | Spline | 27 |
| Run59.Photo1 | 0.00001 | Spline | 26 |
| Run65.FID3 | 0.00001 | Spline | 32 |
| Run88.FID3 | 0.00001 | Spline | 28 |
| Run96.Photo7 | 0.00001 | Spline | 19 |
| Run10.Photo2 | 0.000009 | Spline | 29 |
| Run13.FID8 | 0.000009 | Spline | 35 |
| Run14.FID1 | 0.000009 | Spline | 32 |
| Run29.Photo2 | 0.000009 | Spline | 30 |
| Run37.FID10 | 0.000009 | Spline | 21 |
| Run41.FID2 | 0.000009 | Spline | 25 |
| Run43.FID1 | 0.000009 | Spline | 20 |
| Run45.FID1 | 0.000009 | Spline | 39 |
| Run46.Photo1 | 0.000009 | Spline | 19 |
| Run48.FID6 | 0.000009 | Spline | 22 |
| Run56.Photo2 | 0.000009 | Spline | 28 |
| Run61.FID2 | 0.000009 | Spline | 26 |
| Run73.Photo2 | 0.000009 | Spline | 29 |
| Run74.Photo2 | 0.000009 | Spline | 40 |
| Run77.Photo2 | 0.000009 | Spline | 28 |
| Run87.Photo1 | 0.000009 | Spline | 22 |
| Run90.Photo1 | 0.000009 | Spline | 23 |
| Run96.Photo1 | 0.000009 | Spline | 23 |
| Run96.Photo5 | 0.000009 | Spline | 22 |
| Run12.Photo1 | 0.000008 | Spline | 21 |
| Run13.FID10 | 0.000008 | Spline | 30 |
| Run13.Photo1 | 0.000008 | Spline | 35 |
| Run22.Photo4 | 0.000008 | Spline | 23 |
| Run43.Photo2 | 0.000008 | Spline | 20 |
| Run55.Photo2 | 0.000008 | Spline | 21 |
| Run60.FID1 | 0.000008 | Spline | 21 |
| Run77.Photo1 | 0.000008 | Spline | 40 |
| Run79.FID3 | 0.000008 | Spline | 21 |
| Run81.Photo2 | 0.000008 | Spline | 16 |
| Run90.FID5_6 | 0.000008 | Spline | 22 |
| Run1.Photo2 | 0.000007 | Spline | 30 |
| Run4.FID9 | 0.000007 | Spline | 19 |
| Run6.FID2 | 0.000007 | Spline | 17 |
| Run7.FID4_FID5 | 0.000007 | Spline | 22 |
| Run7.Photo2 | 0.000007 | Spline | 20 |
| Run28.FID4 | 0.000007 | Spline | 24 |
| Run33.Photo1 | 0.000007 | Spline | 17 |
| Run37.FID4 | 0.000007 | Spline | 18 |
| Run37.Photo3 | 0.000007 | Spline | 22 |
| Run42.FID1 | 0.000007 | Spline | 23 |
| Run45.FID6_7 | 0.000007 | Spline | 33 |
| Run47.FID1 | 0.000007 | Spline | 21 |
| Run47.Photo2 | 0.000007 | Spline | 20 |
| Run53.Photo2 | 0.000007 | Spline | 17 |
| Run65.Photo1 | 0.000007 | Spline | 23 |
| Run79.FID1 | 0.000007 | Spline | 20 |
| Run80.FID1 | 0.000007 | Spline | 17 |
| Run82.FID1 | 0.000007 | Spline | 26 |
| Run83.Photo1 | 0.000007 | Spline | 18 |
| Run84.Photo4 | 0.000007 | Spline | 19 |
| Run86.Photo1 | 0.000007 | Spline | 21 |
| Run88.FID1 | 0.000007 | Spline | 27 |
| Run89.Photo2 | 0.000007 | Spline | 22 |
| Run96.FID4 | 0.000007 | Spline | 29 |
| Run96.Photo2 | 0.000007 | Spline | 27 |
| Run13.FID17 | 0.000006 | Spline | 29 |
| Run13.Photo4 | 0.000006 | Spline | 22 |
| Run18.Photo2 | 0.000006 | Spline | 23 |
| Run21.Photo2 | 0.000006 | Spline | 22 |
| Run22.FID11_12 | 0.000006 | Spline | 27 |
| Run22.FID13 | 0.000006 | Spline | 23 |
| Run25.FID1 | 0.000006 | Spline | 25 |
| Run25.Photo2 | 0.000006 | Spline | 20 |
| Run41.Photo1 | 0.000006 | Spline | 20 |
| Run45.Photo1 | 0.000006 | Spline | 21 |
| Run48.FID9 | 0.000006 | Spline | 27 |
| Run50.Photo2 | 0.000006 | Spline | 15 |
| Run58.Photo2 | 0.000006 | Spline | 25 |
| Run62.Photo2 | 0.000006 | Spline | 30 |
| Run69.Photo2 | 0.000006 | Spline | 35 |
| Run70.Photo2 | 0.000006 | Spline | 29 |
| Run72.Photo2 | 0.000006 | Spline | 24 |
| Run74.FID2 | 0.000006 | Spline | 26 |
| Run84.Photo2 | 0.000006 | Spline | 23 |
| Run86.Photo2 | 0.000006 | Spline | 24 |
| Run89.FID3 | 0.000006 | Spline | 24 |
| Run96.Photo6 | 0.000006 | Spline | 29 |
| Run97.Photo1 | 0.000006 | Spline | 27 |
| Run97.Photo2 | 0.000006 | Spline | 33 |
| Run99.FID5 | 0.000006 | Spline | 22 |
| Run4.Photo1 | 0.000005 | Spline | 19 |
| Run4.Photo2 | 0.000005 | Spline | 18 |
| Run8.Photo1 | 0.000005 | Spline | 27 |
| Run10.Photo1 | 0.000005 | Spline | 23 |
| Run22.Photo1 | 0.000005 | Spline | 23 |
| Run28.FID5_FID6 | 0.000005 | Spline | 20 |
| Run28.FID8 | 0.000005 | Spline | 22 |
| Run30.FID1 | 0.000005 | Spline | 26 |
| Run37.Photo5 | 0.000005 | Spline | 24 |
| Run40.Photo1 | 0.000005 | Spline | 32 |
| Run40.Photo2 | 0.000005 | Spline | 32 |
| Run41.FID1 | 0.000005 | Spline | 23 |
| Run49.Photo1 | 0.000005 | Spline | 29 |
| Run57.FID1 | 0.000005 | Spline | 20 |
| Run57.FID2 | 0.000005 | Spline | 24 |
| Run57.FID8 | 0.000005 | Spline | 19 |
| Run57.Photo1 | 0.000005 | Spline | 21 |
| Run59.FID2 | 0.000005 | Spline | 22 |
| Run62.FID1 | 0.000005 | Spline | 23 |
| Run67.Photo2 | 0.000005 | Spline | 20 |
| Run68.Photo2 | 0.000005 | Spline | 23 |
| Run74.FID1 | 0.000005 | Spline | 23 |
| Run75.FID1 | 0.000005 | Spline | 21 |
| Run79.FID4 | 0.000005 | Spline | 26 |
| Run79.Photo2 | 0.000005 | Spline | 20 |
| Run84.FID8 | 0.000005 | Spline | 23 |
| Run84.Photo6 | 0.000005 | Spline | 17 |
| Run90.FID7_8 | 0.000005 | Spline | 21 |
| Run90.Photo2 | 0.000005 | Spline | 18 |
| Run96.FID6 | 0.000005 | Spline | 23 |
| Run96.Photo3 | 0.000005 | Spline | 26 |
| Run99.Photo1 | 0.000005 | Spline | 22 |
| Run9.Photo1 | 0.000004 | Spline | 22 |
| Run12.FID1 | 0.000004 | Spline | 22 |
| Run13.FID15 | 0.000004 | Spline | 32 |
| Run20.FID1 | 0.000004 | Spline | 22 |
| Run25.Photo1 | 0.000004 | Spline | 21 |
| Run33.FID3 | 0.000004 | Spline | 24 |
| Run37.FID3 | 0.000004 | Spline | 19 |
| Run37.FID5 | 0.000004 | Spline | 21 |
| Run37.FID8 | 0.000004 | Spline | 20 |
| Run37.Photo10 | 0.000004 | Spline | 24 |
| Run44.FID2 | 0.000004 | Spline | 25 |
| Run44.Photo2 | 0.000004 | Spline | 30 |
| Run45.FID8 | 0.000004 | Spline | 27 |
| Run47.FID2 | 0.000004 | Spline | 20 |
| Run48.FID2 | 0.000004 | Spline | 22 |
| Run48.Photo2 | 0.000004 | Spline | 25 |
| Run52.FID1 | 0.000004 | Spline | 20 |
| Run53.Photo1 | 0.000004 | Spline | 22 |
| Run54.FID1 | 0.000004 | Spline | 17 |
| Run59.FID3 | 0.000004 | Spline | 18 |
| Run61.FID4 | 0.000004 | Spline | 26 |
| Run61.Photo2 | 0.000004 | Spline | 27 |
| Run65.FID1 | 0.000004 | Spline | 26 |
| Run75.Photo1 | 0.000004 | Spline | 19 |
| Run79.FID2 | 0.000004 | Spline | 30 |
| Run80.Photo2 | 0.000004 | Spline | 18 |
| Run84.FID6 | 0.000004 | Spline | 16 |
| Run88.FID2 | 0.000004 | Spline | 24 |
| Run88.Photo2 | 0.000004 | Spline | 22 |
| Run89.Photo1 | 0.000004 | Spline | 30 |
| Run98.FID1 | 0.000004 | Spline | 14 |
| Run99.Photo4 | 0.000004 | Spline | 23 |
| Run100.FID2 | 0.000004 | Spline | 17 |
| Run6_Photo2 | 0.000003 | Spline | 13 |
| Run7.FID2 | 0.000003 | Spline | 24 |
| Run9.Photo2 | 0.000003 | Spline | 27 |
| Run11.Photo2 | 0.000003 | Spline | 28 |
| Run13.FID3_FID4 | 0.000003 | Spline | 31 |
| Run13.FID7 | 0.000003 | Spline | 29 |
| Run14.FID3 | 0.000003 | Spline | 17 |
| Run18.Photo1 | 0.000003 | Spline | 30 |
| Run23.FID1 | 0.000003 | Spline | 21 |
| Run23.Photo1 | 0.000003 | Spline | 24 |
| Run23.Photo2 | 0.000003 | Spline | 26 |
| Run29.Photo1 | 0.000003 | Spline | 34 |
| Run37.FID2 | 0.000003 | Spline | 15 |
| Run37.Photo9 | 0.000003 | Spline | 19 |
| Run45.FID5 | 0.000003 | Spline | 22 |
| Run48.FID7_8 | 0.000003 | Spline | 16 |
| Run50.Photo1 | 0.000003 | Spline | 23 |
| Run51.FID1 | 0.000003 | Spline | 16 |
| Run51.Photo1 | 0.000003 | Spline | 23 |
| Run52.Photo2 | 0.000003 | Spline | 16 |
| Run54.Photo1 | 0.000003 | Spline | 21 |
| Run56.FID1 | 0.000003 | Spline | 18 |
| Run57.FID3 | 0.000003 | Spline | 31 |
| Run59.FID4 | 0.000003 | Spline | 21 |
| Run61.FID3 | 0.000003 | Spline | 34 |
| Run61.Photo1 | 0.000003 | Spline | 28 |
| Run62.Photo1 | 0.000003 | Spline | 22 |
| Run63.Photo2 | 0.000003 | Spline | 20 |
| Run64.FID1 | 0.000003 | Spline | 27 |
| Run65.FID2 | 0.000003 | Spline | 23 |
| Run66.Photo2 | 0.000003 | Spline | 12 |
| Run67.FID1 | 0.000003 | Spline | 19 |
| Run67.FID2 | 0.000003 | Spline | 24 |
| Run71.Photo1 | 0.000003 | Spline | 23 |
| Run71.Photo2 | 0.000003 | Spline | 24 |
| Run73.Photo1 | 0.000003 | Spline | 28 |
| Run80.Photo1 | 0.000003 | Spline | 15 |
| Run81.Photo1 | 0.000003 | Spline | 25 |
| Run84.FID1_2_3 | 0.000003 | Spline | 15 |
| Run84.Photo5 | 0.000003 | Spline | 18 |
| Run90.FID2 | 0.000003 | Spline | 17 |
| Run95.FID3 | 0.000003 | Spline | 16 |
| Run96.FID3 | 0.000003 | Spline | 27 |
| Run98.Photo1 | 0.000003 | Spline | 18 |
| Run99.FID1_FID2 | 0.000003 | Spline | 29 |
| Run100.Photo2 | 0.000003 | Spline | 15 |
| Run7.FID3 | 0.000002 | Spline | 26 |
| Run7.Photo1 | 0.000002 | Spline | 35 |
| Run20.Photo2 | 0.000002 | Spline | 28 |
| Run21.FID5 | 0.000002 | Spline | 20 |
| Run24.FID1 | 0.000002 | Spline | 27 |
| Run28.FID1 | 0.000002 | Spline | 16 |
| Run30.Photo1 | 0.000002 | Spline | 29 |
| Run33.FID1 | 0.000002 | Spline | 21 |
| Run36.Photo1 | 0.000002 | Spline | 22 |
| Run37.FID1 | 0.000002 | Spline | 17 |
| Run37.FID7 | 0.000002 | Spline | 19 |
| Run37.FID9 | 0.000002 | Spline | 17 |
| Run37.Photo1 | 0.000002 | Spline | 18 |
| Run37.Photo2 | 0.000002 | Spline | 23 |
| Run37.Photo6 | 0.000002 | Spline | 30 |
| Run44.FID1 | 0.000002 | Spline | 23 |
| Run44.FID3 | 0.000002 | Spline | 28 |
| Run45.FID2 | 0.000002 | Spline | 28 |
| Run45.FID4 | 0.000002 | Spline | 19 |
| Run46.Photo2 | 0.000002 | Spline | 29 |
| Run48.FID5 | 0.000002 | Spline | 23 |
| Run53.FID1 | 0.000002 | Spline | 20 |
| Run56.Photo1 | 0.000002 | Spline | 22 |
| Run57.FID5 | 0.000002 | Spline | 25 |
| Run57.FID7 | 0.000002 | Spline | 19 |
| Run59.Photo2 | 0.000002 | Spline | 20 |
| Run60.FID2 | 0.000002 | Spline | 20 |
| Run63.Photo1 | 0.000002 | Spline | 27 |
| Run64.Photo2 | 0.000002 | Spline | 20 |
| Run65.FID4 | 0.000002 | Spline | 27 |
| Run68.Photo1 | 0.000002 | Spline | 21 |
| Run72.Photo1 | 0.000002 | Spline | 19 |
| Run76.Photo1 | 0.000002 | Spline | 19 |
| Run79.Photo1 | 0.000002 | Spline | 22 |
| Run79.Photo3 | 0.000002 | Spline | 18 |
| Run82.FID3 | 0.000002 | Spline | 20 |
| Run82.Photo2 | 0.000002 | Spline | 20 |
| Run84.FID7 | 0.000002 | Spline | 27 |
| Run84.Photo1 | 0.000002 | Spline | 17 |
| Run85.FID1 | 0.000002 | Spline | 24 |
| Run93.Photo1 | 0.000002 | Spline | 17 |
| Run95.FID2 | 0.000002 | Spline | 18 |
| Run95.Photo1 | 0.000002 | Spline | 14 |
| Run96.FID1 | 0.000002 | Spline | 25 |
| Run96.FID2 | 0.000002 | Spline | 26 |
| Run98.Photo2 | 0.000002 | Spline | 13 |
| Run98.Photo4 | 0.000002 | Spline | 17 |
| Run5.Photo2 | 0.000001 | Spline | 18 |
| Run13.FID5 | 0.000001 | Spline | 32 |
| Run14.FID2 | 0.000001 | Spline | 23 |
| Run21.FID2 | 0.000001 | Spline | 23 |
| Run22.Photo2 | 0.000001 | Spline | 29 |
| Run28.Photo1 | 0.000001 | Spline | 22 |
| Run28.Photo2 | 0.000001 | Spline | 22 |
| Run33.Photo2 | 0.000001 | Spline | 26 |
| Run36.Photo2 | 0.000001 | Spline | 37 |
| Run37.FID11 | 0.000001 | Spline | 16 |
| Run37.FID6 | 0.000001 | Spline | 21 |
| Run37.Photo7 | 0.000001 | Spline | 19 |
| Run38.Photo1 | 0.000001 | Spline | 31 |
| Run44.Photo1 | 0.000001 | Spline | 19 |
| Run45.FID9 | 0.000001 | Spline | 23 |
| Run45.Photo2 | 0.000001 | Spline | 19 |
| Run46.FID1 | 0.000001 | Spline | 19 |
| Run51.Photo2 | 0.000001 | Spline | 18 |
| Run55.Photo1 | 0.000001 | Spline | 19 |
| Run60.Photo2 | 0.000001 | Spline | 20 |
| Run61.FID1 | 0.000001 | Spline | 22 |
| Run64.Photo1 | 0.000001 | Spline | 19 |
| Run65.Photo1 | 0.000001 | Spline | 18 |
| Run66.Photo1 | 0.000001 | Spline | 16 |
| Run70.Photo1 | 0.000001 | Spline | 15 |
| Run75.FID4 | 0.000001 | Spline | 22 |
| Run75.FID5 | 0.000001 | Spline | 17 |
| Run75.FID6 | 0.000001 | Spline | 17 |
| Run75.Photo2 | 0.000001 | Spline | 20 |
| Run76.FID1 | 0.000001 | Spline | 17 |
| Run76.Photo2 | 0.000001 | Spline | 19 |
| Run79.Photo4 | 0.000001 | Spline | 18 |
| Run82.FID2 | 0.000001 | Spline | 16 |
| Run82.Photo1 | 0.000001 | Spline | 24 |
| Run83.Photo2 | 0.000001 | Spline | 16 |
| Run84.FID4 | 0.000001 | Spline | 15 |
| Run84.Photo3 | 0.000001 | Spline | 25 |
| Run85.Photo1 | 0.000001 | Spline | 19 |
| Run87.FID1 | 0.000001 | Spline | 18 |
| Run87.Photo2 | 0.000001 | Spline | 17 |
| Run89.FID1 | 0.000001 | Spline | 21 |
| Run89.FID2 | 0.000001 | Spline | 21 |
| Run90.FID1 | 0.000001 | Spline | 17 |
| Run90.FID3 | 0.000001 | Spline | 22 |
| Run90.FID4 | 0.000001 | Spline | 17 |
| Run93.FID1 | 0.000001 | Spline | 19 |
| Run93.Photo2 | 0.000001 | Spline | 18 |
| Run95.FID1 | 0.000001 | Spline | 13 |
| Run95.Photo2 | 0.000001 | Spline | 12 |
| Run96.FID4 | 0.000001 | Spline | 29 |
| Run98.FID2 | 0.000001 | Spline | 20 |
| Run98.Photo3 | 0.000001 | Spline | 19 |
| Run99.FID3_FID4 | 0.000001 | Spline | 21 |
| Run99.Photo3 | 0.000001 | Spline | 14 |
| Run100.FID1 | 0.000001 | Spline | 14 |
| Run100.FID3 | 0.000001 | Spline | 18 |
| Run100.Photo1 | 0.000001 | Spline | 17 |
